# A simple and broadly applicable nanobody-based approach to generate potent TNFR agonists

**DOI:** 10.1038/s41419-026-08911-x

**Published:** 2026-05-30

**Authors:** Isabell Lang, Olena Zaitseva, Amelie Glöckler, Daniela Siegmund, Dalia Sheta, Bayan Mouhandes, Daniela Weisenberger, Viktoria Schäfer, Svetlana Stepanzow, Theresa Schneider, Andreas Beilhack, Alexander Crauel, Markus Kilisch, Lisa-Marie Funk, Hansjörg Götzke, Steffen Frey, Harald Wajant

**Affiliations:** 1 Division of Molecular Internal Medicine, Department of Internal Medicine II, University Hospital Würzburg, Würzburg, Germany; 2 Department of Internal Medicine II, Interdisciplinary Center for Clinical Research (IZKF) laboratory Würzburg, Center for Experimental Molecular Medicine, University Hospital Würzburg, Würzburg, Germany; 3 NanoTag Biotechnologies GmbH, Göttingen, Germany

**Keywords:** Biologics, Cell death and immune response

## Abstract

The receptors of the tumor necrosis factor (TNF) receptor superfamily (TNFRSF) are of overwhelming scientific and clinical relevance and stand at the center of intensive basic and translational research efforts. TNFRSF receptors (TNFRs) are engaged by membrane-bound ligands of the TNF superfamily (TNFSF) and, in some cases, by soluble ligand molecules released from the membrane-bound TNFSF ligand (TNFL) molecules. The development of recombinant TNFL-based TNFR agonists for research and especially therapeutic purposes is highly “individualized”, as ligand type-specific hurdles must be overcome in terms of stability, manufacturability, TNFR-specificity and need for oligomerization. TNFR-specific antibodies can also show agonistic activity, but this agonism typically requires FcγR-binding, resulting in a reciprocal conditional bispecific FcγR/TNFR agonism not useful for the study or exploitation of pure TNFR agonism. Some antibodies trigger intrinsic TNFR agonism independent from FcγR-binding, but the rational development of such antibodies is poorly predictable and furthermore challenging due to isotype- and epitope-requirements and poor specific activity when benchmarked with FcγR-bound anti-TNFR antibodies.

Using a series of nanobodies (or single-domain antibodies (sdAbs) or variable heavy domain of heavy chains (VHHs)) specific for the TNFRSF members 41BB, BCMA, CD40, CD95, TRAILR2/DR5, GITR, OX40, TNFR1 and TNFR2, we show here that genetic fusion of single-chain encoded triplets of these nanobodies with oligomerizing protein scaffolds regularly results in potent hexa-, nona- and dodecavalent agonists inducing TNFR signaling with EC50-values in the sub-nanomolar range. The oligovalent nanobody formats described exhibit superior CMC properties and enable the simple generation of highly active TNFR agonists from virtually any TNFR-specific nanobody.

## Introduction

The tumor necrosis factor (TNF) superfamily (TNFSF) comprises a family of trimeric type II transmembrane ligands, which are often also found in soluble form and are defined by the phylogenetically conserved TNF homology domain (THD) [1]. The TNFSF ligands (TNFLs) elicit their activities by means of receptors of the TNF receptor superfamily (TNFRSF). TNFRSF receptors (TNFRs) and TNFLs are crucially involved in the regulation of a variety of immunological processes, but also control cellular differentiation and cell survival [2]. Accordingly, TNFLs and TNFRs are important targets for therapeutic approaches ranging from the treatment of autoinflammatory diseases to the therapy of heart-related diseases to cancer immunotherapy. TNFRs are characterized by the cysteine-rich domain, a conserved structural module that occurs in one to six copies in the extracellular part of these molecules [2]. The TNFRSF comprises three subgroups: the death receptor and TRAF (TNF receptor-associated factor)-interacting TNFR subgroups and the decoy TNFRs [3]. The latter lack an authentic signaling capacity and instead act by regulating the activity of the signaling competent TNFRs by hetero-complex formation and/or ligand competition [3]. Intriguingly, the signaling-competent TNFRs differ in their reaction towards soluble ligand trimers. Category I TNFRs bind soluble TNFL trimers and elicit similar effects as upon stimulation by the corresponding membrane-bound TNFL species. In contrast, category II TNFRs trigger no or only limited cellular signaling despite high-affinity binding of soluble TNFL trimers [4, 5].

In view of the overwhelming biological and pathophysiological importance of TNFLs and TNFRs, it is no surprise that TNFR agonists are not only important tools for basic research but also attract considerable attention as therapeutic reagents [6–8]. There are two major groups of TNFR agonists, TNFL-based agonists and antibody-based agonists. However, both groups of agonists have limitations, complicating their use as research tools and making their clinical development an elaborate process.

The development of TNFR agonists based on the receptor-binding THD of TNFLs has to cope with various challenges, which are of variable relevance for different TNFL types and which can be solved by different means. The development of TNFL-based TNFR agonists is therefore a highly “individualized” process, which cannot be easily transferred from one TNFL type to the next. Indeed, even agonists using one and the same ligand-derived TNFR binding domain can considerably differ in important properties, including specific activity, CMC properties and pharmacokinetics [9–11]. Furthermore, several TNFLs interact with two or more different TNFR types, e.g., TRAIL, TNF, Baff and LIGHT [2, 7]. Thus, the generation of TNFR type-specific agonists with the help of such ligands requires the identification of mutations conferring receptor specificity. Last, but not least, the in vivo use of TNFL-based agonists is limited due to the poor serum half-life of soluble TNFL trimers, and the productivity of some TNFL constructs is rather poor.

Early on, it was recognized that TNFR-specific antibodies and antisera can also display agonistic activity [3]. Obviously, antibody-based TNFR agonists are attractive as they straightforwardly solve some of the limitations that TNFLs have, such as a lack of specificity for a single TNFR type or poor serum half-life. However, antibody-based agonists must also ultimately trigger TNFR clustering. Indeed, bivalent antibodies specific for category II TNFRs are typically poor agonists and show strong agonism only upon crosslinking with secondary antibodies, an approach early on and broadly used in the TNFR field. Bivalent antibodies can also efficiently activate TNFRs when bound to FcγRs [3, 4, 12]. However, this kind of TNFR agonism is not useful for the study and clinical exploitation of pure TNFR agonism. Recently, it has been further demonstrated that biparatopic tetravalent TNFR-specific antibodies can act as pure TNFR agonists with intrinsic activity, but this generally applicable approach suffers from the need for screening for two different functionally complementing antibodies as building blocks [13]. Thus, there is still considerable need for a simple procedure enabling the generation of potent TNFR agonists. Here, we describe a simple method allowing the generation of highly active TNFR agonists from virtually any TNFR-specific single-domain antibody (sdAb, nanobody, VHH).

## Results

### Bivalent TNFR-targeting nanobody (Nb) fusion proteins are not or only poorly agonistic

**Fig. 1:**
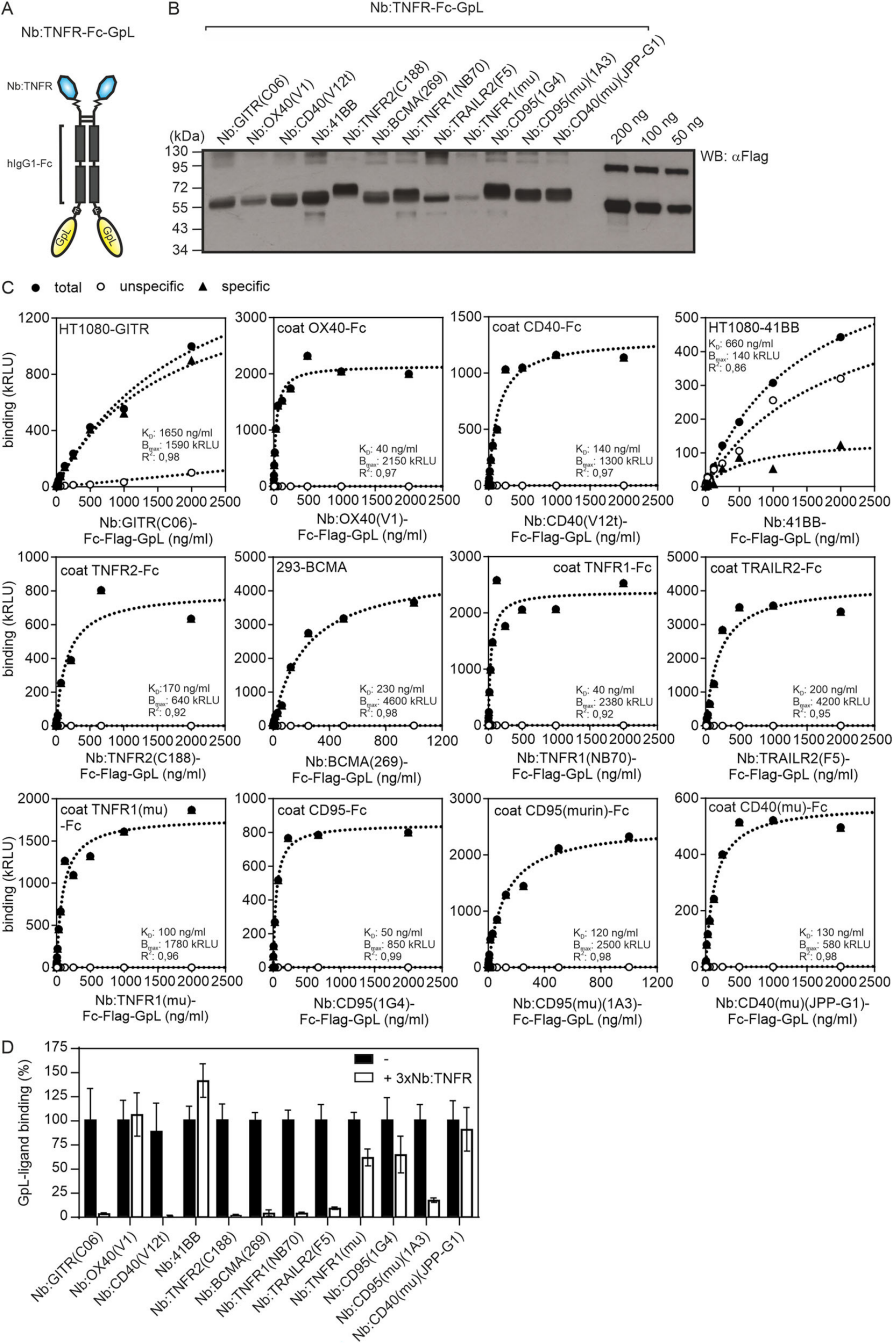
Characterization of bivalent TNFR-targeting nanobody (Nb) fusion proteins. **A** Domain architecture of the Nb:TNFR-Fc-GpL fusion proteins. F, FLAG tag; GpL, *Gaussia princeps* luciferase; Nb:TNFR(ed), TNFR ectodomain-specific nanobody. **B** Western blot analysis of supernatants of HEK293 cells transiently expressing the indicated Nb:TNFR-Fc-GpL fusion proteins with anti-Flag antibody. **C** Binding of Nb:TNFR-Fc-GpL fusion proteins to plastic-immobilized TNFR(ed)-Fc fusion proteins or TNFR-expressing cells. Results of one of two similar experiments are shown. **D** Plastic immobilized TNFR(ed)-Fc fusion proteins or adherent TNFR-expressing cells were treated with 5 µg/ml of GpL-free oligovalent Nb:TNFR fusion proteins in triplicate. Then, 20 ng/ml of GpL-TNFL fusion proteins were added, and finally, cell-associated GpL activity was measured. One experiment with technical triplicates is shown (mean ± SD).

Bivalent IgG antibodies targeting TNFRs, particularly those of the category II, often show no or only limited agonistic activity unless they are presented by cells in FcγR-bound form. We wondered whether this also applies to bivalent nanobody fusion proteins. To address this issue, we generated and analyzed a panel of bivalent Nb-Fc fusion proteins targeting 41BB, BCMA, CD40, muCD40, CD95, muCD95, GITR, OX40, TNFR1, muTNFR1, TNFR2 and TRAILR2 (Table 1). To facilitate the basic characterization of these constructs, we equipped the Nb:TNFR-Fc fusion proteins with a C-terminal *Gaussia princeps* luciferase (GpL) reporter domain. In cell-free binding assays with plastic-immobilized TNFR ectodomain Fc fusion proteins, the Nb:TNFR-Fc-GpL variants showed apparent affinities between 40 and 1650 ng/ml (Fig. 1A–C and Table 1). The nanobody panel used comprised both nanobodies blocking ligand binding and nanobodies not interfering with ligand binding (Fig. 1D and Table 1).

**Table 1:** Characterization of TNFR-specific nanobodies used in this study.

Nanobody (clone)	Specificity	Sequence source	K_D_ of Fc-GpL variant (ng/ml)	Ligand blocking
	41BB	Zhai et al., [56]	660	No
269A37948	BCMA	US 20220127371A1	230	Yes
V12t	CD40	De Weerdt et al., [15]	140	Yes
JPP-G1	muCD40	WO 2018201017A1	130	No
1G4	CD95	this study	50	Weak
1A3	muCD95	this study	120	Yes
hzC06	GITR	US 2019100594A1	1650	Yes
1D10V1	OX40	AU 2019321490A1	40	No
Nb:70	TNFR1	Steeland et al., [57]	40	Yes
DOM1m-21-23	muTNFR1	Goodall et al., [58]	100	Weak
C188	TNFR2	WO 2022207921	170	Yes
F5	TRAILR2	WO 2017011837A2	200	Yes

**Fig. 2:**
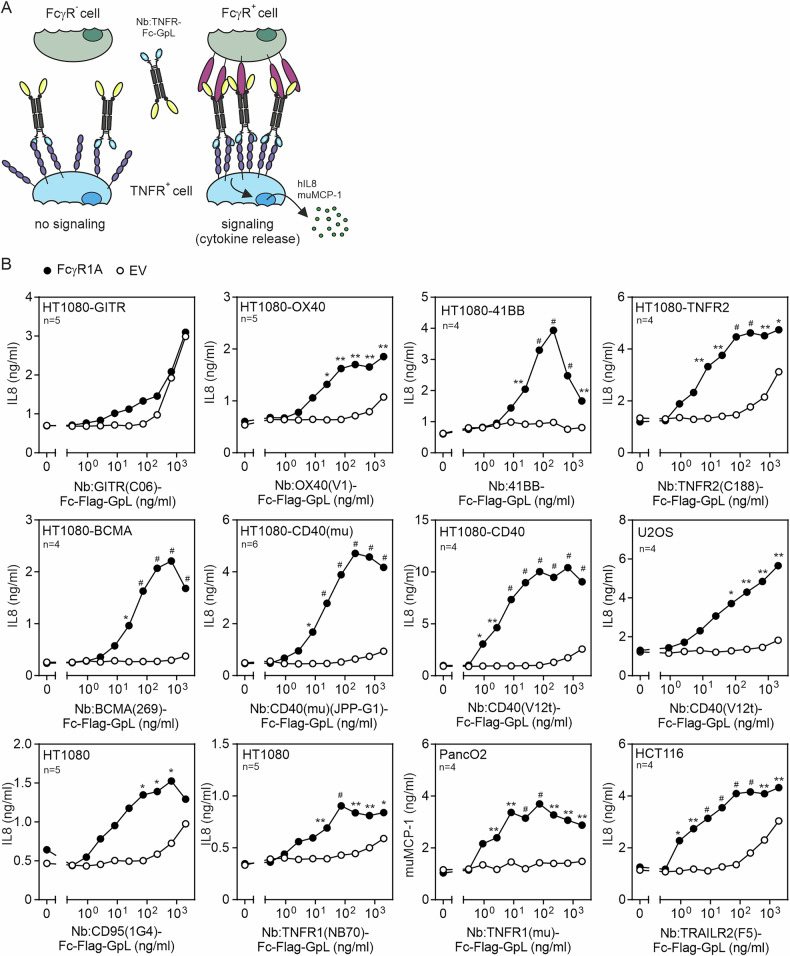
Bivalent TNFR-targeting nanobody (Nb) fusion proteins are largely non-agonistic. **A** Scheme of assay for analyzing the agonism of free versus FcγR-bound Nb:TNFR(ed)-Fc-GpL fusion proteins. **B** The indicated cells (10^4^ per well) responding with IL8 or muMCP-1 production to TNFR stimulation were cultured in 96-well plates overnight and supplemented the next day (1:1) with HEK293 cells transfected with empty vector (EV) or an expression plasmid encoding FcγR1A along with increasing concentrations of the indicated bivalent Nb:TNFR(ed)-Fc-GpL constructs. The following day, the amount of IL8 (or muMCP-1) in the cell culture supernatants was determined. Upregulation of IL8 or muMCP-1 production served as a readout for TNFR activation. Where indicated, cell cultures were supplemented with 20 µM ZVAD to prevent inhibitory effects of death receptor-induced apoptosis on IL8 production. Please note that the different TNFR responder cell lines used elicit different maximum IL8 production in response to TNFR stimulation. Shown are the averaged data, and the number of independent experiments is noted in the corresponding graph. Data were analyzed with the GraphPad Prism software using the multiple unpaired t-test. * *p* < 0.05; **p < 0.01; # *p* < 0.0001.

To evaluate the impact of FcγR-binding on the TNFR-stimulating activity of the various Nb:TNFR-Fc-GpL fusion proteins, we analyzed their TNFR agonism in cocultures of TNFR responder cells with either empty vector (EV)- or human FcγR1A-transfected HEK293 cells (Fig. 2A). All signaling-competent receptors of the TNFRSF activate the classical NFκB pathway. The latter results in strong transcription and production of the IL8/CXCL8 gene in many human cell lines [14] and of MCP-1 in murine cell lines. HT1080-GITR, HT1080-OX40, HT1080-41BB, HT1080-TNFR2, HT1080-BCMA and HT1080-muCD40 transfectants served as responder cells for GITR, OX40, 41BB, TNFR2, BCMA and murine CD40. The CD40 response was analyzed with the help of HT1080-CD40 transfectants and U2OS cells, which have endogenous CD40 expression. Furthermore, HT1080 cells were used as TNFR1 and CD95 responder cells, HCT116 cells as TRAILR2 responders and PancO2 cells as murine TNFR1 responder cells. In all these responder cells, robust production of IL8 or muMCP-1 occurs upon TNFR engagement. HEK293 cells elicit comparatively poor IL8 expression and lack expression of most of the TNFRs investigated (Supplementary Fig. S1). In the case of the murine TNFR-interacting Nb:TNFR-Fc-GpL fusion proteins, murine cell lines were used as responder cells, and muMCP-1 served as read-out. In the cocultures with EV-transfected HEK293 cells, Nb:TNFR-Fc-GpL fusion proteins elicited no, or at best minimal, IL8 induction up to a concentration of 2 µg/ml. In contrast, in the cocultures with FcγR1A-transfected HEK293 cells, all constructs with exception of Nb:GITR-Fc-GpL triggered a significant IL8 response already at low concentrations in the range of 1 and 20 ng/ml (app. 10-200 pM; Fig. 2B). Thus, FcγR1A-binding lowers the EC50-values for IL8 induction of the bivalent Nb:TNFR molecules by several orders of magnitude (Fig. 2B). In sum, these data revealed that bivalent Nb-Fc fusion proteins targeting TNFRs act largely similar to conventional bivalent anti-TNFR IgGs, thus having per se no, or poor intrinsic agonism but also possessing a latent capacity to trigger robust TNFR agonism when bound to FcγRs.

### Oligovalent Nb:TNFR fusion proteins exhibit strong intrinsic agonism

**Fig. 3:**
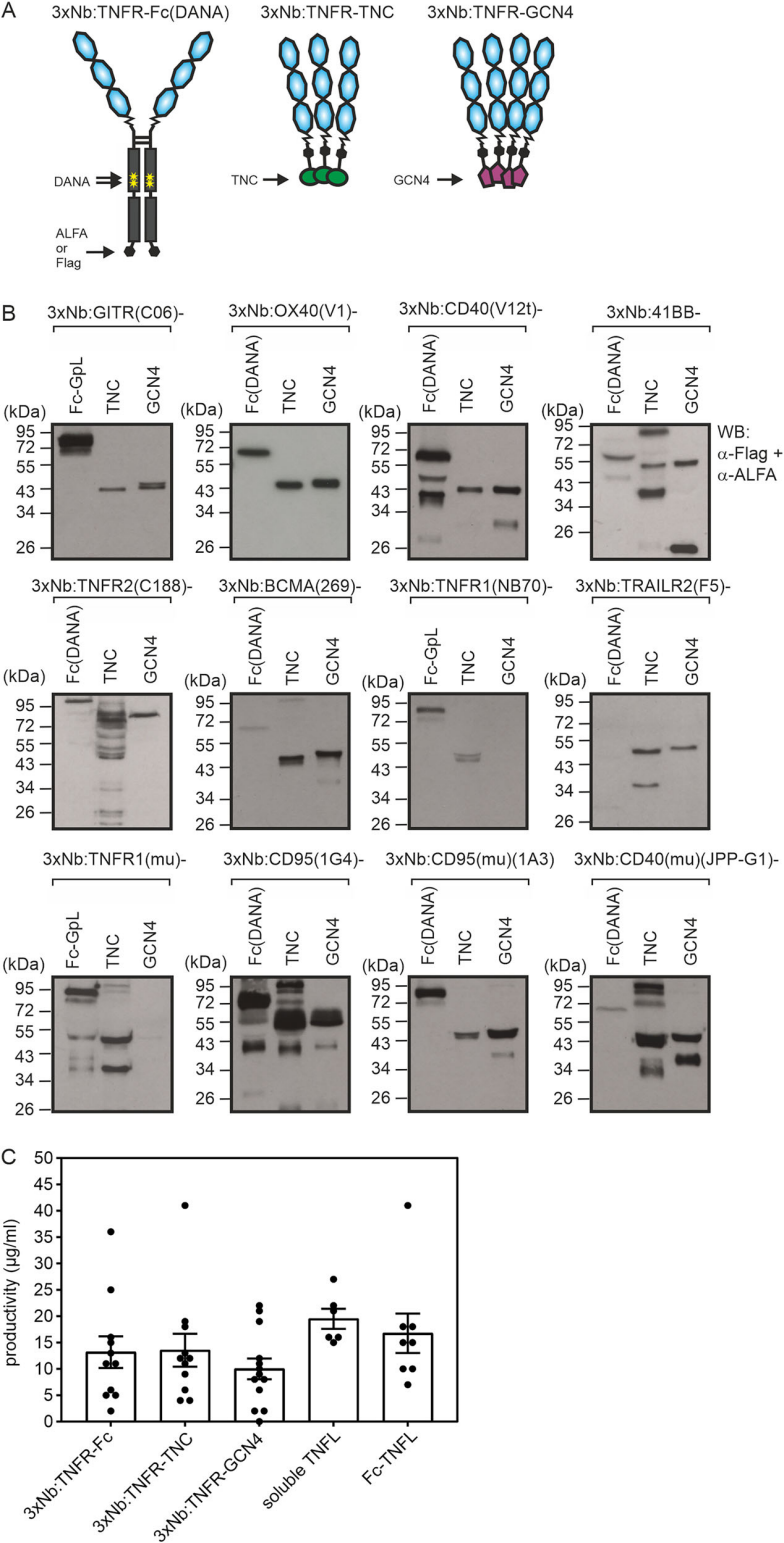
Characterization of oligovalent TNFR-targeting nanobody (Nb) fusion proteins. **A** Scheme of oligovalent TNFR(ed)-specific nanobody fusion proteins. **B** Supernatants of cells producing TNFR(ed)-specific oligovalent Nb fusion proteins were separated by reducing SDS-PAGE and analyzed by western blotting. **C** The 3xNb:TNFR-Fc, 3xNb:TNFR-TNC and 3xNb:TNFR-GCN4 variants, Flag-tagged soluble TNFL constructs (41BB-L, APRIL, CD95L, muCD95L, muCD40L, TNF) and Fc-TNFL constructs (41BB-L, APRIL, CD40L, CD95L, GITRL, OX40L, TNF, TRAIL) were transiently expressed using HEK293 cells. For each protein, productivity was determined in three independent transfection experiments by western blotting. Averaged productivities of each protein were assigned to the different protein categories and analyzed by ANOVA. There was no statistically significant difference in the productivity between any of the protein categories. The productivities of the individual proteins are listed in Supplemental Table S1.

We recently observed that oligovalent fusion proteins of the CD40-specific nanobody (Nb) V12 [15] with six or more Nb domains regularly display excellent intrinsic agonism, high productivity and no aggregation [16]. Therefore, we verified the hypothesis that oligovalency is a general exploitable principle enabling the construction of TNFR agonists with high intrinsic activity from TNFR-specific nanobodies. To test this idea, we generated hexa-, nona-, and dodecavalent Nb fusion proteins of the TNFR-specific Nb panel described above. To obtain the oligovalent Nb constructs, we genetically fused single-chain-encoded Nb:TNFR trimers (3xNb:TNFR) to dimerizing, trimerizing and tetramerizing protein domains. For dimerization of the 3xNb:TNFR domains, thus to achieve hexavalency, we used the human IgG1 Fc domain as a fusion partner (Fig. 3A). To reduce the potential interaction with FcγRs, we furthermore introduced the D265A/N297A (DANA) mutation into the Fc domain [17]. The nonavalent Nb:TNFR variants were furthermore generated by fusing the 3xNb:TNFR domains with the short trimerization domain of tenascin-C (TNC), and the dodecavalent Nb:TNFR variants were realized by fusing the 3xNb:TNFR domains with a parallel, four-stranded coiled coil tetramer-forming leucine zipper domain of the yeast transcription factor GCN4 [18, 19] (Fig. 3A). With few exceptions, all the oligovalent Nb:TNFR fusion proteins were produced in good to excellent yields (Fig. 3B and Supplementary Table 1). There was no significant correlation between domain architecture and productivity of the 3xNb:TNFR fusion protein types (Fig. 3C). The expression levels of the 3xNb:TNFR fusion proteins were furthermore comparable to those of various types of TNFL fusion proteins, Flag-tagged soluble TNFLs and Fc-TNFL fusion proteins (Fig. 3C and Supplementary Table 1). In the majority of cases, the proteins migrated in reducing SDS-PAGE as single bands roughly corresponding in size to the estimated size of their protomers. In some cases, there was an additional faster migrating band which presumably lacks one of the three Nb domains due to cleavage/breaks in the linker sequences connecting the latter. A few of the 3xNb:TNFR-TNC fusion proteins showed a high molecular weight band presumably reflecting non-resolved 3xNb:TNC trimers (Fig. 3B). To furthermore investigate the formation of disulfide bridges between the protomers, we analyzed cell culture supernatants of the different constructs under reducing and non-reducing conditions using western blotting (WB). The 3xNb:TNFR-Fc(DANA) fusion proteins migrated under non-reducing conditions largely as dimers and under reducing conditions mainly as monomers (Supplementary Fig. S2). The 3xNb:TNFR-GCN4 fusion proteins migrated according to the size of their protomers, irrespective of reduction with β-mercaptoethanol. Since the GCN4 tetramerization domain is from an intracellular protein, the absence of disulfide bridges is to be expected. The Nb fusion proteins with the trimerization domain of tenascin-C, a molecule of the extracellular matrix, migrated according to the size of the individual protomers under reducing conditions and largely also under non-reducing conditions. Apparently, the 3xNb:TNFR-TNC protomers are therefore only partially linked via disulfide bridges. However, it is known that tenascin-C forms very stable, non-covalent trimers even with only partial disulfide bond formation [18].

**Fig. 4:**
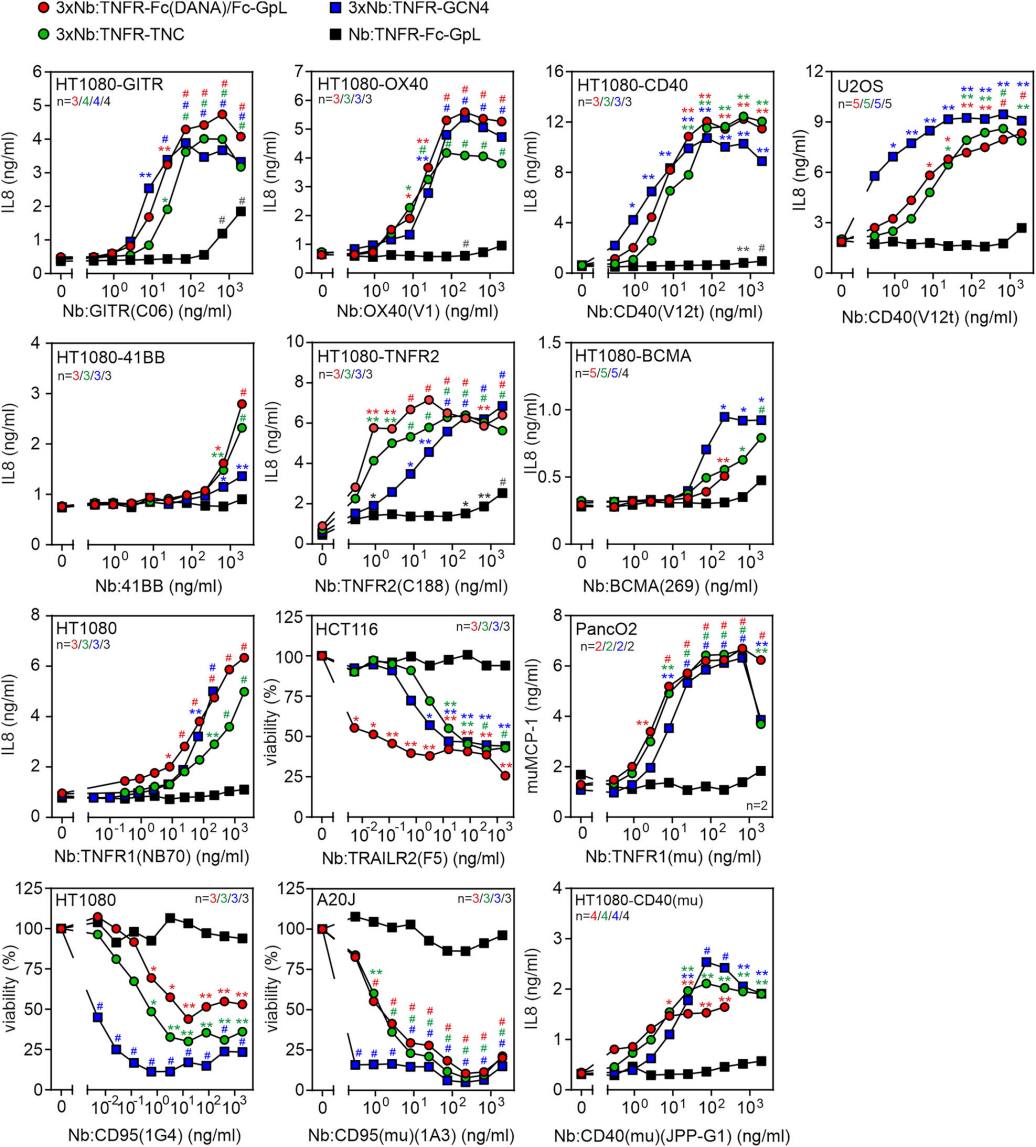
Oligovalent Nb:TNFR fusion proteins exhibit strong intrinsic agonism. The indicated cell lines responding with IL8 or muMCP-1 production or cell death to TNFR stimulation were challenged overnight with cell culture supernatants containing the indicated oligovalent Nb:TNFR fusion proteins. Next day, cells were analyzed for IL8 or muMCP-1 production by ELISA or for cellular viability. HT1080, HCT116 and A20J cells were sensitized for cell death induction with 2.5 µg/ml CHX. Number of independent experiments is noted in the corresponding graph and color. Shown are averaged data which were analyzed using ordinary one-way ANOVA. Construct-treated groups were compared with the untreated control. *p < 0.05; **p < 0.01; # *p* < 0.0001.

To evaluate the receptor-stimulating activity of the various oligovalent Nb:TNFR fusion proteins, we again analyzed IL8-induction, or, in the case of the Nb:muTNFR1 fusion proteins, muMCP-1-induction. In the case of the death receptor-targeting constructs, we analyzed cell death induction. All oligovalent Nb:TNFR molecules, with exception of the 3xNb:41BB variants, revealed robust agonism starting at concentration between 1 and 20 ng/ml (Fig. 4). The bivalent variants as benchmarks showed again no activity or at best residual activity (Fig. 4). With exception of the BCMA-, TRAILR2-, muCD95- and CD95-specific panels, the hexa-, nona- and dodecavalent variants of a 3xNb:TNFR fusion protein panel showed similar maximal IL8 induction and almost similar EC50-values in the range of 1–10 ng/ml (app. 10-100 pM; Fig. 4 and Supplementary Table S2). In the case of the BCMA-, muCD95- and CD95-specific Nb:TNFR fusion protein panels, the dodecavalent 3xNb:TNFR-GCN4 variants triggered half-maximal TNFR responses already at one to two orders of magnitude lower concentrations than the corresponding hexa- and nonavalent variants (Fig. 4). In the case of TRAILR2, the hexavalent 3xNb:TRAILR2-Fc(DANA) triggered half-maximal response up to two orders of magnitude lower concentration. Notably, the EC50-values of the dodecavalent CD95-specific nanobodies were below 10 pg/ml, thus in the sub-picomolar range (Fig. 4). The 41BB-specific oligovalent Nb fusion proteins induced IL8 only at higher concentrations of > 100 ng/ml and were thus clearly less agonistic than the other constructs and the FcγR-bound Nb:41BB-Fc dimer (Fig. 4). For TNFR2 and CD40, we also generated and tested oligovalent constructs derived from other nanobody clones and achieved similar results as with the constructs shown in Fig. 4**(**Supplementary Fig. S3). Five of the 24 oligovalent 3xNb fusion proteins targeting the TNFRs engaged by the commercial benchmarks *Mega*CD40L, *Mega*CD95L, *Mega*TNF, Fc-OX40L and GITRL-His showed a slightly lower “specific activity” than these benchmarks (Supplementary Table S2). Specific activity is hereby defined here as the ratio of the EC50 value of the benchmark to the EC50 value of the Nb construct of interest. The other 19 constructs, however, showed an up to > 200 times higher “specific activity.” With exception of the 3xNb:TNFR fusion proteins derived of the Nb:CD40(1B6), which are all proved to be similarly active as the benchmark *Mega*CD40L, at least one of the three oligovalent 3xNb:TNFR fusion protein variants targeting the same TNFR showed a “specific activity” that was at least one, and usually two, orders of magnitude higher than the corresponding benchmark (Supplementary Table S2).

Nanobodies and nanobody fusion proteins are typically characterized by good stability [20]. Therefore, we compared the temperature stability of the hexa-, nona-and dodecavalent 3xNb:TNFR fusion proteins with that of corresponding Fc-TNFLs using four TNFRs, CD95, OX40, CD40 and TNFR2 as examples. The 3xNb:TNFR fusion proteins showed no or only a slight reduction in activity after incubation at 60°C for 1 h or prolonged incubation at 37 °C (Supplementary Fig. S4). In contrast, the temperature stability of the four Fc-TNFLs varied considerably. While Fc-CD95L and TNF proved to be similarly temperature-stable as the 3xNb:CD95 and 3xNb:TNFR1 fusion proteins, Fc-CD40L and Fc-OX40L lost considerable activity after 1 hour of incubation at 60 °C and longer incubation at 37 °C (Supplementary Fig. S4).

Taken together, the oligovalent 3xNb:TNFR agonists show good producibility, high stability and, most important, much higher activity than TNFL-based TNFR agonists.

### Purification and benchmarking of selected oligovalent Nb:TNFR fusion proteins

**Fig. 5:**
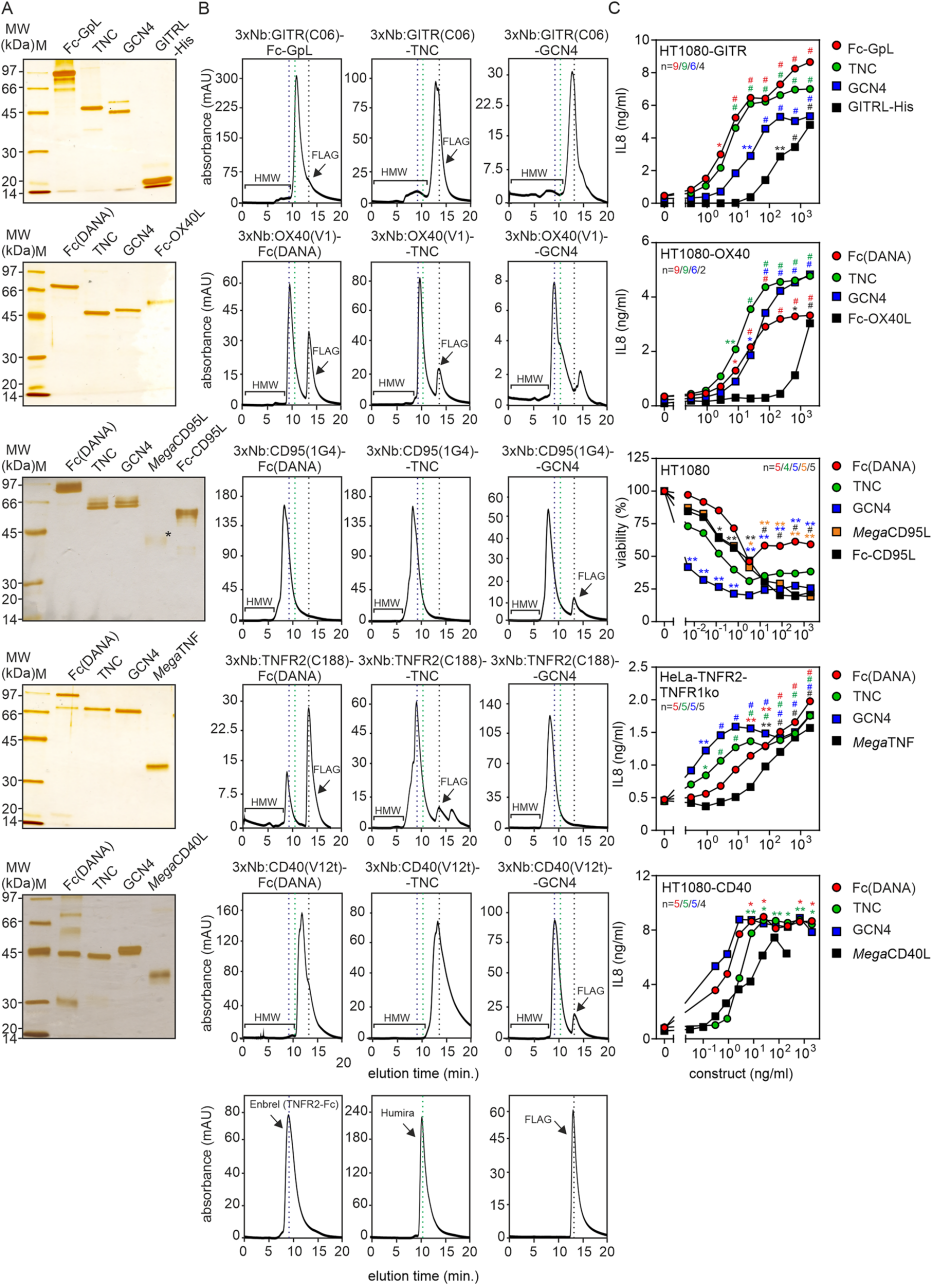

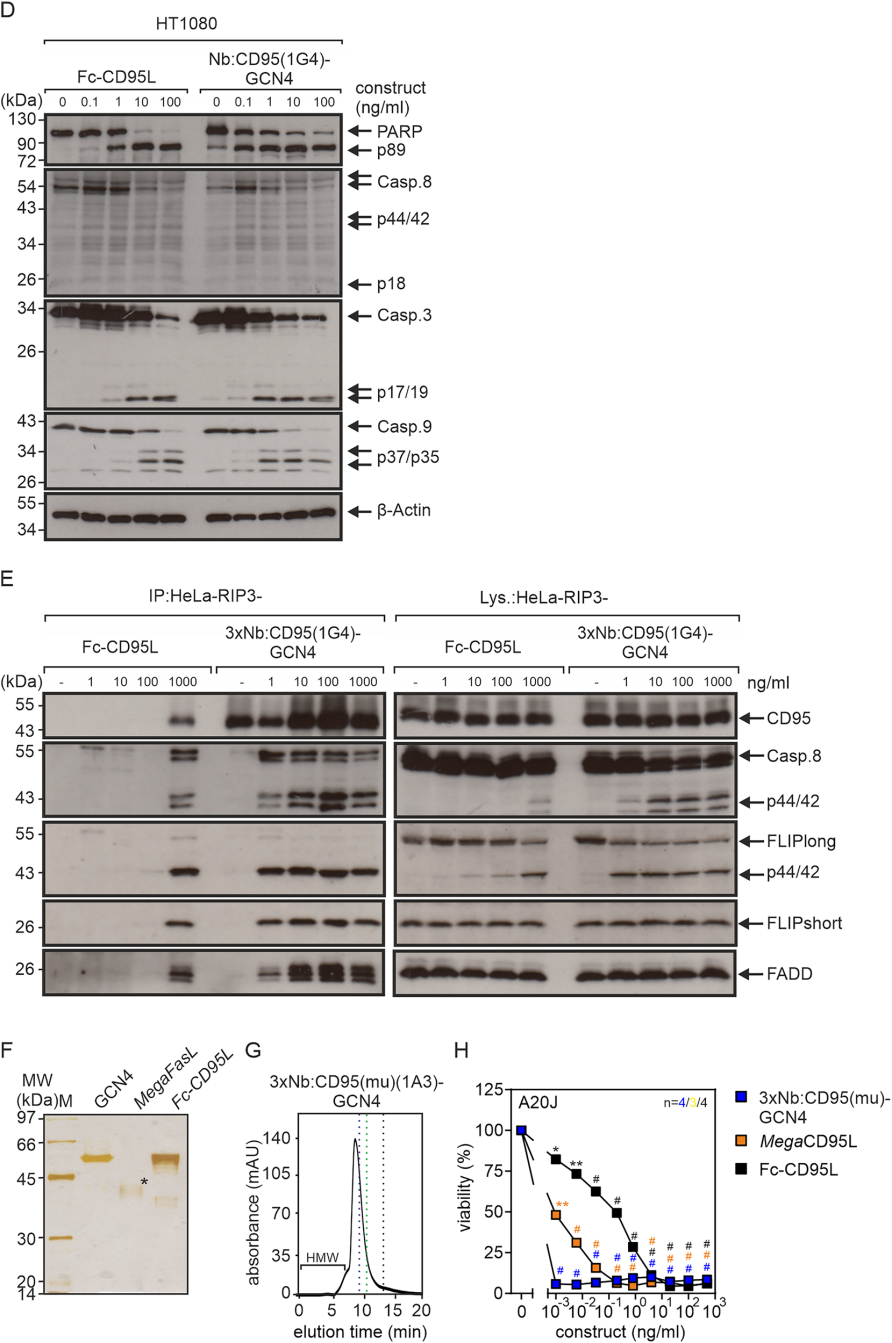
Purification and benchmarking of selected oligovalent Nb:TNFR fusion proteins. The indicated fusion proteins were purified by affinity chromatography using anti-Flag agarose or ALFA Selector^CE^ resin. Purified proteins were analyzed by SDS-PAGE analysis (* the amount of *Mega*CD95L applied to the gel was calculated based on the supplier specifications) (**A**) and size exclusion chromatography (**B**). Enbrel (TNFR2-Fc, MW approximately 150 kDa with glycosylation (~ 25%); elution volume 9,7 min), Humira (IgG1, MW approximately 150 kDa with glycosylation (~ 2-3%); elution volume 10.5 min) and FLAG peptide (elution volume ~ 13.75 min) served as reference proteins, represented by blue (Enbrel), green (Humira) and black (Flag peptide) dashed lines. Flag peptide remaining in some cases after anti-Flag affinity purification was indicated by black arrows (FLAG). The elution volume range where possible high molecular weight aggregates would appear is also marked. **C** TNFR-stimulating activity (IL8 induction, cell death) of purified oligovalent Nb:TNFR agonists and ligand-based benchmark agonists. Shown are the averaged data, and the number of independent experiments is noted in the corresponding graph. Data for each construct were analyzed with the GraphPad Prism software using ordinary one-way ANOVA. Construct-treated groups were compared with the untreated control. *p < 0.05; **p < 0.01; # *p* < 0.0001. **D** HT1080 cells were stimulated overnight with the indicated concentrations of 3xNb:CD95-GCN4 or Fc-CD95L or remained untreated. Total cell lysates were evaluated by Western blot with respect to the indicated protein species. Results of one of two similar experiments are shown. **E** HeLa-RIP3 cells were stimulated with the indicated concentrations of Fc-CD95L and 3xNb:CD95-GCN4 for 1 h. The CD95 DISC was immunoprecipitated with protein G (Fc-CD95L) or ALFA Selector^CE^ resin (3xNb:CD95-GCN4) and co-precipitated proteins were analyzed by western blotting. **F** 3xNb:muCD95-GCN4, Fc-CD95L and *Mega*CD95L were separated by SDS-PAGE and visualized by silver staining. * the amount of *Mega*CD95L applied to the gel was calculated based on the supplier specifications. **G** Size exclusion chromatography of 3xNb:muCD95-GCN4. **H** A20J cells were sensitized with 2.5 µg/ml CHX and challenged overnight with increasing concentrations of 3xNb:muCD95-GCN4, Fc-CD95L and *Mega*CD95L and were finally analyzed for viability. For statistical evaluations averaged data of 3 (*Mega*CD95L) or 4 (3xNb:muCD95-GCN4, Fc-CD95L) independent experiments were analyzed using ordinary one-way ANOVA. Construct-treated groups were compared with the untreated control. *p < 0.05; **p < 0.01; # *p* < 0.0001.

To rule out that the agonism of the oligovalent Nb:TNFR fusion proteins is not artificially caused by protein aggregation resulting in mixtures of molecules of varying stoichiometry with potentially varying agonistic activity, we purified a subset of the oligovalent Nb constructs and analyzed them by gel filtration. Purification was either achieved by affinity chromatography on anti-Flag mAb M2 agarose and elution with Flag peptide or by affinity chromatography on ALFA Selector^CE^ or ALFA Selector^OM^ agarose and elution with the corresponding elution peptide (Fig. 5A). Purified proteins were separated by SDS-PAGE and silver staining of the gels revealed good purity of all proteins (Fig. 5A). The purified oligomeric Nb:TNFR variants furthermore eluted in gel filtration analyses largely as roughly symmetric peaks in the range of the size expected for the correctly assembled 3xNb:TNFR fusion proteins (Fig. 5B). 3xNb:TNFR fusion protein constructs of the same type and thus of similar size eluted partly with quite different volumes, e.g. the various 3xNb:GITR(C06) constructs versus the corresponding 3xNb:CD95(1G4) constructs (Fig. 5B). This seemingly surprising finding can be straight forwardly explained by the highly variable CRD3 loop of the Nb domains, which result in considerable different hydrodynamic sizes of the latter. Furthermore, there were no or only traces of aggregated high molecular weight (HMW) protein species preceding the construct peaks (Fig. 5B). Most important, the affinity chromatography purified proteins showed still the high agonistic activity observed before already with the corresponding Nb:TNFR construct-containing cell culture supernatants (Fig. 5C). Moreover, we exemplarily evaluated the activity of four 3xNb:TNFR-GCN4 constructs purified either alone by affinity chromatography or by affinity chromatography plus preparative gel filtration (Supplementary Fig. S5). From this comparison, there was no evidence for different activities of the two preparations again arguing that aggregated protein species did not affect the activity results. Furthermore, we analyzed most of the purified oligovalent Nb:TNFR agonists side by side with commercially available or in-house developed agonistic TNFL constructs of documented, well-established high activity as benchmarks. The oligovalent Nb:CD40 fusion proteins were benchmarked with *Mega*CD40L, a commercially available hexavalent fusion protein of soluble human CD40L (aa 116-261) and the collagen domain of murine ACRP30 (aa 18-111; Supplementary Fig. S6). Since soluble TNF does not properly activate TNFR2 [21], *Mega*TNF, a hexavalent fusion protein of soluble human TNF (aa 85-233) and the collagen domain of murine ACRP30, was used as a commercially available benchmark for the oligovalent Nb:TNFR2 constructs. Since the commercially available benchmark *Mega*TNF is not TNFR2-specific, we used for analysis of the oligovalent Nb:TNFR2 agonists TNFR2-expressing HeLa transfectants with CRISPR/Cas9-deleted TNFR1 [22]. *Mega*CD95L, a commercially available hexavalent fusion protein of soluble human CD95L (aa 139-281) and the collagen domain of murine ACRP30 [23], and Fc-CD95L, an in-house produced hexavalent fusion protein of soluble human CD95L with the Fc domain of human IgG1, were used to benchmark the oligovalent Nb:CD95 and Nb:mu(CD95) constructs (Supplementary Fig. S6). Furthermore, the GITR- and OX40-engaging Nb-based agonists were benchmarked with commercially available C-terminally His-tagged soluble human GITRL (aa 74-199) and human Fc-OX40L (aa 50-138). All purified oligovalent Nb agonists were as active as or even more active than the corresponding ligand-based benchmarks with respect to EC50-values and maximum IL8 or apoptosis response. Indeed, the dodecavalent Nb:CD95 construct induced half-maximal cell killing with more than one order of magnitude lower concentrations than the two benchmarks, *Mega*CD95L and Fc-CD95L. The superior activity of 3xNb:CD95-GCN4 was also evident from western blot analysis of caspase activation and immunoprecipitation of the CD95 death-inducing signaling complex (DISC) (Fig. 5D, E). The striking superiority of the dodecavalent Nb:CD95 agonist seems not to be a special feature of the specific Nb used, because we observed similar superiority of agonism also with the nonameric Nb:muCD95 agonist (Fig. 5F–H).

**Fig. 6:**
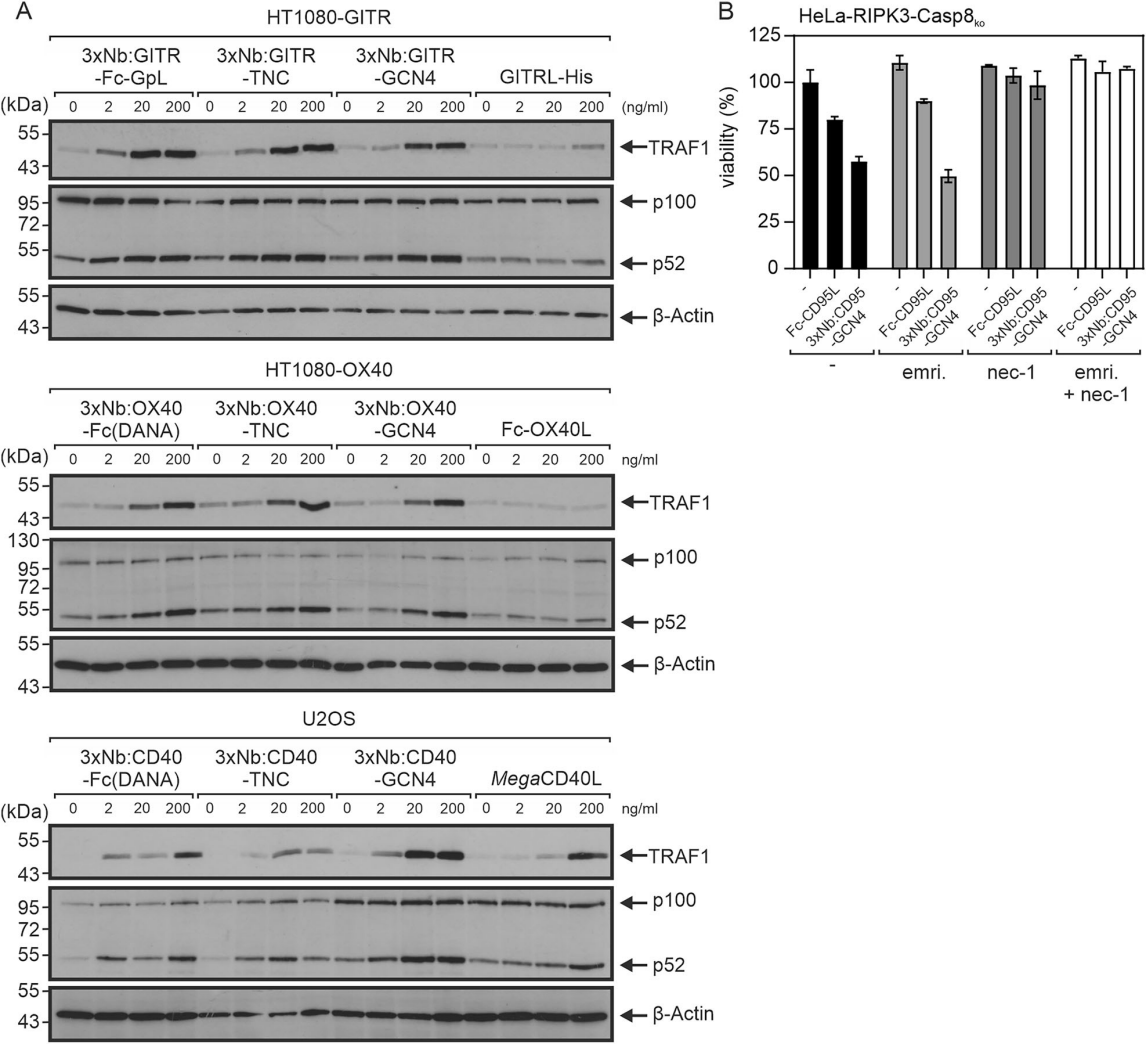
Stimulation of alternative NFκB signaling and necroptosis by 3xNb:TNFR-GCN4 constructs. **A** HT1080-GITR-, HT1080-OX40- and U2OS cells (endogenous CD40 expression) were stimulated as indicated with hexavalent (3xNb:TNFR-Fc(DANA), nonavalent (3xNb:TNFR-TNC) or dodecavalent 3xNb:TNFR-GCN4 constructs or ligand-based benchmarks for 16 hours. Total cell lysates were then analyzed for the presence of the indicated proteins by western blotting. Results of one of two similar experiments are shown. **B** HeLa-RIPK3-caspase-8-KO cells were stimulated overnight with 500 ng/ml of 3xNb:CD95 or Fc-CD95L, along with the indicated combinations of 90 µM necrostatin-1 (nec-1) and 10 µM emricasan. Cells were finally analyzed for viability by crystal violet staining. Results of one of two similar experiments with technical triplicates are shown (mean ± SD).

To this point, we focused our functional analysis on IL8 induction as a hallmark of classical NFκB pathway engagement and apoptotic cell death induction activation. However, there are several other important TNFR-associated signaling pathways, including, among others, the alternative NFκB pathway and necroptotic RIPK1 signaling. The alternative NFκB pathway signals activation of certain members of the NFκB transcription factor family by mechanisms quite distinct from those of the classical NFκB pathway, and necroptotic RIPK1 signaling results in a form of cell death, which, in contrast to apoptosis, does not involve caspase activation. Since TNFRs are molecularly linked with the alternative NFκB pathway and necroptotic RIPK1 signaling by other molecular mechanisms than the classical NFκB pathway and apoptosis signaling, we checked some of the oligovalent Nb:TNFR agonists for their ability to stimulate these pathways. To examine the activation of the alternative NFκB signaling pathway, we stimulated HT1080-TNFR transfectants and U2OS (endogenous expression of CD40) cells with the 3xNb:TNFR constructs targeting GITR, OX40 and CD40, along with the corresponding ligand-based benchmarks and analyzed by western blot processing of the p100 NFκB precursor protein to p52, a central biochemical hallmark of the alternative NFκB signaling pathway [24]. Additionally, we analyzed the induction of TRAF1, which is induced by the classical and alternative NFκB pathway [25]. The ligand-based benchmarks triggered these biochemical events to a variable, rather poor extent (Fig. 6A). The oligovalent Nb:TNFR constructs, however, triggered these events clearly and much more efficiently, starting at concentrations between 2 and 20 ng/ml, thus in the sub-nanomolar range (Fig. 6A). In order to study the necroptosis-inducing capacity of the 3xNb:CD95-GCN4 construct targeting the human CD95 death receptor, we used HeLa-RIPK3-caspase-8 knockout cells. Death receptors are unable to trigger apoptotic cell death in this cell variant due to the absence of caspase-8, but instead are able to stimulate necroptosis due to ectopic RIPK3 expression [26]. In this system, 3xNb:CD95-GCN4 was again highly agonistic and more active as the ligand-based benchmark (Fig. 6B). In accordance with the assumption that the observed cell killing was due to necroptosis induction, the caspase-8 inhibitor emricasan showed no effect in these experiments, while necrostatin-1, an inhibitor of RIPK3, which is essential for DR-induced necroptosis, completely abrogated cell death induction by 3xNb:CD95-GCN4.

**Fig. 7:**
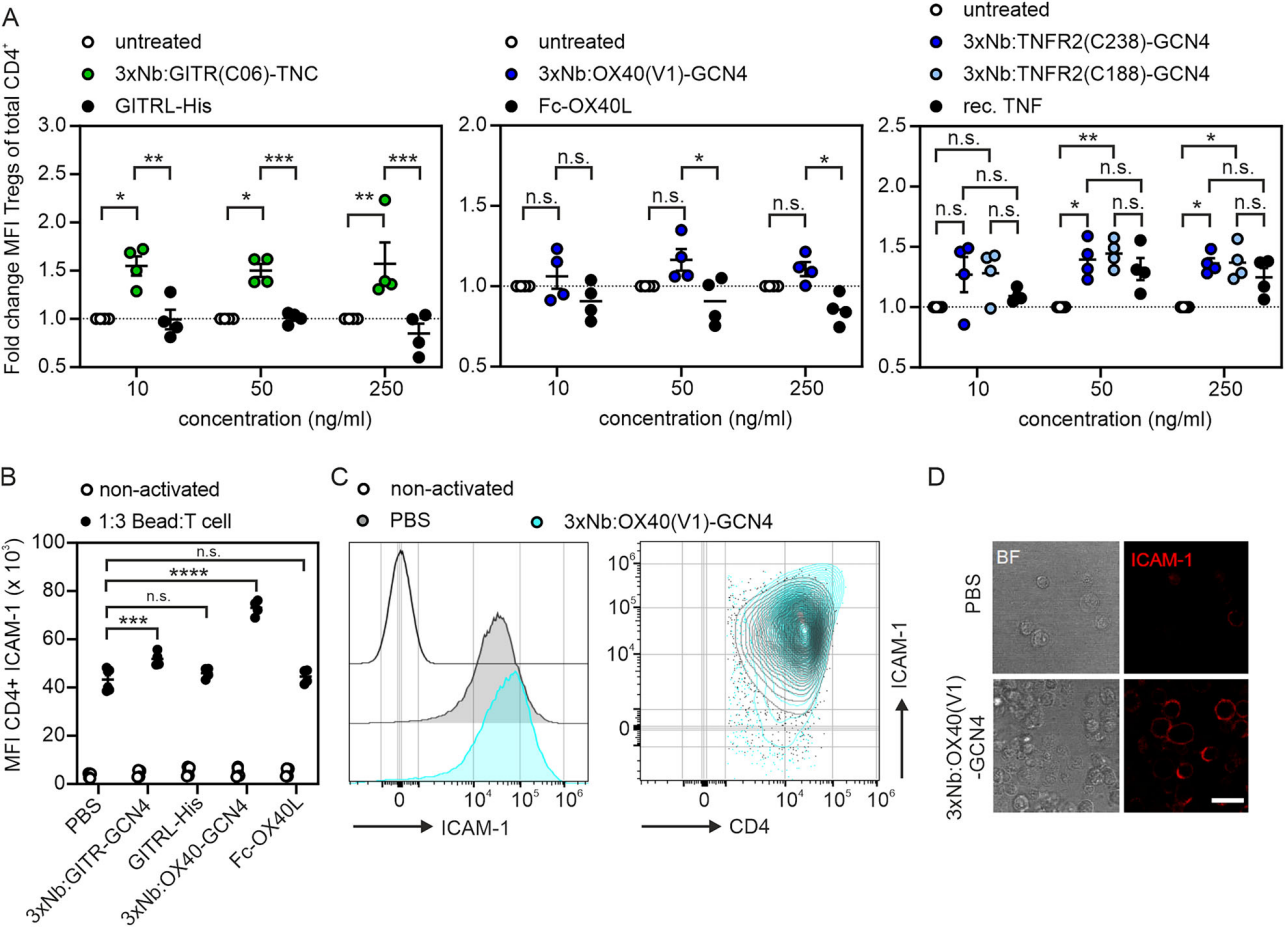
3xNb:TNFR fusion proteins stimulate human primary T cells. **A** Change of frequencies of CD3^+^CD4^+^FoxP3^+^ Tregs in human peripheral blood mononuclear cells (PBMCs) after 4 days of stimulation with the indicated TNFR agonists relative to untreated control samples of the same donor. Four donors were analyzed. **B** Activated conventional T cells (Tcons) were stimulated for one day with 200 ng/ml of the indicated OX40 and GITR agonists and were analyzed for the mean fluorescence intensity (MFI) of ICAM-1 expression by flow cytometry. Two donors were analyzed with technical duplicates. Analysis of ICAM-1 expression of 3xNb:OX40-GCN4-, Fc-OX40L- and PBS-treated Tcons by flow cytometry (**C**) and fluorescence microscopy (**D**). One representative example is shown. *p < 0.05, **p < 0.01, ***p < 0.001; ****p < 0.0001.

Finally, we evaluated the agonistic activity of a representative from each of the oligovalent 3xNb-based TNFR2, GITR and OX40 agonist panels compared to the ligand-based benchmark agonists using primary cells with endogenous TNFR expression. One important function of TNFR2 and GITR is to promote Treg proliferation [27, 28]. Thus, we tested the ability of 3xNb:GITR-TNC, 3xNb:OX40-GCN4, 3xNb:TNFR2-GCN4, along with the benchmarks GITRL-His, Fc-OX40L and TNF to increase Treg frequency in 4-day human high-density PBMC cultures. 3xNb:GITR-TNC and two different 3xNb:TNFR2-GCN4 variants significantly increased Treg frequency already with 10 ng/ml and 50 ng/ml while conventional TNF and GITRL-His showed no Treg expansion even when used with 250 ng/ml (Fig. 7A). There was also a trend towards Treg expansion with 3xNb:OX40 but not with Fc-OX40L (Fig. 7A). The poor effect of the oligovalent Nb:OX40 agonist is in good accordance with previous findings reporting superior Treg expansion via TNFR2, DR3, 41BB, GITR in comparison to OX40 [29]. We therefore analyzed 3xNb:OX40-GCN4 and its Fc-OX40L benchmark in a second setup: costimulation of conventional T cells treated with anti-CD3/anti-CD28 beads. As a marker of costimulation, we analyzed expression of ICAM-1. 3xNb:OX40-GCN4 significantly enhanced anti-CD3/anti-CD28-induced ICAM-1 expression while the less active Fc-OX40L benchmark molecule showed no major effect (Fig. 7B–D) confirming the superior performance of 3xNb:OX40-GCN4 observed before (Figs. 5C and 6A). The superior activity of the 3xNb:GITR-GCN4 molecule compared to its benchmark GITRL-His, observed in Figs. 5C and 6A, was also confirmed in T cell costimulation experiments (Fig. 7B). Thus, in sum, the results of the T cell experiments demonstrated again the much better activity of oligovalent Nb-based agonists compared to established ligand-based agonists.

## Discussion

Over three decades have passed since the identification of the 19 genes encoding ligand protomers of the TNFSF, and many concepts and strategies have been followed to generate TNFR agonists with high intrinsic activity. However, these concepts and strategies have often only be applied to few TNFR types and/or only result in potent agonists for a subset of TNFRs. The first very straightforward approach was simply to produce the extracellular THD-containing part of the TNFL molecules recombinantly as soluble proteins. In fact, this approach results in some cases in potent agonists. For example, soluble TNF efficiently stimulates TNFR1 and is even approved for clinical use in the therapy of soft sarcoma by isolated limb perfusion [30]. However, soluble TNF fails to stimulate TNFR2 and thus already exemplify that soluble TNFL molecules not necessarily act in every case as bona fide TNFR agonists [21]. Similar findings were also made with other ligands of the TNFSF, such as CD27L, OX40L, CD95L, TRAIL and several more which show as soluble ligand trimers no or only suboptimal agonism [5]. Inactive soluble TNFLs, however, can be agonistically empowered by stabilization of their trimeric organization and/or physical linkage of two or more ligand trimers. For example, the intrinsic trimerization capacity of the isolated THD of some TNFLs, like TRAIL, is rather weak which makes it necessary to additionally stabilize the trimeric assembly of the three THD protomers by genetic engineering, e.g. by connecting the protomers by short linkers (single-chain sTNFLs) or by fusion of the THD with trimeric oligomerization domains [9–11]. Strong activation of category II TNFRs by TNFL-based agonists furthermore requires constructs in which two or more THD trimers are physically linked. In the corresponding cases, this again prompts an additional need for genetic fusion with an appropriate oligomerization domain. For example, genetic fusion of sTNFL with the Fc domain of hIgG1 or the collagen-domain of ACRP30 can result in many cases in potent hexavalent TNFR agonists, and genetic fusion of sTNFLs with the surfactant protein-D (SP-D) scaffold results in dodecavalent agonists [23, 31–34]. Furthermore, genetic fusion of stabilized single-chain sTNFLs (scTNFLs) with a Fc domain, an IgG1 antibody or non-antibody oligomerization domains, such as the tenascin-C trimerization domain or the tetrameric GCN4 domain, allows the production of hexa-, nona- or dodecameric agonists with high activity [35–38]. While the practicability of ACRP30 and Fc fusion proteins has been demonstrated for several types of TNFLs, the usefulness of the scTNFL fusion proteins as agonists has mainly been demonstrated for a TNFR2-specific mutant of sTNF, sTRAIL and sCD40L [10, 36, 39]. Agonism-conferring crosslinking of sTNFL trimers can also be accomplished with antibodies. However, this results in mixtures of TNFL molecule species of varying stoichiometry, which are not promising options for clinical development [5]. Moreover, recombinant TNFL variants frequently suffer from stability issues, are prone to aggregation or have only poor half-life in vivo. This is particularly well investigated for TRAIL [40]. Taken together, there are various useful formats for potent ligand-based TNFR agonists, but there is no construct type yielding efficient agonism, good CMC properties and high serum retention for each TNFL type.

Antibodies might also be used to engage receptors of the TNFRSF. Activating receptor clustering, however, as already discussed briefly in the introduction, is again an issue that is not straightforwardly and generally achieved. Bivalent IgG antibodies are strongly favored for clinical development due to their favorable CMC properties, but typically fail to sufficiently engage receptor clustering, resembling the situation with soluble TNFL trimers. Decavalent IgM antibodies as well as IgG3 and human IgG2 isoform B antibodies which are prone for aggregation are more agonistic than bivalent IgG classes of the same idiotype [41–46] but suffer from poor productivity and heterogeneous complex formation. Moreover, in previous studies using CD27-, CD40-, CD95-, OX40- and TRAILR2-specific conventional antibodies, it was found that the intrinsic, i.e., FcγR-independent, agonism of anti-TNFR IgG antibodies, depends on the binding to specific areas of the TNFR molecule. The TNFR part of relevance for agonism seems to vary from case to case. For OX40 and CD27, for example, it was found that IgG antibodies that recognize a plasma membrane proximal epitope have particularly good agonistic effects [47–49]. In contrast, CD40-specific human IgG2 antibodies targeting the plasma membrane-distal CRD1 are agonistic but lack agonism when targeting plasma membrane-distal epitopes [44, 45] and agonism of antibodies of the death receptors CD95 and TRAILR2 seems to be tightly linked with the recognition of a patch of positively charged residues located in CRD2 and CRD3 of these receptors [50, 51]. Taken together, the development of anti-TNFR antibody agonists with strong intrinsic activity appears not less individualized than that of ligand-based TNFR agonists. Moreover, it is presumably even more complex, given that TNFR-bound antibodies also function as FcγR agonists, potentially eliciting off-target activities unrelated to the intended TNFR signaling.

The experience with ligand- and antibody-based TNFR agonists points to valency as a crucial factor conferring agonism to TNFR binding molecules. To evaluate this idea in a broad and comprehensive manner, we analyzed the intrinsic agonistic activity of bi-, hexa-, nona- and dodecavalent variants of a panel of nanobodies (Nbs) targeting 9 of the 25 signaling competent human TNFRs and three murine TNFRs (Table 1). The bivalent Nb variants were obtained by genetic fusion with the human Fc domain, while the hexa-, nona- and dodecavalent variants were generated by fusing single-chain encoded triplets of the nanobodies with oligomerizing protein scaffolds. It turned out that all nanobodies were non- or poorly agonistic in bivalent form (Fig. 2). Constructs with a valency of six or more, however, acted as potent TNFR agonists with sub-nanomolar activity (Figs. 4 and 5C). The nanobodies used in our study to construct TNFR agonists target quite different types of TNFRs, ranging from BCMA, which only possesses one single CRD in the extracellular part, over TNFR1, CD95 and TRAILR2, which have 2.5 to 4 CRDs and which represent two functionally different categories of death receptors, to CD40, 41BB, OX40, GITR and TNFR2 which have 2 to 4 CRDs and belong to a subgroup of the TNFRSF directly interacting with various members of the TRAF protein family [52]. The nanobodies used in this study furthermore cover ligand blocking and non-blocking ones (Fig. 1D). Despite the large diversity of the nanobodies used, their hexa-, nona- and dodecavalent variants regularly displayed high activity. The specific activity of hexa-, nona- and dodecavalent constructs derived from the nanobodies specific for GITR, OX40, CD40, TNFR2, TRAILR2 and muTNFR1 was largely similar, whereas the specific activity of the 41BB, BCMA and human and murine CD95 specific constructs increased further with higher valency. Thus, although valency is obviously the by far most dominant factor conferring agonism to TNFR-specific nanobody constructs, TNFR type- and/or construct type-specific factors might have to some extent a modifying secondary effect on the relevance of valency for TNFR agonism. In sum, the presented approach for the construction of TNFR agonists is broadly applicable, simple and delivers by far less variable/individualized results as the various ligand- and antibody-based approaches discussed above. Worth mentioning, the well-established excellent CMC properties (stability, production yields, lack of aggregation) of nanobodies are preserved in the oligovalent nanobody formats.

Last but not least, the finding that structurally and functionally diverse TNFRs are efficiently engaged by agonists of identical domain architectures is also interesting with respect to the understanding of the molecular mechanisms of TNFR activation. Since the nanobodies used to construct the TNFR agonists of this study were not preselected in any way for the part or epitope recognized in their TNFR target, this issue is unlikely to be of relevance for oligovalent Nb-based TNFR agonists. This in turn eventually argues against the idea that molecularly ordered and/or structurally demanding assembly processes are obligate for TNFR activation. Instead, it appears that induced proximity of six or more spatially unordered TNFR molecules is the only common but sufficient event for receptor activation by oligovalent Nb-based TNFR agonists.

## Materials and methods

### Reagents

The FcγRIA expression plasmid (pCMV-SPORT6) was obtained from SourceBioScience (Nottingham, UK), and the expression plasmid for BCMA was a kind gift of Pascal Schneider (University of Lausanne). Ligand-based TNFR „benchmarks“ were purchased from AdipoGen (CA, USA; Fc-OX40L #AG-40B-0172, *Mega*CD40L #AG-40B-0010-C010, GITRL-His #AG-40A-0024T-C010, *Mega*TNFα #AG-40B-0019-C010, *Mega*CD95L #AG-40B-0130-C010).

Following antibodies were used for Western Blotting: Direct-Blot™ HRP anti-Flag Tag antibody (BioLegend, San Diego, USA), anti-ALFA HRP (NanoTag Biotechnologies, Germany #N1505-HRP), anti-Caspase-3 (#14220), anti-Caspase-9 (Cell Signaling, MA, USA, #9503), anti-TRAF1 (Cell Signaling, MA, USA, #4715), anti-CD95 (Cell Signaling, MA, USA, #8023), anti-PARP (BD Biosciences, NJ, USA, #556494), anti-Caspase8 (Enzo Life Science, #ADI-AAM-118E), anti-β-actin (Sigma, #A1978-200UL), anti-p100/p52 (Merck Millipore, #05-361), anti-FLIP (AdipoGen, #AG-20B-0056-C100) and anti-FADD (Cell Signaling, MA, USA, #2782). Carbobenzoxy-valyl-alanyl-aspartyl-[Omethyl]-fluoromethylketone (ZVAD) was from Bachem (CA, USA), necrostatin-1 (nec-1) from MedChemExpress (NJ, USA; #HY-15760), cycloheximide (CHX) from Sigma and emricasan also from MedChemExpress (NJ, USA; #HY-10396).

### Cell culture and cell lines

All cell lines used were cultured at 37 °C and 5% CO_2_. Human cell lines HEK293T, HT1080, HT1080 stably transfected with TNFRs, HCT116, HeLa-TNFR2-TNFR1_ko_, HeLa-RIPK3 (kind gift of Markus Leverkus, University Hospital Aachen, ref. [53]), as well as the caspase-8-deficient variant derived thereof, HeLa-RIPK3-casp8_ko_ [26] were maintained in RPMI1640 medium (Sigma-Aldrich, Steinheim, Germany) supplemented with 10% fetal bovine serum (FBS; GIBCO). Murine cell lines PanCO2 and A20J and human U2OS cells were cultured using DMEM supplemented with 10% FBS (Sigma-Aldrich, Steinheim, Germany). The stable transfectants were regularly controlled by flow cytometry for the presence of the transfected TNFR. The TNFR signaling capabilities of the transfectants were regularly analyzed and compared with “historic” benchmarks, and by analyzing IL8 induction with recombinant TNF.

### Nanobody generation and identification

To generate single-domain antibodies (sdAbs) targeting the ectodomains of human and murine CD95, two male alpacas were immunized six times with 60 µg of recombinant proteins containing the respective target domain fused to a human IgG1 Fc domain. The antigens were administered with Adjuvant F (GERBU #3030) according to the manufacturer’s recommendations. Five days after the final immunization, total RNA was extracted from peripheral blood mononuclear cells (PBMCs) isolated from 150 mL of blood. The cDNA encoding sdAbs was amplified using a three-step nested RT-PCR protocol and subsequently cloned into a pHEN2-derived phagemid vector. Recombinant phages displaying target-specific sdAbs were enriched in three successive rounds of phage display while continuously counter-selecting against human IgG1 Fc. For each target protein, a total of 96 individual clones from the enriched pool of sdAbs were recombinantly expressed in *Escherichia coli* and analyzed by enzyme-linked immunosorbent assay (ELISA) for specific binding to the immunized target and an unrelated control protein harboring a human IgG1 Fc domain.

### Binding studies

To evaluate the binding properties of the different Nb:TNFR-Fc-GpL constructs, we performed cell-free or cellular binding studies. For cell-free binding studies, the Fc-tagged extracellular domain of the TNFR (Fc-TNFRed) of interest (1 µg/ml in 0.1 M carbonate buffer) was coated overnight at 4 °C to black high-binding 96-well plates along with an irrelevant IgG1 antibody as a negative control. The next day, wells were washed three times with PBS-Tween (PBST) and the remaining protein binding capacity was blocked with 10% FBS in PBS for 1 h. After an additional three washing steps, Nb:TNFR-Fc-GpL fusion proteins were added pairwise in increasing concentrations to the Fc-TNFRed and negative control antibody-coated wells. Following 1 h incubation at 37 °C, plates were finally washed 5-10 times in ice-cold PBS to remove unbound proteins. For measurement of well-remaining GpL activity, 50 µl RPMI 1640 medium, supplemented with 0,5% FBS and 1% Penicillin/Streptomycin, was added to each well and, after adding 25 µl substrate solution (1.5 µM coelenterazin (Carl Roth, Karlsruhe, Germany) in PBS), luminescence signals were immediately quantified using a LUmo luminometer (anthos Mikrosysteme GmbH, Friesoythe, Germany). Specific binding values were calculated by subtracting the unspecific binding values (control antibody-coated wells) from the corresponding total binding values (Fc-TNFRed-coated wells). K_D_ (dissociation constant) values were obtained by fitting to „one site specific binding“ interaction using the non-linear regression analysis function of the GraphPad Prism 10 software. In the case of cellular binding studies, TNFR-expressing cells and corresponding control cells w/o TNFR expression were seeded in black 96-well cell culture plates (2 ×10^5^ per well) and cultivated overnight. The next day, cells were treated pairwise with increasing concentrations of corresponding Nb:TNFR-Fc-GpL fusion proteins for 1 h at 37 °C. Plates were subsequently washed 5-10 times with ice-cold PBS to remove unbound GpL fusion proteins and otherwise handled as described above for the cell-free binding studies.

We also performed binding studies to evaluate if the Nb:TNFR-Fc-GpL fusion proteins interfere with ligand binding. Therefore, TNFR-positive and corresponding control cells w/o TNFR expression (2 × 10^5^ per well) were seeded in black 96-well cell culture plates, or alternatively, Fc-TNFRed and control antibody (1µg/ml) were coated to black 96-well high-binding plates. The next day, TNFR-positive wells and “control” wells were incubated with 5 µg/ml of GpL-free oligovalent Nb:TNFR fusion proteins in triplicate for 30 min at 37 °C or remained untreated. Then, 20 ng/ml of GpL-TNFL fusion proteins [54] were added to all wells for an additional hour. Finally, cell-associated GpL activity was measured as described above.

### Cloning, expression and purification of recombinant proteins

To obtain expression plasmids for the various Nb:TNFR fusion proteins, synthetic and PCR-generated DNA fragments encoding the protein domains of interest were cloned into the pCR3 expression vector using standard cloning techniques. Amino acid sequences of the Nb:TNFR fusion proteins used are listed in Supplementary Table S3. All recombinant proteins used were produced by transient transfection of HEK293T cells with corresponding expression plasmids using polyethylenimine (PEI, Polyscience Inc., Warrington, USA) as described in [55]. One day post-transfection, PEI/DNA-containing transfection medium was replaced by RPMI1640 medium supplemented with 2% FBS and 1% Pen/Strep. After 5–7 days, supernatants were harvested and cleared from cell debris by centrifugation (10 min, 4360 g). Protein concentrations were estimated by western blotting using either anti-Flag-HRP antibody or HRP-conjugated sdAb anti-ALFA (NanoTag Biotechnologies #N1505-HRP) and comparison with a Flag- and ALFA-tagged protein of known concentration on the same blot.

Flag-tagged nanobody fusion proteins were purified by anti-Flag affinity chromatography using Flag peptide as eluent according to the manufacturer's protocol (Sigma-Aldrich, Steinheim, Germany). ALFA-tagged sdAb fusion proteins were purified either on ALFA Selector^CE^ (NanoTag Biotechnologies #N1512) or ALFA Selector^OM^ resin (NanoTag Biotechnologies #N1513) and eluted using the corresponding elution peptides (ALFA elution peptide, NanoTag Biotechnologies #N1520 or ALFA elution peptide^OM^, NanoTag Biotechnologies #N1521, respectively). Purity and concentration of purified proteins were controlled by SDS-PAGE and silver staining using the Pierce Silver Stain Kit (Thermo Fisher Scientific, MA, USA) along with proteins of known size and concentration (Low Molecular Weight Calibration Kit for SDS Electrophoresis, GE Healthcare, UK Limited, Little Chalfont, UK).

### High-performance liquid chromatography (HPLC)

Affinity-purified protein samples (100-500 µl, 50-1000 µg/ml) were analyzed by gel filtration on a MabPac SEC-1 column (Thermo Fisher Scientific, MA, USA) in PBS with a flow rate of 0.76 ml/min using the UltiMate 3000 HPLC system (Thermo Fisher).

### Flow cytometry

To verify TNFR expression, cells were harvested, washed and resuspended in PBS (0.5–2 ×10^6^ cells). After incubation for one hour on ice with fluorophore-labeled antibodies along with corresponding fluorophore-labeled isotype control antibodies, cells were washed three times with PBS to remove unbound antibodies. The antibody concentrations were used according to the manufacturer’s instructions. Finally, cells were analyzed with the FACSCelesta™ (BD, Pharmingen).

### IL8 and muMCP-1 ELISA

In brief, cells of interest were seeded overnight in 96-well cell culture plates (2 × 10^5^ cells per well). The next day, the medium was replaced by fresh medium supplemented with a dilution series of proteins of interest. After an additional day, cell culture supernatants were analyzed for IL8 or murine MCP-1 expression using the BD OptEIA™ human IL8-ELISA kit and the BD OptEIA™ Murine MCP-1-ELISA set (BD Biosciences, NJ, USA).

### Viability assay

Adherent cells were cultivated overnight in 96-well cell culture plates (2 × 10^5^ cells per well), and suspension cells (A20J) were seeded directly before treatment (8 × 10^5^ cells per well). Cells were stimulated with increasing concentrations of the proteins of interest. Where indicated, cells were pretreated for 30 minutes with cycloheximide (CHX, 2.5 µg/ml) prior to stimulation to sensitize for cell death induction. The next day, the viability of adherent cells was quantified by crystal violet staining, and the viability of suspension cells was analyzed using the MTT staining assay.

### Immunoblotting

Cells of interest were seeded overnight in 6- or 12-well cell culture plates (1 ×10^6^/0.5 × 10^6^ cells per plate). Then, cells were stimulated as indicated and afterwards scraped into medium using a rubber policeman. After transfer to 1.5 ml tubes, cells were pelleted by centrifugation and washed once with PBS. Cell pellets were then resuspended in 4x Laemmli buffer supplemented with protease (Roche, Germany) and phosphatase inhibitor (Sigma-Aldrich, Weinheim, Germany) cocktails. Next, samples were sonicated (25 s, maximum amplitude, UP100H Ultrasonic Processor, Hielscher, Germany) and boiled for 5 min at 95 °C. Lysates were subjected to separation by SDS-Page after an additional centrifugation. After transfer of proteins to a nitrocellulose membrane and blocking with 5% (w/v) non-fat dry milk, membranes were incubated with antibodies as indicated. Finally, proteins were made visible by chemiluminescence western blot detection using ECL (Amersham Bioscience).

### Immunoprecipitation

The CD95 signaling complex was immunoprecipitated using either Fc-Flag-CD95L and protein G beads (Roche) or 3xNb:CD95(1G4)-ALFA-GCN4 and ALFA Selector^ST^ affinity resin (NanoTag Biotechnologies, Germany #N1511). Cells were seeded to 80% confluency in 15 cm tissue culture plates and stimulated with 1, 10, 100 or 1000 ng/ml Fc-Flag-CD95L or 3xNb:CD95(1G4)-ALFA-GCN4 for 1 h. Untreated cells served as a negative control. Cells were washed three times with ice-cold PBS to remove unbound proteins. After adding 10 ml PBS, cells were harvested using a rubber policeman and pelleted. Cell pellets were resuspended in 1.5 ml lysis buffer (30 mM Tris HCl, pH 7.5, 120 mM NaCl, 1% Triton X-100, 10% glycerol) supplemented with protease inhibitor (cOmplete™ protease inhibitor cocktail, Sigma) and incubated 20 min on ice. Then, two rounds of centrifugation (5000 × *g*, 5 min and 14,000 × *g*, 30 min) were performed to remove insoluble cell debris. To untreated control lysates 1 ng Fc-Flag-CD95L or 1 ng 3xNb:CD95(1G4)-ALFA-GCN4 were added. Lysates of Fc-CD95L-treated cells were supplemented with 30 µl protein G agarose, while lysates of cells treated with 3xNb:CD95(1G4)-ALFA-GCN4 were supplemented with 30 µl ALFA Selector^ST^ affinity resin. After incubation at 4 °C overnight with gentle agitation, beads were collected by centrifugation for 30 s at 500 × *g* and washed in lysis buffer four times. Next, beads were resuspended in 140 µl 4× Laemmli buffer and heated for 15 min at 90 °C. Beads were again pelleted by centrifugation. Finally, cleared supernatants were subjected to immunoblotting.

### Isolation, stimulation and analysis of T cells

Peripheral blood mononuclear cells (PBMCs) were isolated from leukocyte reduction system (LRS) chambers obtained from the Institute of Clinical Transfusion Medicine and Hemotherapy of the University Hospital Würzburg. The sex of the donors was not available to us. Briefly, 7 mL of blood from one LRS chamber was mixed with 13 ml PBS and equally distributed into two SepMate™ 50 ml tubes (STEMCELL Technologies, #85450) preloaded with 15 mL Lymphoprep™ (STEMCELL Technologies, #07811). After centrifugation at 1200 × *g* for 10 min at room temperature, the PBMC layer was collected, transferred to a fresh tube and washed twice with PBS (300 × *g*, 5 min, RT). The PBMC pellet was resuspended in complete RPMI medium (cRPMI) supplemented with 10% fetal calf serum (FCS), 1% L-glutamine and 1% penicillin/streptomycin and cultured at high-density (20 ml at 1 × 10⁷ cells/ml in a standing T75 tissue culture flask) for two days.

Single-cell suspensions of enriched total T cells were isolated from the high-density cultures by negative selection using the Untouched™ Human T Cell Isolation Kit (Invitrogen, #11344D) according to the manufacturer’s instructions. After removal of the bead-bound cells using a magnetic rack, the supernatant containing untouched T cells was collected and re-cleared to remove residual beads. T cells were washed, resuspended in human T cell culture medium, and counted. T cell purity was assessed by flow cytometry using a viability dye and antibodies against CD3, CD4, and CD8. T cells were activated for 4 days using CD3/CD28 Human T-Activator Dynabeads (Invitrogen, #11131D) at a bead-to-cell ratio of 1:3 according to the manufacturer’s instructions. Activated T cells (1 × 10⁵ cells per well) were stimulated in 96-well plates for 24 h in cRPMI supplemented with 10% FCS, 1% L-glutamine and 1% penicillin/streptomycin with 200 ng/ml of the indicated agonists or PBS as a control.

For stimulation of regulatory T-cells, PBMCs cultured at high density for 2 days were plated at 5 × 10⁵ cells per well in 96-well plates and were cultured for an additional 4 days in cRPMI containing 10% FCS, 1% L-glutamine and 1% penicillin/streptomycin in the presence of the agonists of interest or PBS as a control.

For flow cytometric analyses, cell cultures and single-cell suspensions were incubated in washing buffer (5% FCS in PBS) for 10 min at RT prior to staining. Cells were stained with fluorochrome-conjugated monoclonal antibodies for 30 min at 4 °C. The following antibodies were used: anti-CD3 (OKT3, BioLegend), anti-CD4 (OKT4, BioLegend), anti-CD8 (SK1, BioLegend), anti-CD25 (BC96, BioLegend), and anti-CD54 (HCD54, BioLegend). To allow exclusion of dead cells, samples were co-stained with Zombie Aqua™ Fixable Viability Dye. For intracellular Foxp3 staining, cells were fixed and permeabilized using the Foxp3/Transcription Factor Fixation/Permeabilization Kit (eBioscience, #00-5523-00) according to the manufacturer’s protocol. Anti-human Foxp3 antibody (PCH101, Invitrogen) was added to the permeabilization buffer (1× in deionized water) and incubated for 30 min at 4 °C prior to data acquisition. Data were acquired on an Attune™ NxT Flow Cytometer (Thermo Fisher Scientific). Compensation was performed using single-stained OneComp eBeads™ (Thermo Fisher Scientific, #01-1111-41) to generate compensation matrices for each antibody panel. Data analysis was performed using FlowJo v10 software.

### Statistical analysis

Data normality was tested using the Shapiro-Wilk test, and the homogeneity of variances assumption for two or more groups was validated using Levene’s test without exclusion of outliers. Statistical analyses were performed using the GraphPad Prism version 11 software. Comparison of dose-response relations between two matched conditions was analyzed using the multiple unpaired t-test. One-way analysis of variance (ANOVA) was used for multiple group comparisons. In all analyses, statistical significance is shown as *p*-values with < 0.05 considered significant. *p < 0.05, **p < 0.01, ***p < 0.001, *p*-values > 0.05 were not labeled. Further details of experimental group numbers and statistical tests are given in the corresponding figure legends.

## Supplementary information

Supplemental material 1 - data

Supplemental material 2 - original data

## Data Availability

The original full-size Western blots are shown in the Original Data WB file. Further information and requests for reagents should be directed and fulfilled by the corresponding author, H. Wajant.
