# Supplementary material for: A simple and broadly applicable nanobody-based approach to generate potent TNFR agonists
Source: Cell Death Dis. 2026 May 30;17(1):518. doi: 10.1038/s41419-026-08911-x (PMC13222343; doi:10.1038/s41419-026-08911-x)
Supplement: Supplementary file 1 — Supplemental material 1 - data [file 41419_2026_8911_MOESM1_ESM.pdf]

Supplemental Data

A simple and broadly applicable nanobody-based approach to generate potent TNFR agonists

Isabell Lang[1], Olena Zaitseva[1], Amelie Glöckler[1], Daniela Siegmund[1], Dalia Sheta[2], Bayan Mouhandes[1], Daniela Weisenberger[1], Viktoria Schäfer[1], Svetlana Stepanzow[1], Theresa Schneider[2], Andreas Beilhack[2], Alexander Crauel[3], Markus Kilisch[3], Lisa-Marie Funk[3], Hansjörg Götzke[3], Steffen Frey[3] and Harald Wajant[1]

[1] Division of Molecular Internal Medicine, Department of Internal Medicine II, University Hospital Würzburg, Würzburg, Germany.

[2] Department of Internal Medicine II, Interdisciplinary Center for Clinical Research (IZKF) laboratory Würzburg, Center for Experimental Molecular Medicine, University Hospital Würzburg, Würzburg, Germany.

[3] NanoTag Biotechnologies GmbH, Rudolf-Wissell-Straße 28a, 37079 Göttingen, Germany.

Supplemental Table S1. Concentration of oligovalent 3xNb:TNFR and TNFL variants in the supernatant of transiently transfected HEK293 cells

Protein	Productivity (µg/ml)				
	Transfection no.			Mean	SEM
	1	2	3		
3xNb:41BB-Fc(DANA)	8	13	13	11	± 1.7
3xNb:41BB-TNC	12	9	18	13	± 2.6
3xNb:41BB-GCN4	19	18	26	21	± 2.5
Soluble 41BB-L	16	13	18	16	± 1.5
Fc-41BB-L	14	16	14	15	± 0.7
3xNb:BCMA-Fc(DANA)	5	5	4	5	± 0.3
3xNb:BCMA-TNC	15	19	22	19	± 2.0
3xNb:BCMA-GCN4	16	23	19	19	± 2.0
Soluble APRIL	19	14	13	15	± 1.9
Fc-APRIL	15	13	17	15	± 1.2
3xNb:CD40-Fc(DANA)	14	10	13	12	± 1.2
3xNb:CD40-TNC	4	2	7	4	± 1.5
3xNb:CD40-GCN4	8	6	8	6	± 0.6
3xNb:CD40-GCN4	7	6	5	7	± 0.7
Fc-CD40L	21	19	15	18	± 1.8
3xNb:CD40(mu)-Fc(DANA)	5	6	6	6	± 0.3
3xNb:CD40(mu)-TNC	18	16	19	18	± 0.9
3xNb:CD40(mu)-GCN4	11	10	12	11	± 0.6
Soluble muCD40L	18	11	19	16	± 2.5
3xNb:CD95-Fc(DANA)	26	25	23	25	± 0.9
3xNb:CD95-TNC	41	38	46	41	± 2.3
3xNb:CD95GCN4	22	26	19	22	± 2.0
3xNb:CD95-GCN4	13	13	10	12	± 1.0
Soluble-CD95L	35	30	0	22	± 11
Fc-CD95L	19	20	17	19	± 0.9
3xNb:CD95(mu)-Fc-GpL	15	17	15	16	± 0.7
3xNb:CD95(mu)TNC	11	12	9	11	± 0.9
3xNb:CD95(mu)-GCN4	9	10	8	9	± 0.6
Soluble muCD95L	25	27	11	21	± 5.0
3xNb:GITR-Fc-GpL	43	29	34	35	± 4.1
3xNb:GITR-TNC	12	12	13	12	± 0.3
3xNb:GITR-GCN4	1	2	3	2	± 0.6
Fc-GITRL	10	11	11	11	± 0.3
3xNb:OX40-Fc(DANA)	10	10	13	11	± 1.0
3xNb:OX40-TNC	8	8	12	9	± 1.3
3xNb:OX40-GCN4	7	7	9	8	± 0.7
Fc-OX40L	11	9	10	10	± 0.6
3xNb:TNFR1-Fc-GpL	16	13	14	14	± 0.9
3xNb:TNFR1-TNC	5	5	8	6	± 1.0
3xNb:TNFR1-GCN4	0	0	0	0	± 0
3xNb:TNFR2-Fc(DANA)	4	4	6	5	± 0.7
3xNb:TNFR2-TNC	12	10	13	12	± 0.9
3xNb:TNFR2-GCN4	8	11	12	10	± 1.2
Soluble TNF	31	24	26	27	± 2.0

Fc-TNF	43	39	40	41	± 1.2
3xNb:TRAILR2-Fc(DANA)	2	3	1	2	± 0.6
3xNb:TRAILR2-TNC	5	4	4	4	± 0.3
3xNb:TRAILR2-GCN4	2	2	1	2	± 0.3
Fc-TRAIL	8	8	8	8	± 0

Supplemental Table S2. Characteristics of TNFR targeting molecules used in this study.

Protein	MW (kDa)[a]	Proto-mers	Va-lency	EC50 (ng/ml)		$EC50_{benchmark}$ / $EC50_{protein}$[b]
				SN	purified	
3xNb:41BB-Fc(DANA)	72	2	6	> 1000	-	-
3xNb:41BB-TNC	50	3	9	> 1000	-	-
3xNb:41BB-GCN4	51	4	12	> 1000	-	-
Nb:41BB-Fc-GpL	59	2	2	> 1000	-	-
3xNb:BCMA-Fc(DANA)	69	2	6	370	-	-
3xNb:BCMA-TNC	47	3	9	240	-	-
3xNb:BCMA-GCN4	49	4	12	47	-	-
Nb:BCMA-Fc-GpL	58	2	2	> 1000	-	-
3xNb:CD40(V12t)-Fc(DANA)	68	2	6	5/5[c]	0.7/-	16/-[c]
3xNb:CD40(V12t)-TNC	46	3	9	8/10[c]	3/-	4/-[c]
3xNb:CD40(V12t)-GCN4	46	4	12	-	0.1	110
3xNb:CD40(V12t)-GCN4	47	4	12	2.5/0.2[c]	-	4/55[c]
Nb:CD40(V12t)-Fc-GpL	57	2	2	> 1000	-	<< 1
MegaCD40L	35*	6	6	-	11	1
3xNb:CD40(1B6)-Fc(DANA)	72	2	6	31	-	0.4
3xNb:CD40(1B6)-TNC	50	3	9	15	-	0.7
3xNb:CD40(1B6)-GCN4	51	4	12	8	-	1.4
Nb:CD40(1B6)-Fc-GpL	59	2	2	> 1000	-	< 0.02
3xNb:CD40(mu)-Fc(DANA)	72	2	6	2	-	-
3xNb:CD40(mu)-TNC	50	3	9	3.5	-	-
3xNb:CD40(mu)-GCN4	52	4	12	8	-	-
Nb:CD40(mu)-Fc-GpL	59	2	2	> 1000	-	-
3xNb:CD95-Fc(DANA)	76	2	6	3	1	0.2
3xNb:CD95-TNC	54	3	9	0.3	0.04	5
3xNb:CD95-GCN4 ALFA-tag	55	4	12	0.002	-	100
3xNb:CD95-GCN4 Flag tag	54	4	12	-	< 0.001	> 200
Nb:CD95-Fc-GpL	60	2	2	> 1000	-	< 0.001
MegaCD95L	40*	6	6	-	0.2	1
Fc-CD95L	44	6	6	-	0.2	1
3xNb:CD95(mu)-Fc-GpL	90	2	6	1	-	0.2
3xNb:CD95(mu)-TNC	50	3	9	1	-	0.2
3xNb:CD95(mu)-GCN4	51	4	12	< 0.001	< 0.001	> 20
Nb:CD95(mu)-Fc-GpL	59	2	2	> 1000	-	< 0.001
3xNb:GITR-Fc-GpL	87	2	6	12	3	> 200
3xNb:GITR-TNC	46	3	9	26	5	133
3xNb:GITR-GCN4	48	4	12	7	35	19
Nb:GITR-Fc-GpL	58	2	2	> 1000	-	< 1
GITRL-His	17*	3	3	-	670	1
3xNb:OX40-Fc(DANA)	68	2	6	10	15	67
3xNb:OX40-TNC	46	3	9	15	12	85
3xNb:OX40-GCN4	48	4	12	27	37	27
Nb:OX40-Fc-GpL	58	2	2	> 1000	-	< 1
OX40L-Fc	50*	6	6	-	> 1000	1
3xNb:TNFR1-Fc-GpL	88	2	6	51	-	-
3xNb:TNFR1-TNC	48	3	9	640	-	-

3xNb:TNFR1-GCN4	50	4	12	86	-	-
Nb:TNFR1-Fc-GpL	58	2	2	> 1000	-	-
3xNb:TNFR1(mu)-Fc-GpL	89	2	6	4	-	-
3xNb:TNFR1(mu)-TNC	48	3	9	5	-	-
3xNb:TNFR1(mu)-GCN4	50	4	12	11	-	-
Nb:TNFR1(mu)-Fc-GpL	59	2	2	> 1000	-	-
3xNb:TRAILR2-Fc(DANA)	71	2	6	0.003	-	-
3xNb:TRAILR2-TNC	49	3	9	5	-	-
3xNb:TRAILR2-GCN4	50	4	12	0.8	-	-
Nb:TRAILR2-Fc-GpL	59	2	2	> 1000	-	-
3xNb:TNFR2(C188)-Fc(DANA)	72	2	6	0.4	8	10
3xNb:TNFR2(C188)-TNC	50	3	9	0.6	1	76
3xNb:TNFR2(C188)-GCN4	49	4	12	10	0.3	253
Nb:TNFR2(C188)-Fc-GpL	59	2	2	> 1000	-	< 1
MegaTNF	34*	6	6	-	76	1
3xNb:TNFR2(C238)-Fc(DANA)	71	2	6	0.5	-	152
3xNb:TNFR2(C238)-TNC	49	3	9	3	-	25
3xNb:TNFR2(C238)-GCN4	51	4	12	4	-	19
Nb:TNFR2(C238)-Fc-GpL	59	2	2	> 1000	-	< 0.1

[a] Calculated based on aa sequence or in the case of the commercial benchmarks (*) according to the supplier's information.
[b] EC50 of purified protein or, if not available, of protein SN
[c] first value based on analysis of HT1080-CD40 transfectants, second value is derived from U2OS with endogenous expression of CD40

Supplemental Table S3. pCR3-based expression plasmids and corresponding amino acid sequences.
Leader: underlined; Flag or ALFA-tag: underlined + grey background; restriction site encoding 2AA linker: bold; linker: bold + italic; Nb domains: italic; TNC trimerization domain: italic + underlined + grey background; GCN4 tag: italic + underlined + grey background + bold; Fc part: grey background.

3xNb:41BB-Fc(DANA)-pCR3	MNFGFSLIFLVLVLKGVQCEVKLVPRGTQVQLVESGGGVVQPGRSLRLSCAASGSTFSIVAM<br>GWYRQAPGKQRELVASIITGDGDTNYADSVKGRFTISRDNSKNTMYLQMNSLKPEDTAVYYC<br>YARTGYGSSWLMGHEYDYWGQGTQVTVSSLGRSGGGGSGGGGSGGGGSGGGGSGGGGSQVQL<br>VESGGGVVQPGRSLRLSCAASGSTFSIVAMGWYRQAPGKQRELVASIITGDGDTNYADSVKG<br>RFTISRDNSKNTMYLQMNSLKPEDTAVYYCYARTGYGSSWLMGHEYDYWGQGTQVTVSSLGG<br>GGGSGGGGSGGGGSGGGGSGGGGSQLQVQLVESGGGVVQPGRSLRLSCAASGSTFSIVAMGW<br>YRQAPGKQRELVASIITGDGDTNYADSVKGRFTISRDNSKNTMYLQMNSLKPEDTAVYYCYA<br>RTGYGSSWLMGHEYDYWGQGTQVTVSSLGGSKTHTCPPCPAPELLGGPSVFLFPPKPKDTLM<br>ISRTPEVTCVVVAVSHEDPEVKFNWYVDGVEVHNAKTKPREEQYASTYRVVSVLTVLHQDWL<br>NGKEYKCKVSNKALPAPIEKTISKAKGQPREPQVYTLPPSRDELTKNQVSLTCLVKGFYPSD<br>IAVEWESNGQPENNYKTTPPVLDSDGSFFLYSKLTVDKSRWQQGNVFSCSVMHEALHNHYTQ<br>KSLSLSPGKEFDYKDDDDKLE
3xNb:41BB-TNC-pCR3	MNFGFSLIFLVLVLKGVQCEVKLVPRGTQVQLVESGGGVVQPGRSLRLSCAASGSTFSIVAM<br>GWYRQAPGKQRELVASIITGDGDTNYADSVKGRFTISRDNSKNTMYLQMNSLKPEDTAVYYC<br>YARTGYGSSWLMGHEYDYWGQGTQVTVSSLGRSGGGGSGGGGSGGGGSGGGGSGGGGSQVQL<br>VESGGGVVQPGRSLRLSCAASGSTFSIVAMGWYRQAPGKQRELVASIITGDGDTNYADSVKG<br>RFTISRDNSKNTMYLQMNSLKPEDTAVYYCYARTGYGSSWLMGHEYDYWGQGTQVTVSSLGG<br>GGGSGGGGSGGGGSGGGGSGGGGSQLQVQLVESGGGVVQPGRSLRLSCAASGSTFSIVAMGW<br>YRQAPGKQRELVASIITGDGDTNYADSVKGRFTISRDNSKNTMYLQMNSLKPEDTAVYYCYA<br>RTGYGSSWLMGHEYDYWGQGTQVTVSSLGGSDYKDDDDKDIACGCAAAPDIKDLLSRLEELE<br>GLVSSLREQGTG
3xNb:41BB-GCN4-pCR3	MNFGFSLIFLVLVLKGVQCEVKLVPRGSDYKDDDDKQLRLKQIEDKLEEILSKLYHIENELA<br>RIKKLLGEREFGLEQVQLVESGGGVVQPGRSLRLSCAASGSTFSIVAMGWYRQAPGKQRELV<br>ASIITGDGDTNYADSVKGRFTISRDNSKNTMYLQMNSLKPEDTAVYYCYARTGYGSSWLMGH<br>EYDYWGQGTQVTVSSLGRSGGGGSGGGGSGGGGSGGGGSGGGGSQVQLVESGGGVVQPGRSL<br>RLSCAASGSTFSIVAMGWYRQAPGKQRELVASIITGDGDTNYADSVKGRFTISRDNSKNTMY<br>LQMNSLKPEDTAVYYCYARTGYGSSWLMGHEYDYWGQGTQVTVSSLGGGGGSGGGGSGGGGS<br>GGGGSGGGGSQLQVQLVESGGGVVQPGRSLRLSCAASGSTFSIVAMGWYRQAPGKQRELVAS<br>IITGDGDTNYADSVKGRFTISRDNSKNTMYLQMNSLKPEDTAVYYCYARTGYGSSWLMGHEY<br>DYWGQGTQVTVSSLG
Nb:41BB-Fc-GpL-pCR3	MNFGFSLIFLVLVLKGVQCEVKLVPRQLQVQLVESGGGVVQPGRSLRLSCAASGSTFSIVAM<br>GWYRQAPGKQRELVASIITGDGDTNYADSVKGRFTISRDNSKNTMYLQMNSLKPEDTAVYYC<br>YARTGYGSSWLMGHEYDYWGQGTQVTVSSLGGSKTHTCPPCPAPELLGGPSVFLFPPKPKDT<br>LMISRTPEVTCVVVDVSHEDPEVKFNWYVDGVEVHNAKTKPREEQYNSTYRVVSVLTVLHQD<br>WLNGKEYKCKVSNKALPAPIEKTISKAKGQPREPQVYTLPPSRDELTKNQVSLTCLVKGFYP<br>SDIAVEWESNGQPENNYKTTPPVLDSDGSFFLYSKLTVDKSRWQQGNVFSCSVMHEALHNHY<br>TQKSLSLSPGKEFDYKDDDDKLEKPTENNEDFNIVAVASNFATTDLDADRGKLPGKKLPLEV<br>LKEMEANARKAGCTRGCLICLSHIKCTPKMKKFIPGRCHTYEGDKESAQGGIGEAIVDIPEI<br>PGFKDLEPMEQFIAQVDLCVDCTTGCLKGLANVQCSDLLKKWLPQRCATFASKIQGQVDKIK<br>GAGGD
3xNb:BCMA(269)-Fc(DANA)-pCR3	MNFGFSLIFLVLVLKGVQCEVKLVPRGTAVQLVESGGGLVQAGDSLRLTCTASGRAFSTYFM<br>AWFRQAPGKEREFVAGIAWSGGSTAYADSVKGRFTISRDNAKNTVYLQMNSLKSEDTAVYYC<br>ASRGIEVEEFGAWGQGTQVTVSSRSGGGGSGGGGSGGGGSGGGGSGGGGSAVQLVESGGGLV<br>QAGDSLRLTCTASGRAFSTYFMAWFRQAPGKEREFVAGIAWSGGSTAYADSVKGRFTISRDN<br>AKNTVYLQMNSLKSEDTAVYYCASRGIEVEEFGAWGQGTQVTVSSGGGGSGGGGSGGGGSGG<br>GGSGGGGSQLAVQLVESGGGLVQAGDSLRLTCTASGRAFSTYFMAWFRQAPGKEREFVAGIA<br>WSGGSTAYADSVKGRFTISRDNAKNTVYLQMNSLKSEDTAVYYCASRGIEVEEFGAWGQGTQ<br>VTVSSGSKTHTCPPCPAPELLGGPSVFLFPPKPKDTLMISRTPEVTCVVVAVSHEDPEVKFN<br>WYVDGVEVHNAKTKPREEQYASTYRVVSVLTVLHQDWLNGKEYKCKVSNKALPAPIEKTISK<br>AKGQPREPQVYTLPPSRDELTKNQVSLTCLVKGFYPSDIAVEWESNGQPENNYKTTPPVLDS<br>DGSFFLYSKLTVDKSRWQQGNVFSCSVMHEALHNHYTQKSLSLSPGKEFDYKDDDDKLE
3xNb:BCMA(269)-TNC-pCR3	MNFGFSLIFLVLVLKGVQCEVKLVPRGTAVQLVESGGGLVQAGDSLRLTCTASGRAFSTYFM<br>AWFRQAPGKEREFVAGIAWSGGSTAYADSVKGRFTISRDNAKNTVYLQMNSLKSEDTAVYYC<br>ASRGIEVEEFGAWGQGTQVTVSSRSGGGGSGGGGSGGGGSGGGGSGGGGSAVQLVESGGGLV<br>QAGDSLRLTCTASGRAFSTYFMAWFRQAPGKEREFVAGIAWSGGSTAYADSVKGRFTISRDN<br>AKNTVYLQMNSLKSEDTAVYYCASRGIEVEEFGAWGQGTQVTVSSGGGGSGGGGSGGGGSGG<br>GGSGGGGSQLAVQLVESGGGLVQAGDSLRLTCTASGRAFSTYFMAWFRQAPGKEREFVAGIA<br>WSGGSTAYADSVKGRFTISRDNAKNTVYLQMNSLKSEDTAVYYCASRGIEVEEFGAWGQGTQ<br>VTVSSGSDYKDDDDKDIACGCAAAPDIKDLLSRLEELEGLVSSLREQGTG
3xNb:BCMA(269)-ALFA-GCN4-pCR3	MNFGFSLIFLVLVLKGVQCEVKLVPRGTAVQLVESGGGLVQAGDSLRLTCTASGRAFSTYFM<br>AWFRQAPGKEREFVAGIAWSGGSTAYADSVKGRFTISRDNAKNTVYLQMNSLKSEDTAVYYC<br>ASRGIEVEEFGAWGQGTQVTVSSRSGGGGSGGGGSGGGGSGGGGSGGGGSAVQLVESGGGLV<br>QAGDSLRLTCTASGRAFSTYFMAWFRQAPGKEREFVAGIAWSGGSTAYADSVKGRFTISRDN<br>AKNTVYLQMNSLKSEDTAVYYCASRGIEVEEFGAWGQGTQVTVSSGGGGSGGGGSGGGGSGG<br>GGSGGGGSQLAVQLVESGGGLVQAGDSLRLTCTASGRAFSTYFMAWFRQAPGKEREFVAGIA<br>WSGGSTAYADSVKGRFTISRDNAKNTVYLQMNSLKSEDTAVYYCASRGIEVEEFGAWGQGTQ<br>VTVSSGSPSRLEEELRRRLTEPELRLKQIEDKLEEILSKLYHIENELARIKKLLGEREFGLE

Nb:BCMA(269)-Fc-GpL-pCR3	MNFGFSLIFLVLVLKGVQCEVKLVPRQLAVQLVESGGGLVQAGDSLRLTCTASGRAFSTYFM<br>AWFRQAPGKEREFVAGIAWSGGSTAYADSVKGRFTISRDNAKNTVYLQMNSLKSEDTAVYYC<br>ASRGIEVEEFGAWGQGTQVTVSSGSKTHTCPPCPAPELLGGPSVFLFPPKPKDTLMISRTPE<br>VTCVVVDVSHEDPEVKFNWYVDGVEVHNAKTKPREEQYNSTYRVVSVLTVLHQDWLNGKEYK<br>CKVSNKALPAPIEKTISKAKGQPREPQVYTLPPSRDELTKNQVSLTCLVKGFYPSDIAVEWE<br>SNGQPENNYKTTPPVLDSDGSFFLYSKLTVDKSRWQQGNVFSCSVMHEALHNHYTQKSLSLS<br>PGKEFDYKDDDDKLEKPTENNEDFNIVAVASNFATTDLDADRGKLPGKKLPLEVLKEMEANA<br>RKAGCTRGCLICLSHIKCTPKMKKFIPGRCHTYEGDKESAQGGIGEAIVDIPEIPGFKDLEP<br>MEQFIAQVDLCVDCTTGCLKGLANVQCSDLLKKWLPQRCATFASKIQGQVDKIKGAGGD
3xNb:CD40(V12t)-ALFA-Fc(DANA)-pCR3	MNFGFSLIFLVLVLKGVQCEVKLVPRGTQVQLQESGGGLVQAGGSLRLSCAASGLVFKRYSM<br>NWYRQPPGQQRGLVASISDSGVSTNYADSVKGRFTISRDNAKNIGYLQMNSLKPEDTAVYYC<br>NMHTFWGQGTQVTVSSRSGGGGSGGGGSGGGGSGGGGSGGGGSQVQLQESGGGLVQAGGSLR<br>LSCAASGLVFKRYSMNWYRQPPGQQRGLVASISDSGVSTNYADSVKGRFTISRDNAKNIGYL<br>QMNSLKPEDTAVYYCNMHTFWGQGTQVTVSSGGGGSGGGGSGGGGSGGGGSGGGGSQLQVQL<br>QESGGGLVQAGGSLRLSCAASGLVFKRYSMNWYRQPPGQQRGLVASISDSGVSTNYADSVKG<br>RFTISRDNAKNIGYLQMNSLKPEDTAVYYCNMHTFWGQGTQVTVSSGSPSRLEEELRRRLTE<br>PEFKTHTCPPCPAPELLGGPSVFLFPPKPKDTLMISRTPEVTCVVVAVSHEDPEVKFNWYVD<br>GVEVHNAKTKPREEQYASTYRVVSVLTVLHQDWLNGKEYKCKVSNKALPAPIEKTISKAKGQ<br>PREPQVYTLPPSRDELTKNQVSLTCLVKGFYPSDIAVEWESNGQPENNYKTTPPVLDSDGSF<br>FLYSKLTVDKSRWQQGNVFSCSVMHEALHNHYTQKSLSLSPGKLE
3xNb:CD40(V12t)-ALFA-TNC-pCR3	MNFGFSLIFLVLVLKGVQCEVKLVPRGTQVQLQESGGGLVQAGGSLRLSCAASGLVFKRYSM<br>NWYRQPPGQQRGLVASISDSGVSTNYADSVKGRFTISRDNAKNIGYLQMNSLKPEDTAVYYC<br>NMHTFWGQGTQVTVSSRSGGGGSGGGGSGGGGSGGGGSGGGGSQVQLQESGGGLVQAGGSLR<br>LSCAASGLVFKRYSMNWYRQPPGQQRGLVASISDSGVSTNYADSVKGRFTISRDNAKNIGYL<br>QMNSLKPEDTAVYYCNMHTFWGQGTQVTVSSGGGGSGGGGSGGGGSGGGGSGGGGSQLQVQL<br>QESGGGLVQAGGSLRLSCAASGLVFKRYSMNWYRQPPGQQRGLVASISDSGVSTNYADSVKG<br>RFTISRDNAKNIGYLQMNSLKPEDTAVYYCNMHTFWGQGTQVTVSSGSPSRLEEELRRRLTE<br>PEFDIACGCAAAPDIKDLLSRLEELEGLVSSLREQGTGLE
3xNb:CD40(V12t)-GCN4-pCR3	MNFGFSLIFLVLVLKGVQCEVKLVPRGSDYKDDDDKQLRLKQIEDKLEEILSKLYHIENELA<br>RIKKLLGEREFGLEQVQLQESGGGLVQAGGSLRLSCAASGLVFKRYSMNWYRQPPGQQRGLV<br>ASISDSGVSTNYADSVKGRFTISRDNAKNIGYLQMNSLKPEDTAVYYCNMHTFWGQGTQVTV<br>SSRSGGGGSGGGGSGGGGSGGGGSGGGGSQVQLQESGGGLVQAGGSLRLSCAASGLVFKRYS<br>MNWYRQPPGQQRGLVASISDSGVSTNYADSVKGRFTISRDNAKNIGYLQMNSLKPEDTAVYY<br>CNMHTFWGQGTQVTVSSGGGGSGGGGSGGGGSGGGGSGGGGSQLQVQLQESGGGLVQAGGSL<br>RLSCAASGLVFKRYSMNWYRQPPGQQRGLVASISDSGVSTNYADSVKGRFTISRDNAKNIGY<br>LQMNSLKPEDTAVYYCNMHTFWGQGTQVTVSS
3xNb:CD40(V12t)-ALFA-GCN4-pCR3	MNFGFSLIFLVLVLKGVQCEVKLVPRGTQVQLQESGGGLVQAGGSLRLSCAASGLVFKRYSM<br>NWYRQPPGQQRGLVASISDSGVSTNYADSVKGRFTISRDNAKNIGYLQMNSLKPEDTAVYYC<br>NMHTFWGQGTQVTVSSRSGGGGSGGGGSGGGGSGGGGSGGGGSQVQLQESGGGLVQAGGSLR<br>LSCAASGLVFKRYSMNWYRQPPGQQRGLVASISDSGVSTNYADSVKGRFTISRDNAKNIGYL<br>QMNSLKPEDTAVYYCNMHTFWGQGTQVTVSSGGGGSGGGGSGGGGSGGGGSGGGGSQLQVQL<br>QESGGGLVQAGGSLRLSCAASGLVFKRYSMNWYRQPPGQQRGLVASISDSGVSTNYADSVKG<br>RFTISRDNAKNIGYLQMNSLKPEDTAVYYCNMHTFWGQGTQVTVSSGSPSRLEEELRRRLTE<br>PELRLKQIEDKLEEILSKLYHIENELARIKKLLGEREFGLE
Nb:CD40(V12t)-Fc-GpL-pCR3	MNFGFSLIFLVLVLKGVQCEVKLVPRQLQVQLQESGGGLVQAGGSLRLSCAASGLVFKRYSM<br>NWYRQPPGQQRGLVASISDSGVSTNYADSVKGRFTISRDNAKNIGYLQMNSLKPEDTAVYYC<br>NMHTFWGQGTQVTVSSGSKTHTCPPCPAPELLGGPSVFLFPPKPKDTLMISRTPEVTCVVVD<br>VSHEDPEVKFNWYVDGVEVHNAKTKPREEQYNSTYRVVSVLTVLHQDWLNGKEYKCKVSNKA<br>LPAPIEKTISKAKGQPREPQVYTLPPSRDELTKNQVSLTCLVKGFYPSDIAVEWESNGQPEN<br>NYKTTPPVLDSDGSFFLYSKLTVDKSRWQQGNVFSCSVMHEALHNHYTQKSLSLSPGKEFDY<br>KDDDDKLEKPTENNEDFNIVAVASNFATTDLDADRGKLPGKKLPLEVLKEMEANARKAGCTR<br>GCLICLSHIKCTPKMKKFIPGRCHTYEGDKESAQGGIGEAIVDIPEIPGFKDLEPMEQFIAQ<br>VDLCVDCTTGCLKGLANVQCSDLLKKWLPQRCATFASKIQGQVDKIKGAGGD
3xNb:CD40(mu)(JPP-G1)-Fc(DANA)-pCR3	MNFGFSLIFLVLVLKGVQCEVKLVPRGTQVQLVESGGGLVQPGGSLRLSCVASGFTFNGDTM<br>IWYRQTPGKTREWVAGIAPIGDIANYANSVKGRFTISRDNAKNTVYLQMNSLRPEDTAVYYC<br>NTVTWGQSNSYWGQGTQVTVSSEPKTPKPQRSGGGGSGGGGSGGGGSGGGGSGGGGSQVQLV<br>ESGGGLVQPGGSLRLSCVASGFTFNGDTMIWYRQTPGKTREWVAGIAPIGDIANYANSVKGR<br>FTISRDNAKNTVYLQMNSLRPEDTAVYYCNTVTWGQSNSYWGQGTQVTVSSEPKTPKPQGGG<br>GSGGGGSGGGGSGGGGSGGGGSQLQVQLVESGGGLVQPGGSLRLSCVASGFTFNGDTMIWYR<br>QTPGKTREWVAGIAPIGDIANYANSVKGRFTISRDNAKNTVYLQMNSLRPEDTAVYYCNTVT<br>WGQSNSYWGQGTQVTVSSEPKTPKPQGSKTHTCPPCPAPELLGGPSVFLFPPKPKDTLMISR<br>TPEVTCVVVAVSHEDPEVKFNWYVDGVEVHNAKTKPREEQYASTYRVVSVLTVLHQDWLNGK<br>EYKCKVSNKALPAPIEKTISKAKGQPREPQVYTLPPSRDELTKNQVSLTCLVKGFYPSDIAV<br>EWESNGQPENNYKTTPPVLDSDGSFFLYSKLTVDKSRWQQGNVFSCSVMHEALHNHYTQKSL<br>SLSPGKEFDYKDDDDKLE
3xNb:CD40(mu)(JPP-G1)-TNC-pCR3	MNFGFSLIFLVLVLKGVQCEVKLVPRGTQVQLVESGGGLVQPGGSLRLSCVASGFTFNGDTM<br>IWYRQTPGKTREWVAGIAPIGDIANYANSVKGRFTISRDNAKNTVYLQMNSLRPEDTAVYYC<br>NTVTWGQSNSYWGQGTQVTVSSEPKTPKPQRSGGGGSGGGGSGGGGSGGGGSGGGGSQVQLV<br>ESGGGLVQPGGSLRLSCVASGFTFNGDTMIWYRQTPGKTREWVAGIAPIGDIANYANSVKGR<br>FTISRDNAKNTVYLQMNSLRPEDTAVYYCNTVTWGQSNSYWGQGTQVTVSSEPKTPKPQGGG<br>GSGGGGSGGGGSGGGGSGGGGSQLQVQLVESGGGLVQPGGSLRLSCVASGFTFNGDTMIWYR<br>QTPGKTREWVAGIAPIGDIANYANSVKGRFTISRDNAKNTVYLQMNSLRPEDTAVYYCNTVT<br>WGQSNSYWGQGTQVTVSSEPKTPKPQGSDYKDDDDKDIACGCAAAPDIKDLLSRLEELEGLV<br>SSLREQGTG
3xNb:CD40(mu)(JPP-G1)-ALFA-GCN4-pCR3	MNFGFSLIFLVLVLKGVQCEVKLVPRGTQVQLVESGGGLVQPGGSLRLSCVASGFTFNGDTM<br>IWYRQTPGKTREWVAGIAPIGDIANYANSVKGRFTISRDNAKNTVYLQMNSLRPEDTAVYYC<br>NTVTWGQSNSYWGQGTQVTVSSEPKTPKPQRSGGGGSGGGGSGGGGSGGGGSGGGGSQVQLV

	ESGGGLVQPGGSLRLSCVASGFTFNGDTMIWYRQTPGKTREWVAGIAPIGDIANYANSVKGRFTISRDNAKNTVYLQMNSLRPEDTAVYYCNTVTWGQSNSYWGQGTQVTVSSEPKTPKPQGGGGSGGGGSGGGGSGGGGSGGGGSQLQVQLVESGGGLVQPGGSLRLSCVASGFTFNGDTMIWYRQTPGKTREWVAGIAPIGDIANYANSVKGRFTISRDNAKNTVYLQMNSLRPEDTAVYYCNTVTWGQSNSYWGQGTQVTVSSEPKTPKPQGSPSRLEEELRRRLTEPELRLKQIEDKLEEILSKLYHIENELARIKKLLGEREFGLE
Nb:CD40(mu)(JPP-G1)-Fc-GpL-pCR3	MNFGFSLIFLVLVLKGVQCEVKLVPRQLQVQLVESGGGLVQPGGSLRLSCVASGFTFNGDTMIWYRQTPGKTREWVAGIAPIGDIANYANSVKGRFTISRDNAKNTVYLQMNSLRPEDTAVYYCNTVTWGQSNSYWGQGTQVTVSSEPKTPKPQGSKTHTCPPCPAPELLGGPSVFLFPPKPKDTLMISRTPEVTCVVVDVSHEDPEVKFNWYVDGVEVHNAKTKPREEQYNSTYRVVSVLTVLHQDWLNGKEYKCKVSNKALPAPIEKTISKAKGQPREPQVYTLPPSRDELTKNQVSLTCLVKGFYPSDIAVEWESNGQPENNYKTTPPVLDSDGSFFLYSKLTVDKSRWQQGNVFSCSVMHEALHNHYTQKSLSLSPGKEFDYKDDDDKLEKPTENNEDFNIVAVASNFATTDLDADRGKLPGKKLPLEVLKEMEANARKAGCTRGCLICLSHIKCTPKMKKFIPGRCHTYEGDKESAQGGIGEAIVDIPEIPGFKDLEPMEQFIAQVDLCVDCTTGCLKGLANVQCSDLLKKWLPQRCATFASKIQGQVDKIKGAGGD
3xNb:CD95(1G4)-ALFA-Fc(DANA)-pCR3	MNFGFSLIFLVLVLKGVQCEVKLVPRGTEVQLVESGGGLVQAGGSLRLSCAVSGFTDDDYYIGWFRQAPGMEREGVATISLGDNDTYYSHSVKGRFTISSDNAKNTVYLQMNNLKPEDTGVYTCAADRRPSSYSGSLSPIPYEYWGQGTQVTVSSAHHSEDPNSRSGGGGSGGGGSGGGGSGGGGSGGGGSEVQLVESGGGLVQAGGSLRLSCAVSGFTDDDYYIGWFRQAPGMEREGVATISLGDNDTYYSHSVKGRFTISSDNAKNTVYLQMNNLKPEDTGVYTCAADRRPSSYSGSLSPIPYEYWGQGTQVTVSSAHHSEDPNSGGGGSGGGGSGGGGSGGGGSGGGGSQLEVQLVESGGGLVQAGGSLRLSCAVSGFTDDDYYIGWFRQAPGMEREGVATISLGDNDTYYSHSVKGRFTISSDNAKNTVYLQMNNLKPEDTGVYTCAADRRPSSYSGSLSPIPYEYWGQGTQVTVSSAHHSEDPNSGSPSRLEEELRRRLTEPEFKTHTCPPCPAPELLGGPSVFLFPPKPKDTLMISRTPEVTCVVVAVSHEDPEVKFNWYVDGVEVHNAKTKPREEQYASTYRVVSVLTVLHQDWLNGKEYKCKVSNKALPAPIEKTISKAKGQPREPQVYTLPPSRDELTKNQVSLTCLVKGFYPSDIAVEWESNGQPENNYKTTPPVLDSDGSFFLYSKLTVDKSRWQQGNVFSCSVMHEALHNHYTQKSLSLSPGKLE
3xNb:CD95(1G4)-ALFA-TNC-pCR3	MNFGFSLIFLVLVLKGVQCEVKLVPRGTEVQLVESGGGLVQAGGSLRLSCAVSGFTDDDYYIGWFRQAPGMEREGVATISLGDNDTYYSHSVKGRFTISSDNAKNTVYLQMNNLKPEDTGVYTCAADRRPSSYSGSLSPIPYEYWGQGTQVTVSSAHHSEDPNSRSGGGGSGGGGSGGGGSGGGGSGGGGSEVQLVESGGGLVQAGGSLRLSCAVSGFTDDDYYIGWFRQAPGMEREGVATISLGDNDTYYSHSVKGRFTISSDNAKNTVYLQMNNLKPEDTGVYTCAADRRPSSYSGSLSPIPYEYWGQGTQVTVSSAHHSEDPNSGGGGSGGGGSGGGGSGGGGSGGGGSQLEVQLVESGGGLVQAGGSLRLSCAVSGFTDDDYYIGWFRQAPGMEREGVATISLGDNDTYYSHSVKGRFTISSDNAKNTVYLQMNNLKPEDTGVYTCAADRRPSSYSGSLSPIPYEYWGQGTQVTVSSAHHSEDPNSGSPSRLEEELRRRLTEPEFDIACGCAAAPDIKDLLSRLEELEGLVSSLREQGTGLE
3xNb:CD95(1G4)-ALFA-GCN4-pCR3	MNFGFSLIFLVLVLKGVQCEVKLVPRGTEVQLVESGGGLVQAGGSLRLSCAVSGFTDDDYYIGWFRQAPGMEREGVATISLGDNDTYYSHSVKGRFTISSDNAKNTVYLQMNNLKPEDTGVYTCAADRRPSSYSGSLSPIPYEYWGQGTQVTVSSAHHSEDPNSRSGGGGSGGGGSGGGGSGGGGSGGGGSEVQLVESGGGLVQAGGSLRLSCAVSGFTDDDYYIGWFRQAPGMEREGVATISLGDNDTYYSHSVKGRFTISSDNAKNTVYLQMNNLKPEDTGVYTCAADRRPSSYSGSLSPIPYEYWGQGTQVTVSSAHHSEDPNSGGGGSGGGGSGGGGSGGGGSGGGGSQLEVQLVESGGGLVQAGGSLRLSCAVSGFTDDDYYIGWFRQAPGMEREGVATISLGDNDTYYSHSVKGRFTISSDNAKNTVYLQMNNLKPEDTGVYTCAADRRPSSYSGSLSPIPYEYWGQGTQVTVSSAHHSEDPNSGSPSRLEEELRRRLTEPELRLKQIEDKLEEILSKLYHIENELARIKKLLGEREFGLE
3xNb:CD95(1G4)-GCN4-pCR3	MNFGFSLIFLVLVLKGVQCEVKLVPRGTEVQLVESGGGLVQAGGSLRLSCAVSGFTDDDYYIGWFRQAPGMEREGVATISLGDNDTYYSHSVKGRFTISSDNAKNTVYLQMNNLKPEDTGVYTCAADRRPSSYSGSLSPIPYEYWGQGTQVTVSSAHHSEDPNSRSGGGGSGGGGSGGGGSGGGGSGGGGSEVQLVESGGGLVQAGGSLRLSCAVSGFTDDDYYIGWFRQAPGMEREGVATISLGDNDTYYSHSVKGRFTISSDNAKNTVYLQMNNLKPEDTGVYTCAADRRPSSYSGSLSPIPYEYWGQGTQVTVSSAHHSEDPNSGGGGSGGGGSGGGGSGGGGSGGGGSQLEVQLVESGGGLVQAGGSLRLSCAVSGFTDDDYYIGWFRQAPGMEREGVATISLGDNDTYYSHSVKGRFTISSDNAKNTVYLQMNNLKPEDTGVYTCAADRRPSSYSGSLSPIPYEYWGQGTQVTVSSAHHSEDPNSGSDYKDDDDKQLRLKQIEDKLEEILSKLYHIENELARIKKLLGEREFGLE
Nb:CD95(1G4)-Fc-GpL-pCR3	MNFGFSLIFLVLVLKGVQCEVKLVPRQLEVQLVESGGGLVQAGGSLRLSCAVSGFTDDDYYIGWFRQAPGMEREGVATISLGDNDTYYSHSVKGRFTISSDNAKNTVYLQMNNLKPEDTGVYTCAADRRPSSYSGSLSPIPYEYWGQGTQVTVSSAHHSEDPNSGSKTHTCPPCPAPELLGGPSVFLFPPKPKDTLMISRTPEVTCVVVDVSHEDPEVKFNWYVDGVEVHNAKTKPREEQYNSTYRVVSVLTVLHQDWLNGKEYKCKVSNKALPAPIEKTISKAKGQPREPQVYTLPPSRDELTKNQVSLTCLVKGFYPSDIAVEWESNGQPENNYKTTPPVLDSDGSFFLYSKLTVDKSRWQQGNVFSCSVMHEALHNHYTQKSLSLSPGKEFDYKDDDDKLEKPTENNEDFNIVAVASNFATTDLDADRGKLPGKKLPLEVLKEMEANARKAGCTRGCLICLSHIKCTPKMKKFIPGRCHTYEGDKESAQGGIGEAIVDIPEIPGFKDLEPMEQFIAQVDLCVDCTTGCLKGLANVQCSDLLKKWLPQRCATFASKIQGQVDKIKGAGGD
3xNb:CD95(mu)(1A3)-Fc-GpL-pCR3	MNFGFSLIFLVLVLKGVQCEVKLVPRGTEVQLQESGGGLVQPGGSLRLSCVGSGFDFSEKVMGWARRVRGKGLEWVSGIYSHGTVAYYADSVKGRFTISRDNAKNTMYLQMNSLEPEDTGVYYCAIYPPRGSSWSRAIMEYTGQGTQVTVSSRSGGGGSGGGGSGGGGSGGGGSGGGGSEVQLQESGGGLVQPGGSLRLSCVGSGFDFSEKVMGWARRVRGKGLEWVSGIYSHGTVAYYADSVKGRFTISRDNAKNTMYLQMNSLEPEDTGVYYCAIYPPRGSSWSRAIMEYTGQGTQVTVSSGGGGSGGGGSGGGGSGGGGSGGGGSQLEVQLQESGGGLVQPGGSLRLSCVGSGFDFSEKVMGWARRVRGKGLEWVSGIYSHGTVAYYADSVKGRFTISRDNAKNTMYLQMNSLEPEDTGVYYCAIYPPRGSSWSRAIMEYTGQGTQVTVSSGSKTHTCPPCPAPELLGGPSVFLFPPKPKDTLMISRTPEVTCVVVDVSHEDPEVKFNWYVDGVEVHNAKTKPREEQYNSTYRVVSVLTVLHQDWLNGKEYKCKVSNKALPAPIEKTISKAKGQPREPQVYTLPPSRDELTKNQVSLTCLVKGFYPSDIAVEWESNGQPENNYKTTPPVLDSDGSFFLYSKLTVDKSRWQQGNVFSCSVMHEALHNHYTQKSLSLSPGKEFDYKDDDDKLEKPTENNEDFNIVAVASNFATTDLDADRGKLPGKKLPLEVLKEMEANARKA

	GCTRGCLICLSHIKCTPKMKKFIPGRCHTYEGDKESAQGGIGEAIVDIPEIPGFKDLEPMEQFIAQVDLCVDCTTGCLKGLANVQCSDLLKKWLPQRCATFASKIQGQVDKIKGAGGD
3xNb:CD95(mu)(1A3)-ALFA-TNC-pCR3	MNFGFSLIFLVLVLKGVQCEVKLVPRGTEVQLQESGGGLVQPGGSLRLSCVGSGFDFSEKVMGWARRVRGKGLEWVSGIYSHGTVAYYADSVKGRFTISRDNAKNTMYLQMNSLEPEDTGVYYCAIYPPRGSSWSRAIMEYTGQGTQVTVSSRSGGGGSGGGGSGGGGSGGGGSGGGGSEVQLQESGGGLVQPGGSLRLSCVGSGFDFSEKVMGWARRVRGKGLEWVSGIYSHGTVAYYADSVKGRFTISRDNAKNTMYLQMNSLEPEDTGVYYCAIYPPRGSSWSRAIMEYTGQGTQVTVSSGGGGSGGGGSGGGGSGGGGSGGGGSQLEVQLQESGGGLVQPGGSLRLSCVGSGFDFSEKVMGWARRVRGKGLEWVSGIYSHGTVAYYADSVKGRFTISRDNAKNTMYLQMNSLEPEDTGVYYCAIYPPRGSSWSRAIMEYTGQGTQVTVSSGSPSRLEEELRRRLTEPEFDIACGCAAAPDIKDLLSRLEELEGLVSSLREQGTGLE
3xNb:CD95(mu)(1A3)-ALFA-GCN4-pCR3	MNFGFSLIFLVLVLKGVQCEVKLVPRGTEVQLQESGGGLVQPGGSLRLSCVGSGFDFSEKVMGWARRVRGKGLEWVSGIYSHGTVAYYADSVKGRFTISRDNAKNTMYLQMNSLEPEDTGVYYCAIYPPRGSSWSRAIMEYTGQGTQVTVSSRSGGGGSGGGGSGGGGSGGGGSGGGGSEVQLQESGGGLVQPGGSLRLSCVGSGFDFSEKVMGWARRVRGKGLEWVSGIYSHGTVAYYADSVKGRFTISRDNAKNTMYLQMNSLEPEDTGVYYCAIYPPRGSSWSRAIMEYTGQGTQVTVSSGGGGSGGGGSGGGGSGGGGSGGGGSQLEVQLQESGGGLVQPGGSLRLSCVGSGFDFSEKVMGWARRVRGKGLEWVSGIYSHGTVAYYADSVKGRFTISRDNAKNTMYLQMNSLEPEDTGVYYCAIYPPRGSSWSRAIMEYTGQGTQVTVSSGSPSRLEEELRRRLTEPELRLKQIEDKLEEILSKLYHIENELARIKKLLGEREFGLE
Nb:CD95(mu)(1A3)-Fc-GpL-pCR3	MNFGFSLIFLVLVLKGVQCEVKLVPRQLEVQLQESGGGLVQPGGSLRLSCVGSGFDFSEKVMGWARRVRGKGLEWVSGIYSHGTVAYYADSVKGRFTISRDNAKNTMYLQMNSLEPEDTGVYYCAIYPPRGSSWSRAIMEYTGQGTQVTVSSGSKTHTCPPCPAPELLGGPSVFLFPPKPKDTLMISRTPEVTCVVVDVSHEDPEVKFNWYVDGVEVHNAKTKPREEQYNSTYRVVSVLTVLHQDWLNGKEYKCKVSNKALPAPIEKTISKAKGQPREPQVYTLPPSRDELTKNQVSLTCLVKGFYPSDIAVEWESNGQPENNYKTTPPVLDSDGSFFLYSKLTVDKSRWQQGNVFSCSVMHEALHNHYTQKSLSLSPGKEFDYKDDDDKLEKPTENNEDFNIVAVASNFATTDLDADRGKLPGKKLPLEVLKEMEANARKAGCTRGCLICLSHIKCTPKMKKFIPGRCHTYEGDKESAQGGIGEAIVDIPEIPGFKDLEPMEQFIAQVDLCVDCTTGCLKGLANVQCSDLLKKWLPQRCATFASKIQGQVDKIKGAGGD
3xNb:GITR(C06)-Fc-GpL-pCR3	MNFGFSLIFLVLVLKGVQCEVKLVPRGTEVQLLESGGGEVQPGGSLRLSCAASGSVFSIDAMGWYRQAPGKGLELVSALSGISSATYAESVKGRFTISRDNAKNTLYLQMSSLRAEDTAVYYCYADVSTGWGRDAHGYWGQGTLVTVRSGGGGSGGGGSGGGGSGGGGSGGGGSEVQLLESGGGEVQPGGSLRLSCAASGSVFSIDAMGWYRQAPGKGLELVSALSGISSATYAESVKGRFTISRDNAKNTLYLQMSSLRAEDTAVYYCYADVSTGWGRDAHGYWGQGTLVTVGGGGSGGGGSGGGGSGGGGSGGGGSQLEVQLLESGGGEVQPGGSLRLSCAASGSVFSIDAMGWYRQAPGKGLELVSALSGISSATYAESVKGRFTISRDNAKNTLYLQMSSLRAEDTAVYYCYADVSTGWGRDAHGYWGQGTLVTVGSKTHTCPPCPAPELLGGPSVFLFPPKPKDTLMISRTPEVTCVVVDVSHEDPEVKFNWYVDGVEVHNAKTKPREEQYNSTYRVVSVLTVLHQDWLNGKEYKCKVSNKALPAPIEKTISKAKGQPREPQVYTLPPSRDELTKNQVSLTCLVKGFYPSDIAVEWESNGQPENNYKTTPPVLDSDGSFFLYSKLTVDKSRWQQGNVFSCSVMHEALHNHYTQKSLSLSPGKEFDYKDDDDKLEKPTENNEDFNIVAVASNFATTDLDADRGKLPGKKLPLEVLKEMEANARKAGCTRGCLICLSHIKCTPKMKKFIPGRCHTYEGDKESAQGGIGEAIVDIPEIPGFKDLEPMEQFIAQVDLCVDCTTGCLKGLANVQCSDLLKKWLPQRCATFASKIQGQVDKIKGAGGD
3xNb:GITR(C06)-TNC-pCR3	MNFGFSLIFLVLVLKGVQCEVKLVPRGTEVQLLESGGGEVQPGGSLRLSCAASGSVFSIDAMGWYRQAPGKGLELVSALSGISSATYAESVKGRFTISRDNAKNTLYLQMSSLRAEDTAVYYCYADVSTGWGRDAHGYWGQGTLVTVRSGGGGSGGGGSGGGGSGGGGSGGGGSEVQLLESGGGEVQPGGSLRLSCAASGSVFSIDAMGWYRQAPGKGLELVSALSGISSATYAESVKGRFTISRDNAKNTLYLQMSSLRAEDTAVYYCYADVSTGWGRDAHGYWGQGTLVTVGGGGSGGGGSGGGGSGGGGSGGGGSQLEVQLLESGGGEVQPGGSLRLSCAASGSVFSIDAMGWYRQAPGKGLELVSALSGISSATYAESVKGRFTISRDNAKNTLYLQMSSLRAEDTAVYYCYADVSTGWGRDAHGYWGQGTLVTVGSDYKDDDDKDIACGCAAAPDIKDLLSRLEELEGLVSSLREQGTG
3xNb:GITR(C06)-ALFA-GCN4-pCR3	MNFGFSLIFLVLVLKGVQCEVKLVPRGTEVQLLESGGGEVQPGGSLRLSCAASGSVFSIDAMGWYRQAPGKGLELVSALSGISSATYAESVKGRFTISRDNAKNTLYLQMSSLRAEDTAVYYCYADVSTGWGRDAHGYWGQGTLVTVRSGGGGSGGGGSGGGGSGGGGSGGGGSEVQLLESGGGEVQPGGSLRLSCAASGSVFSIDAMGWYRQAPGKGLELVSALSGISSATYAESVKGRFTISRDNAKNTLYLQMSSLRAEDTAVYYCYADVSTGWGRDAHGYWGQGTLVTVGGGGSGGGGSGGGGSGGGGSGGGGSQLEVQLLESGGGEVQPGGSLRLSCAASGSVFSIDAMGWYRQAPGKGLELVSALSGISSATYAESVKGRFTISRDNAKNTLYLQMSSLRAEDTAVYYCYADVSTGWGRDAHGYWGQGTLVTVGSPSRLEEELRRRLTEPELRLKQIEDKLEEILSKLYHIENELARIKKLLGEREFGLE
3xNb:GITR(C06)-Fc-GpL-pCR3	MNFGFSLIFLVLVLKGVQCEVKLVPRQLEVQLLESGGGEVQPGGSLRLSCAASGSVFSIDAMGWYRQAPGKGLELVSALSGISSATYAESVKGRFTISRDNAKNTLYLQMSSLRAEDTAVYYCYADVSTGWGRDAHGYWGQGTLVTVGSKTHTCPPCPAPELLGGPSVFLFPPKPKDTLMISRTPEVTCVVVDVSHEDPEVKFNWYVDGVEVHNAKTKPREEQYNSTYRVVSVLTVLHQDWLNGKEYKCKVSNKALPAPIEKTISKAKGQPREPQVYTLPPSRDELTKNQVSLTCLVKGFYPSDIAVEWESNGQPENNYKTTPPVLDSDGSFFLYSKLTVDKSRWQQGNVFSCSVMHEALHNHYTQKSLSLSPGKEFDYKDDDDKLEKPTENNEDFNIVAVASNFATTDLDADRGKLPGKKLPLEVLKEMEANARKAGCTRGCLICLSHIKCTPKMKKFIPGRCHTYEGDKESAQGGIGEAIVDIPEIPGFKDLEPMEQFIAQVDLCVDCTTGCLKGLANVQCSDLLKKWLPQRCATFASKIQGQVDKIKGAGGD
3xNb:OX40(V1)-Fc(DANA)-pCR3	MNFGFSLIFLVLVLKGVQCEVKLVPRGTEVQLLESGGGEVQPGGSLRLSCAASGFTFSDAFMYWVRQAPGKGLEWVSSISNRGLKTAYAESVKGRFTISRDNAKNTLYLQMSSLRAEDTAVYYCSRDVDGDFRGQGTLVTVKPRSGGGGSGGGGSGGGGSGGGGSGGGGSEVQLLESGGGEVQPGGSLRLSCAASGFTFSDAFMYWVRQAPGKGLEWVSSISNRGLKTAYAESVKGRFTISRDNAKNTLYLQMSSLRAEDTAVYYCSRDVDGDFRGQGTLVTVKPGGGGSGGGGSGGGGSGGGSSGGGGSQLEVQLLESGGGEVQPGGSLRLSCAASGFTFSDAFMYWVRQAPGKGLEWVSSISNRGLKTAYAESVKGRFTISRDNAKNTLYLQMSSLRAEDTAVYYCSRDVDGDFRGQGTLVTVKPGSKTHTCPPCPAPELLGGPSVFLFPPKPKDTLMISRTPEVTCVVVAVSHEDPEVKFNWYVDGVEVHNAK

	TKPREEQYASTYRVVSVLTVLHQDWLNGKEYKCKVSNKALPAPIEKTISKAKGQPREPQVYTLPPSRDELTKNQVSLTCLVKGFYPSDIAVEWESNGQPENNYKTTPPVLDSDGSFFLYSKLTVDKSRWQQGNVFSCSVMHEALHNHYTQKSLSLSPGKEFDYKDDDDKLE
3xNb:OX40(V1)-TNC-pCR3	MNFGFSLIFLVLVLKGVQCEVKLVPRGTEVQLLESGGGEVQPGGSLRLSCAASGFTFSDAFMYWVRQAPGKGLEWVSSISNRGLKTAYAESVKGRFTISRDNAKNTLYLQMSSLRAEDTAVYYCSRDVDGDFRGQGTLVTVKPRSGGGGSGGGGSGGGGSGGGGSGGGGSEVQLLESGGGEVQPGGSLRLSCAASGFTFSDAFMYWVRQAPGKGLEWVSSISNRGLKTAYAESVKGRFTISRDNAKNTLYLQMSSLRAEDTAVYYCSRDVDGDFRGQGTLVTVKPGGGGSGGGGSGGGGSGGGGSGGGGSQLEVQLLESGGGEVQPGGSLRLSCAASGFTFSDAFMYWVRQAPGKGLEWVSSISNRGLKTAYAESVKGRFTISRDNAKNTLYLQMSSLRAEDTAVYYCSRDVDGDFRGQGTLVTVKPGSDYKDDDDKDIACGCAAAPDIKDLLSRLEELEGLVSSLREQGTG
3xNb:OX40(V1)-ALFA-GCN4-pCR3	MNFGFSLIFLVLVLKGVQCEVKLVPRGTEVQLLESGGGEVQPGGSLRLSCAASGFTFSDAFMYWVRQAPGKGLEWVSSISNRGLKTAYAESVKGRFTISRDNAKNTLYLQMSSLRAEDTAVYYCSRDVDGDFRGQGTLVTVKPRSGGGGSGGGGSGGGGSGGGGSGGGGSEVQLLESGGGEVQPGGSLRLSCAASGFTFSDAFMYWVRQAPGKGLEWVSSISNRGLKTAYAESVKGRFTISRDNAKNTLYLQMSSLRAEDTAVYYCSRDVDGDFRGQGTLVTVKPGGGGSGGGGSGGGGSGGGGSGGGGSQLEVQLLESGGGEVQPGGSLRLSCAASGFTFSDAFMYWVRQAPGKGLEWVSSISNRGLKTAYAESVKGRFTISRDNAKNTLYLQMSSLRAEDTAVYYCSRDVDGDFRGQGTLVTVKPGSPSRLEEELRRRLTEPELRLKQIEDKLEEILSKLYHIENELARIKKLLGEREFGLE
Nb:OX40(V1)-Fc-GpL-pCR3	MNFGFSLIFLVLVLKGVQCEVKLVPRQLEVQLLESGGGEVQPGGSLRLSCAASGFTFSDAFMYWVRQAPGKGLEWVSSISNRGLKTAYAESVKGRFTISRDNAKNTLYLQMSSLRAEDTAVYYCSRDVDGDFRGQGTLVTVKPGSKTHTCPPCPAPELLGGPSVFLFPPKPKDTLMISRTPEVTCVVVDVSHEDPEVKFNWYVDGVEVHNAKTKPREEQYNSTYRVVSVLTVLHQDWLNGKEYKCKVSNKALPAPIEKTISKAKGQPREPQVYTLPPSRDELTKNQVSLTCLVKGFYPSDIAVEWESNGQPENNYKTTPPVLDSDGSFFLYSKLTVDKSRWQQGNVFSCSVMHEALHNHYTQKSLSLSPGKEFDYKDDDDKLEKPTENNEDFNIVAVASNFATTDLDADRGKLPGKKLPLEVLKEMEANARKAGCTRGCLICLSHIKCTPKMKKFIPGRCHTYEGDKESAQGGIGEAIVDIPEIPGFKDLEPMEQFIAQVDLCVDCTTGCLKGLANVQCSDLLKKWLPQRCATFASKIQGQVDKIKGAGGD
3xNb:TNFR1(NB70)-Fc-GpL-pCR3	MNFGFSLIFLVLVLKGVQCEVKLVPRGTQVQLQESGGGLVQAGGPLRLSCAASVRTFSSYAMGWFRQAPGKEREFVAAISWSGGSTYYADSVKGRFTISRDNAKNTVYLQMNSLKPEDTAVYYCAVLHEDLYEYDKWGQGTQVTVSSRSGGGGSGGGGSGGGGSGGGGSGGGGSQVQLQESGGGLVQAGGPLRLSCAASVRTFSSYAMGWFRQAPGKEREFVAAISWSGGSTYYADSVKGRFTISRDNAKNTVYLQMNSLKPEDTAVYYCAVLHEDLYEYDKWGQGTQVTVSSGGGGSGGGGSGGGGSGGGGSGGGGSQLQVQLQESGGGLVQAGGPLRLSCAASVRTFSSYAMGWFRQAPGKEREFVAAISWSGGSTYYADSVKGRFTISRDNAKNTVYLQMNSLKPEDTAVYYCAVLHEDLYEYDKWGQGTQVTVSSGSKTHTCPPCPAPELLGGPSVFLFPPKPKDTLMISRTPEVTCVVVDVSHEDPEVKFNWYVDGVEVHNAKTKPREEQYNSTYRVVSVLTVLHQDWLNGKEYKCKVSNKALPAPIEKTISKAKGQPREPQVYTLPPSRDELTKNQVSLTCLVKGFYPSDIAVEWESNGQPENNYKTTPPVLDSDGSFFLYSKLTVDKSRWQQGNVFSCSVMHEALHNHYTQKSLSLSPGKEFDYKDDDDKLEKPTENNEDFNIVAVASNFATTDLDADRGKLPGKKLPLEVLKEMEANARKAGCTRGCLICLSHIKCTPKMKKFIPGRCHTYEGDKESAQGGIGEAIVDIPEIPGFKDLEPMEQFIAQVDLCVDCTTGCLKGLANVQCSDLLKKWLPQRCATFASKIQGQVDKIKGAGGD
3xNb:TNFR1(NB70)-TNC-pCR3	MNFGFSLIFLVLVLKGVQCEVKLVPRGTQVQLQESGGGLVQAGGPLRLSCAASVRTFSSYAMGWFRQAPGKEREFVAAISWSGGSTYYADSVKGRFTISRDNAKNTVYLQMNSLKPEDTAVYYCAVLHEDLYEYDKWGQGTQVTVSSRSGGGGSGGGGSGGGGSGGGGSGGGGSQVQLQESGGGLVQAGGPLRLSCAASVRTFSSYAMGWFRQAPGKEREFVAAISWSGGSTYYADSVKGRFTISRDNAKNTVYLQMNSLKPEDTAVYYCAVLHEDLYEYDKWGQGTQVTVSSGGGGSGGGGSGGGGSGGGGSGGGGSQLQVQLQESGGGLVQAGGPLRLSCAASVRTFSSYAMGWFRQAPGKEREFVAAISWSGGSTYYADSVKGRFTISRDNAKNTVYLQMNSLKPEDTAVYYCAVLHEDLYEYDKWGQGTQVTVSSGSDYKDDDDKDIACGCAAAPDIKDLLSRLEELEGLVSSLREQGTG
3xNb:TNFR1(NB70)-ALFA-GCN4-pCR3	MNFGFSLIFLVLVLKGVQCEVKLVPRGTQVQLQESGGGLVQAGGPLRLSCAASVRTFSSYAMGWFRQAPGKEREFVAAISWSGGSTYYADSVKGRFTISRDNAKNTVYLQMNSLKPEDTAVYYCAVLHEDLYEYDKWGQGTQVTVSSRSGGGGSGGGGSGGGGSGGGGSGGGGSQVQLQESGGGLVQAGGPLRLSCAASVRTFSSYAMGWFRQAPGKEREFVAAISWSGGSTYYADSVKGRFTISRDNAKNTVYLQMNSLKPEDTAVYYCAVLHEDLYEYDKWGQGTQVTVSSGGGGSGGGGSGGGGSGGGGSGGGGSQLQVQLQESGGGLVQAGGPLRLSCAASVRTFSSYAMGWFRQAPGKEREFVAAISWSGGSTYYADSVKGRFTISRDNAKNTVYLQMNSLKPEDTAVYYCAVLHEDLYEYDKWGQGTQVTVSSGSPSRLEEELRRRLTEPELRLKQIEDKLEEILSKLYHIENELARIKKLLGEREFGLE
Nb:TNFR1(NB70)-Fc-GpL-pCR3	MNFGFSLIFLVLVLKGVQCEVKLVPRQLQVQLQESGGGLVQAGGPLRLSCAASVRTFSSYAMGWFRQAPGKEREFVAAISWSGGSTYYADSVKGRFTISRDNAKNTVYLQMNSLKPEDTAVYYCAVLHEDLYEYDKWGQGTQVTVSSGSKTHTCPPCPAPELLGGPSVFLFPPKPKDTLMISRTPEVTCVVVDVSHEDPEVKFNWYVDGVEVHNAKTKPREEQYNSTYRVVSVLTVLHQDWLNGKEYKCKVSNKALPAPIEKTISKAKGQPREPQVYTLPPSRDELTKNQVSLTCLVKGFYPSDIAVEWESNGQPENNYKTTPPVLDSDGSFFLYSKLTVDKSRWQQGNVFSCSVMHEALHNHYTQKSLSLSPGKEFDYKDDDDKLEKPTENNEDFNIVAVASNFATTDLDADRGKLPGKKLPLEVLKEMEANARKAGCTRGCLICLSHIKCTPKMKKFIPGRCHTYEGDKESAQGGIGEAIVDIPEIPGFKDLEPMEQFIAQVDLCVDCTTGCLKGLANVQCSDLLKKWLPQRCATFASKIQGQVDKIKGAGGD
3xNb:TNFR1(mu)-Fc-GpL-pCR3	MNFGFSLIFLVLVLKGVQCEVKLVPRGTEVQLLESGGGLVQPGGSLRLSCAASGFTFNRYSMGWLRQAPGKGLEWVSRIDSYGRGTYYEDPVKGRFSISRDNSKNTLYLQMNSLRAEDTAVYYCAKISQFGSNAFDYWGQGTQVTVSSRSGGGGSGGGGSGGGGSGGGGSGGGGSEVQLLESGGGLVQPGGSLRLSCAASGFTFNRYSMGWLRQAPGKGLEWVSRIDSYGRGTYYEDPVKGRFSISRDNSKNTLYLQMNSLRAEDTAVYYCAKISQFGSNAFDYWGQGTQVTVSSGGGGSGGGGSGGGGSGGGGSGGGGSQLEVQLLESGGGLVQPGGSLRLSCAASGFTFNRYSMGWLRQAPGKGLEWVSRIDSYGRGTYYEDPVKGRFSISRDNSKNTLYLQMNSLRAEDTAVYYCAKISQFGSNAFDYWGQGTQVTVSSGSKTHTCPPCPAPELLGGPSVFLFPPKPKDTLMISRTPEVTCVVVDVSHEDPEVKFNWYVDGVEVHNAKTKPREEQYNSTYRVVSVLTVLHQDWLNGKEYKCKVSNKALPAPIEKTISKAKGQPREPQVYTLPPSRDELTKNQVSLTCLVKGFYPSDIAVEWESNGQPENNYKTTPPV

	LDSDGSFFLYSKLTVDKSRWQQGNVFSCSVMHEALHNHYTQKSLSLSPGKEFDYKDDDDKLE<br>KPTENNEDFNIVAVASNFATTDLDADRGKLPGKKLPLEVLKEMEANARKAGCTRGCLICLSH<br>IKCTPKMKKFIPGRCHTYEGDKESAQGGIGEAIVDIPEIPGFKDLEPMEQFIAQVDLCVDCT<br>TGCLKGLANVQCSDLLKKWLPQRCATFASKIQGQVDKIKGAGGD
3xNb:TNFR1(mu)-TNC-pCR3	MNFGFSLIFLVLVLKGVQCEVKLVPRGTEVQLLESGGGLVQPGGSLRLSCAASGFTFNRYSM<br>GWLRQAPGKGLEWVSRIDSYGRGTYYEDPVKGRFSISRDNSKNTLYLQMNSLRAEDTAVYYC<br>AKISQFGSNAFDYWGQGTQVTVSSRSGGGGSGGGGSGGGGSGGGGSGGGGSEVQLLESGGGL<br>VQPGGSLRLSCAASGFTFNRYSMGWLRQAPGKGLEWVSRIDSYGRGTYYEDPVKGRFSISRD<br>NSKNTLYLQMNSLRAEDTAVYYCAKISQFGSNAFDYWGQGTQVTVSSGGGGSGGGGSGGGGS<br>GGGGSGGGGSQLEVQLLESGGGLVQPGGSLRLSCAASGFTFNRYSMGWLRQAPGKGLEWVSR<br>IDSYGRGTYYEDPVKGRFSISRDNSKNTLYLQMNSLRAEDTAVYYCAKISQFGSNAFDYWGQ<br>GTQVTVSSGSDYKDDDDKDIACGCAAAPDIKDLLSRLEELEGLVSSLREQGTG
3xNb:TNFR1(mu)-ALFA-GCN4-pCR3	MNFGFSLIFLVLVLKGVQCEVKLVPRGTEVQLLESGGGLVQPGGSLRLSCAASGFTFNRYSM<br>GWLRQAPGKGLEWVSRIDSYGRGTYYEDPVKGRFSISRDNSKNTLYLQMNSLRAEDTAVYYC<br>AKISQFGSNAFDYWGQGTQVTVSSRSGGGGSGGGGSGGGGSGGGGSGGGGSEVQLLESGGGL<br>VQPGGSLRLSCAASGFTFNRYSMGWLRQAPGKGLEWVSRIDSYGRGTYYEDPVKGRFSISRD<br>NSKNTLYLQMNSLRAEDTAVYYCAKISQFGSNAFDYWGQGTQVTVSSGGGGSGGGGSGGGGS<br>GGGGSGGGGSQLEVQLLESGGGLVQPGGSLRLSCAASGFTFNRYSMGWLRQAPGKGLEWVSR<br>IDSYGRGTYYEDPVKGRFSISRDNSKNTLYLQMNSLRAEDTAVYYCAKISQFGSNAFDYWGQ<br>GTQVTVSSGSPSRLEEELRRRLTEPELRLKQIEDKLEEILSKLYHIENELARIKKLLGEREF<br>GLE
Nb:TNFR1(mu)-Fc-GpL-pCR3	MNFGFSLIFLVLVLKGVQCEVKLVPRQFEVQLLESGGGLVQPGGSLRLSCAASGFTFNRYSM<br>GWLRQAPGKGLEWVSRIDSYGRGTYYEDPVKGRFSISRDNSKNTLYLQMNSLRAEDTAVYYC<br>AKISQFGSNAFDYWGQGTQVTVSSRSKTHTCPPCPAPELLGGPSVFLFPPKPKDTLMISRTP<br>EVTCVVVDVSHEDPEVKFNWYVDGVEVHNAKTKPREEQYNSTYRVVSVLTVLHQDWLNGKEY<br>KCKVSNKALPAPIEKTISKAKGQPREPQVYTLPPSRDELTKNQVSLTCLVKGFYPSDIAVEW<br>ESNGQPENNYKTTPPVLDSDGSFFLYSKLTVDKSRWQQGNVFSCSVMHEALHNHYTQKSLSL<br>SPGKEFDYKDDDDKLEKPTENNEDFNIVAVASNFATTDLDADRGKLPGKKLPLEVLKEMEAN<br>ARKAGCTRGCLICLSHIKCTPKMKKFIPGRCHTYEGDKESAQGGIGEAIVDIPEIPGFKDLE<br>PMEQFIAQVDLCVDCTTGCLKGLANVQCSDLLKKWLPQRCATFASKIQGQVDKIKGAGGD
3xNb:TRAILR2(F5)-Fc(DANA)-pCR3	MNFGFSLIFLVLVLKGVQCEVKLVPRGTQVQLVQSGGGLVQAGDSLRLSCAASGLTFPNYGM<br>GWFRQAPGEEREFLAWIYWSGGTVFYADSVKGRFTISRDAAKNMVYLQMNSLKSDDTAVYYC<br>AVTIRGAATQTWKYDYWGRGTQVTVSSRSGGGGSGGGGSGGGGSGGGGSGGGGSQVQLVQSG<br>GGLVQAGDSLRLSCAASGLTFPNYGMGWFRQAPGEEREFLAWIYWSGGTVFYADSVKGRFTI<br>SRDAAKNMVYLQMNSLKSDDTAVYYCAVTIRGAATQTWKYDYWGRGTQVTVSSGGGGSGGGG<br>SGGGGSGGGGSGGGGSQLQVQLVQSGGGLVQAGDSLRLSCAASGLTFPNYGMGWFRQAPGEE<br>REFLAWIYWSGGTVFYADSVKGRFTISRDAAKNMVYLQMNSLKSDDTAVYYCAVTIRGAATQ<br>TWKYDYWGRGTQVTVSSGSKTHTCPPCPAPELLGGPSVFLFPPKPKDTLMISRTPEVTCVVV<br>AVSHEDPEVKFNWYVDGVEVHNAKTKPREEQYASTYRVVSVLTVLHQDWLNGKEYKCKVSNK<br>ALPAPIEKTISKAKGQPREPQVYTLPPSRDELTKNQVSLTCLVKGFYPSDIAVEWESNGQPE<br>NNYKTTPPVLDSDGSFFLYSKLTVDKSRWQQGNVFSCSVMHEALHNHYTQKSLSLSPGKEFD<br>YKDDDDKLE
3xNb:TRAILR2(F5)-TNC-pCR3	MNFGFSLIFLVLVLKGVQCEVKLVPRGTQVQLVQSGGGLVQAGDSLRLSCAASGLTFPNYGM<br>GWFRQAPGEEREFLAWIYWSGGTVFYADSVKGRFTISRDAAKNMVYLQMNSLKSDDTAVYYC<br>AVTIRGAATQTWKYDYWGRGTQVTVSSRSGGGGSGGGGSGGGGSGGGGSGGGGSQVQLVQSG<br>GGLVQAGDSLRLSCAASGLTFPNYGMGWFRQAPGEEREFLAWIYWSGGTVFYADSVKGRFTI<br>SRDAAKNMVYLQMNSLKSDDTAVYYCAVTIRGAATQTWKYDYWGRGTQVTVSSGGGGSGGGG<br>SGGGGSGGGGSGGGGSQLQVQLVQSGGGLVQAGDSLRLSCAASGLTFPNYGMGWFRQAPGEE<br>REFLAWIYWSGGTVFYADSVKGRFTISRDAAKNMVYLQMNSLKSDDTAVYYCAVTIRGAATQ<br>TWKYDYWGRGTQVTVSSGSDYKDDDDKDIACGCAAAPDIKDLLSRLEELEGLVSSLREQGTG
3xNb:TRAILR2(F5)-GCN4-pCR3	MNFGFSLIFLVLVLKGVQCEVKLVPRGTQVQLVQSGGGLVQAGDSLRLSCAASGLTFPNYGM<br>GWFRQAPGEEREFLAWIYWSGGTVFYADSVKGRFTISRDAAKNMVYLQMNSLKSDDTAVYYC<br>AVTIRGAATQTWKYDYWGRGTQVTVSSRSGGGGSGGGGSGGGGSGGGGSGGGGSQVQLVQSG<br>GGLVQAGDSLRLSCAASGLTFPNYGMGWFRQAPGEEREFLAWIYWSGGTVFYADSVKGRFTI<br>SRDAAKNMVYLQMNSLKSDDTAVYYCAVTIRGAATQTWKYDYWGRGTQVTVSSGGGGSGGGG<br>SGGGGSGGGGSGGGGSQLQVQLVQSGGGLVQAGDSLRLSCAASGLTFPNYGMGWFRQAPGEE<br>REFLAWIYWSGGTVFYADSVKGRFTISRDAAKNMVYLQMNSLKSDDTAVYYCAVTIRGAATQ<br>TWKYDYWGRGTQVTVSSGSDYKDDDDKQLRLKQIEDKLEEILSKLYHIENELARIKKLLGER<br>EFGLE
Nb:TRAILR2(F5)-Fc-GpL-pCR3	MNFGFSLIFLVLVLKGVQCEVKLVPRQLQVQLVQSGGGLVQAGDSLRLSCAASGLTFPNYGM<br>GWFRQAPGEEREFLAWIYWSGGTVFYADSVKGRFTISRDAAKNMVYLQMNSLKSDDTAVYYC<br>AVTIRGAATQTWKYDYWGRGTQVTVSSGSKTHTCPPCPAPELLGGPSVFLFPPKPKDTLMIS<br>RTPEVTCVVVDVSHEDPEVKFNWYVDGVEVHNAKTKPREEQYNSTYRVVSVLTVLHQDWLNG<br>KEYKCKVSNKALPAPIEKTISKAKGQPREPQVYTLPPSRDELTKNQVSLTCLVKGFYPSDIA<br>VEWESNGQPENNYKTTPPVLDSDGSFFLYSKLTVDKSRWQQGNVFSCSVMHEALHNHYTQKS<br>LSLSPGKEFDYKDDDDKLEKPTENNEDFNIVAVASNFATTDLDADRGKLPGKKLPLEVLKEM<br>EANARKAGCTRGCLICLSHIKCTPKMKKFIPGRCHTYEGDKESAQGGIGEAIVDIPEIPGFK<br>DLEPMEQFIAQVDLCVDCTTGCLKGLANVQCSDLLKKWLPQRCATFASKIQGQVDKIKGAGG<br>D
3xNb:TNFR2(C188)-Fc(DANA)-pCR3	MNFGFSLIFLVLVLKGVQCEVKLVPRGTDVQLVESGGGSVQTGGSLTLSCAISGSTSERYCL<br>GWFRQAPGREREGVAATSLTGRGAQFYADSVKGRFTISLDDAKNTLYLQMDSLRPDDTAVYY<br>CAEDVGFLCGYDSDNDPFYDWGQGTQVTVSSRSGGGGSGGGGSGGGGSGGGGSGGGGSDVQL<br>VESGGGSVQTGGSLTLSCAISGSTSERYCLGWFRQAPGREREGVAATSLTGRGAQFYADSVK<br>GRFTISLDDAKNTLYLQMDSLRPDDTAVYYCAEDVGFLCGYDSDNDPFYDWGQGTQVTVSSG<br>GGGSGGGGSGGGGSGGGGSGGGGSQLDVQLVESGGGSVQTGGSLTLSCAISGSTSERYCLGW<br>FRQAPGREREGVAATSLTGRGAQFYADSVKGRFTISLDDAKNTLYLQMDSLRPDDTAVYYCA<br>EDVGFLCGYDSDNDPFYDWGQGTQVTVSSGASAGSKTHTCPPCPAPELLGGPSVFLFPPKPK

	DTLMISRTPEVTCVVVAVSHEDPEVKFNWYVDGVEVHNAKTKPREEQYASTYRVVSVLTVLH<br>QDWLNGKEYKCKVSNKALPAPIEKTISKAKGQPREPQVYTLPPSRDELTKNQVSLTCLVKGF<br>YPSDIAVEWESNGQPENNYKTTPPVLDSDGSFFLYSKLTVDKSRWQQGNVFSCSVMHEALHN<br>HYTQKSLSLSPGKEFDYKDDDDKLE
3xNb:TNFR2(C188)-TNC-pCR3	MNFGFSLIFLVLVLKGVQCEVKLVPRGTDVQLVESGGGSVQTGGSLTLSCAISGSTSERYCL<br>GWFRQAPGREREGVAATSLTGRGAQFYADSVKGRFTISLDDAKNTLYLQMDSLRPDDTAVYY<br>CAEDVGFLCGYDSDNDPFYDWGQGTQVTVSSRSGGGGSGGGGSGGGGSGGGGSGGGGSDVQL<br>VESGGGSVQTGGSLTLSCAISGSTSERYCLGWFRQAPGREREGVAATSLTGRGAQFYADSVK<br>GRFTISLDDAKNTLYLQMDSLRPDDTAVYYCAEDVGFLCGYDSDNDPFYDWGQGTQVTVSSG<br>GGGSGGGGSGGGGSGGGGSGGGGSQLDVQLVESGGGSVQTGGSLTLSCAISGSTSERYCLGW<br>FRQAPGREREGVAATSLTGRGAQFYADSVKGRFTISLDDAKNTLYLQMDSLRPDDTAVYYCA<br>EDVGFLCGYDSDNDPFYDWGQGTQVTVSSGASAGSDYKDDDDKDIACGCAAAPDIKDLLSRL<br>EELEGLVSSLREQGTG
3xNb:TNFR2(C188)-ALFA-GCN4-pCR3	MNFGFSLIFLVLVLKGVQCEVKLVPRGTDVQLVESGGGSVQTGGSLTLSCAISGSTSERYCL<br>GWFRQAPGREREGVAATSLTGRGAQFYADSVKGRFTISLDDAKNTLYLQMDSLRPDDTAVYY<br>CAEDVGFLCGYDSDNDPFYDWGQGTQVTVSSRSGGGGSGGGGSGGGGSGGGGSGGGGSDVQL<br>VESGGGSVQTGGSLTLSCAISGSTSERYCLGWFRQAPGREREGVAATSLTGRGAQFYADSVK<br>GRFTISLDDAKNTLYLQMDSLRPDDTAVYYCAEDVGFLCGYDSDNDPFYDWGQGTQVTVSSG<br>GGGSGGGGSGGGGSGGGGSGGGGSQLDVQLVESGGGSVQTGGSLTLSCAISGSTSERYCLGW<br>FRQAPGREREGVAATSLTGRGAQFYADSVKGRFTISLDDAKNTLYLQMDSLRPDDTAVYYWG<br>QGTQVTVSSGASAGSPSRLEEELRRRLTEPELRLKQIEDKLEEILSKLYHIENELARIKKLL<br>GEREFGLE
Nb:TNFR2(C188)-Fc-GpL-pCR3	MNFGFSLIFLVLVLKGVQCEVKLVPRQLDVQLVESGGGSVQTGGSLTLSCAISGSTSERYCL<br>GWFRQAPGREREGVAATSLTGRGAQFYADSVKGRFTISLDDAKNTLYLQMDSLRPDDTAVYY<br>CAEDVGFLCGYDSDNDPFYDWGQGTQVTVSSGASAGSKTHTCPPCPAPELLGGPSVFLFPPK<br>PKDTLMISRTPEVTCVVVDVSHEDPEVKFNWYVDGVEVHNAKTKPREEQYNSTYRVVSVLTV<br>LHQDWLNGKEYKCKVSNKALPAPIEKTISKAKGQPREPQVYTLPPSRDELTKNQVSLTCLVK<br>GFYPSDIAVEWESNGQPENNYKTTPPVLDSDGSFFLYSKLTVDKSRWQQGNVFSCSVMHEAL<br>HNHYTQKSLSLSPGKEFDYKDDDDKLEKPTENNEDFNIVAVASNFATTDLDADRGKLPGKKL<br>PLEVLKEMEANARKAGCTRGCLICLSHIKCTPKMKKFIPGRCHTYEGDKESAQGGIGEAIVD<br>IPEIPGFKDLEPMEQFIAQVDLCVDCTTGCLKGLANVQCSDLLKKWLPQRCATFASKIQGQV<br>DKIKGAGGD
3xNb:TNFR2(C238)-Fc(DANA)-pCR3	MNFGFSLIFLVLVLKGVQCEVKLVPRGTQVQLVESGGGSVQPGGSLTLSCVDSGSAYLSYCM<br>AWFRQAPGKEREGVAAIYYGGDSYYADSVKGRFTISQDHAKNTMYLQMNSLKPEDTAVYYCA<br>ADVGFICGYDSDNDPPNDWGQGTQVTVSSRSGGGGSGGGGSGGGGSGGGGSGGGGSQVQLVE<br>SGGGSVQPGGSLTLSCVDSGSAYLSYCMAWFRQAPGKEREGVAAIYYGGDSYYADSVKGRFT<br>ISQDHAKNTMYLQMNSLKPEDTAVYYCAADVGFICGYDSDNDPPNDWGQGTQVTVSSGGGGS<br>GGGGSGGGGSGGGGSGGGGSQLQVQLVESGGGSVQPGGSLTLSCVDSGSAYLSYCMAWFRQA<br>PGKEREGVAAIYYGGDSYYADSVKGRFTISQDHAKNTMYLQMNSLKPEDTAVYYCAADVGFI<br>CGYDSDNDPPNDWGQGTQVTVSSGASAGSKTHTCPPCPAPELLGGPSVFLFPPKPKDTLMIS<br>RTPEVTCVVVAVSHEDPEVKFNWYVDGVEVHNAKTKPREEQYASTYRVVSVLTVLHQDWLNG<br>KEYKCKVSNKALPAPIEKTISKAKGQPREPQVYTLPPSRDELTKNQVSLTCLVKGFYPSDIA<br>VEWESNGQPENNYKTTPPVLDSDGSFFLYSKLTVDKSRWQQGNVFSCSVMHEALHNHYTQKS<br>LSLSPGKEFDYKDDDDKLE
3xNb:TNFR2(C238)-TNC-pCR3	MNFGFSLIFLVLVLKGVQCEVKLVPRGTQVQLVESGGGSVQPGGSLTLSCVDSGSAYLSYCM<br>AWFRQAPGKEREGVAAIYYGGDSYYADSVKGRFTISQDHAKNTMYLQMNSLKPEDTAVYYCA<br>ADVGFICGYDSDNDPPNDWGQGTQVTVSSRSGGGGSGGGGSGGGGSGGGGSGGGGSQVQLVE<br>SGGGSVQPGGSLTLSCVDSGSAYLSYCMAWFRQAPGKEREGVAAIYYGGDSYYADSVKGRFT<br>ISQDHAKNTMYLQMNSLKPEDTAVYYCAADVGFICGYDSDNDPPNDWGQGTQVTVSSGGGGS<br>GGGGSGGGGSGGGGSGGGGSQLQVQLVESGGGSVQPGGSLTLSCVDSGSAYLSYCMAWFRQA<br>PGKEREGVAAIYYGGDSYYADSVKGRFTISQDHAKNTMYLQMNSLKPEDTAVYYCAADVGFI<br>CGYDSDNDPPNDWGQGTQVTVSSGASAGSDYKDDDDKDIACGCAAAPDIKDLLSRLEELEGL<br>VSSLREQGTG
3xNb:TNFR2(C238)-ALFA-GCN4-pCR3	MNFGFSLIFLVLVLKGVQCEVKLVPRGTQVQLVESGGGSVQPGGSLTLSCVDSGSAYLSYCM<br>AWFRQAPGKEREGVAAIYYGGDSYYADSVKGRFTISQDHAKNTMYLQMNSLKPEDTAVYYCA<br>ADVGFICGYDSDNDPPNDWGQGTQVTVSSRSGGGGSGGGGSGGGGSGGGGSGGGGSQVQLVE<br>SGGGSVQPGGSLTLSCVDSGSAYLSYCMAWFRQAPGKEREGVAAIYYGGDSYYADSVKGRFT<br>ISQDHAKNTMYLQMNSLKPEDTAVYYCAADVGFICGYDSDNDPPNDWGQGTQVTVSSGGGGS<br>GGGGSGGGGSGGGGSGGGGSQLQVQLVESGGGSVQPGGSLTLSCVDSGSAYLSYCMAWFRQA<br>PGKEREGVAAIYYGGDSYYADSVKGRFTISQDHAKNTMYLQMNSLKPEDTAVYYCAADVGFI<br>CGYDSDNDPPNDWGQGTQVTVSSGASAGSPSRLEEELRRRLTEPELRLKQIEDKLEEILSKL<br>YHIENELARIKKLLGEREFGLE
Nb:TNFR2(C238)-Fc-GpL-pCR3	MNFGFSLIFLVLVLKGVQCEVKLVPRQLQVQLVESGGGSVQPGGSLTLSCVDSGSAYLSYCM<br>AWFRQAPGKEREGVAAIYYGGDSYYADSVKGRFTISQDHAKNTMYLQMNSLKPEDTAVYYCA<br>ADVGFICGYDSDNDPPNDWGQGTQVTVSSGASAGSKTHTCPPCPAPELLGGPSVFLFPPKPK<br>DTLMISRTPEVTCVVVDVSHEDPEVKFNWYVDGVEVHNAKTKPREEQYNSTYRVVSVLTVLH<br>QDWLNGKEYKCKVSNKALPAPIEKTISKAKGQPREPQVYTLPPSRDELTKNQVSLTCLVKGF<br>YPSDIAVEWESNGQPENNYKTTPPVLDSDGSFFLYSKLTVDKSRWQQGNVFSCSVMHEALHN<br>HYTQKSLSLSPGKEFDYKDDDDKLEKPTENNEDFNIVAVASNFATTDLDADRGKLPGKKLPL<br>EVLKEMEANARKAGCTRGCLICLSHIKCTPKMKKFIPGRCHTYEGDKESAQGGIGEAIVDIP<br>EIPGFKDLEPMEQFIAQVDLCVDCTTGCLKGLANVQCSDLLKKWLPQRCATFASKIQGQVDK<br>IKGAGGD
3xNb:CD40(1B6)-ALFA-Fc(DANA)-pCR3	MNFGFSLIFLVLVLKGVQCEVKLVPRGTEVQLQESGGGLVQPGGSLRLSCVASGLHFDAAVM<br>SWVRQAPGKGMEWVSSIYSYDRKTYYAASVRGRFTLSTNNAKNTMYLQMDNLKAEDTAIYYC<br>AGDGAVAGSANRDDYQYWGQGTQVTVSSRSGGGGSGGGGSGGGGSGGGGSGGGGSEVQLQES<br>GGGLVQPGGSLRLSCVASGLHFDAAVMSWVRQAPGKGMEWVSSIYSYDRKTYYAASVRGRFT<br>LSTNNAKNTMYLQMDNLKAEDTAIYYCAGDGAVAGSANRDDYQYWGQGTQVTVSSGGGGSGG

	GGSGGGGSGGGGSGGGGSQFEVQLQESGGGLVQPGGSLRLSCVASGLHFDAAVMSWVRQAPG<br>KGMEWVSSIYSYDRKTYYAASVRGRFTLSTNNAKNTMYLQMDNLKAEDTAIYYCAGDGAVAG<br>SANRDDYQYWGQGTQVTVSSGSPSRLEEELRRRLTEPEFKTHTCPPCPAPELLGGPSVFLFP<br>PKPKDTLMISRTPEVTCVVVAVSHEDPEVKFNWYVDGVEVHNAKTKPREEQYASTYRVVSVL<br>TVLHQDWLNGKEYKCKVSNKALPAPIEKTISKAKGQPREPQVYTLPPSRDELTKNQVSLTCL<br>VKGFYPSDIAVEWESNGQPENNYKTTPPVLDSDGSFFLYSKLTVDKSRWQQGNVFSCSVMHE<br>ALHNHYTQKSLSLSPGKLE
3xNb:CD40(1B6)-ALFA-TNC-pCR3	MNFGFSLIFLVLVLKGVQCEVKLVPRGTEVQLQESGGGLVQPGGSLRLSCVASGLHFDAAVM<br>SWVRQAPGKGMEWVSSIYSYDRKTYYAASVRGRFTLSTNNAKNTMYLQMDNLKAEDTAIYYC<br>AGDGAVAGSANRDDYQYWGQGTQVTVSSRSGGGGSGGGGSGGGGSGGGGSGGGGSEVQLQES<br>GGGLVQPGGSLRLSCVASGLHFDAAVMSWVRQAPGKGMEWVSSIYSYDRKTYYAASVRGRFT<br>LSTNNAKNTMYLQMDNLKAEDTAIYYCAGDGAVAGSANRDDYQYWGQGTQVTVSSGGGGSGG<br>GGSGGGGSGGGGSGGGGSQFEVQLQESGGGLVQPGGSLRLSCVASGLHFDAAVMSWVRQAPG<br>KGMEWVSSIYSYDRKTYYAASVRGRFTLSTNNAKNTMYLQMDNLKAEDTAIYYCAGDGAVAG<br>SANRDDYQYWGQGTQVTVSSGSPSRLEEELRRRLTEPEFDIACGCAAAPDIKDLLSRLEELE<br>GLVSSLREQGTGLE
3xNb:CD40(1B6)-ALFA-GCN4-pCR3	MNFGFSLIFLVLVLKGVQCEVKLVPRGTEVQLQESGGGLVQPGGSLRLSCVASGLHFDAAVM<br>SWVRQAPGKGMEWVSSIYSYDRKTYYAASVRGRFTLSTNNAKNTMYLQMDNLKAEDTAIYYC<br>AGDGAVAGSANRDDYQYWGQGTQVTVSSRSGGGGSGGGGSGGGGSGGGGSGGGGSEVQLQES<br>GGGLVQPGGSLRLSCVASGLHFDAAVMSWVRQAPGKGMEWVSSIYSYDRKTYYAASVRGRFT<br>LSTNNAKNTMYLQMDNLKAEDTAIYYCAGDGAVAGSANRDDYQYWGQGTQVTVSSGGGGSGG<br>GGSGGGGSGGGGSGGGGSQFEVQLQESGGGLVQPGGSLRLSCVASGLHFDAAVMSWVRQAPG<br>KGMEWVSSIYSYDRKTYYAASVRGRFTLSTNNAKNTMYLQMDNLKAEDTAIYYCAGDGAVAG<br>SANRDDYQYWGQGTQVTVSSGSPSRLEEELRRRLTEPELRLKQIEDKLEEILSKLYHIENEL<br>ARIKKLLGEREFGLE
Nb:CD40(1B6)-Fc-GpL-pCR3	MNFGFSLIFLVLVLKGVQCEVKLVPRQFEVQLQESGGGLVQPGGSLRLSCVASGLHFDAAVM<br>SWVRQAPGKGMEWVSSIYSYDRKTYYAASVRGRFTLSTNNAKNTMYLQMDNLKAEDTAIYYC<br>AGDGAVAGSANRDDYQYWGQGTQVTVSSGSKTHTCPPCPAPELLGGPSVFLFPPKPKDTLMI<br>SRTPEVTCVVVDVSHEDPEVKFNWYVDGVEVHNAKTKPREEQYNSTYRVVSVLTVLHQDWLN<br>GKEYKCKVSNKALPAPIEKTISKAKGQPREPQVYTLPPSRDELTKNQVSLTCLVKGFYPSDI<br>AVEWESNGQPENNYKTTPPVLDSDGSFFLYSKLTVDKSRWQQGNVFSCSVMHEALHNHYTQK<br>SLSLSPGKEFDYKDDDDKLEKPTENNEDFNIVAVASNFATTDLDADRGKLPGKKLPLEVLKE<br>MEANARKAGCTRGCLICLSHIKCTPKMKKFIPGRCHTYEGDKESAQGGIGEAIVDIPEIPGF<br>KDLEPMEQFIAQVDLCVDCTTGCLKGLANVQCSDLLKKWLPQRCATFASKIQGQVDKIKGAG<br>GD

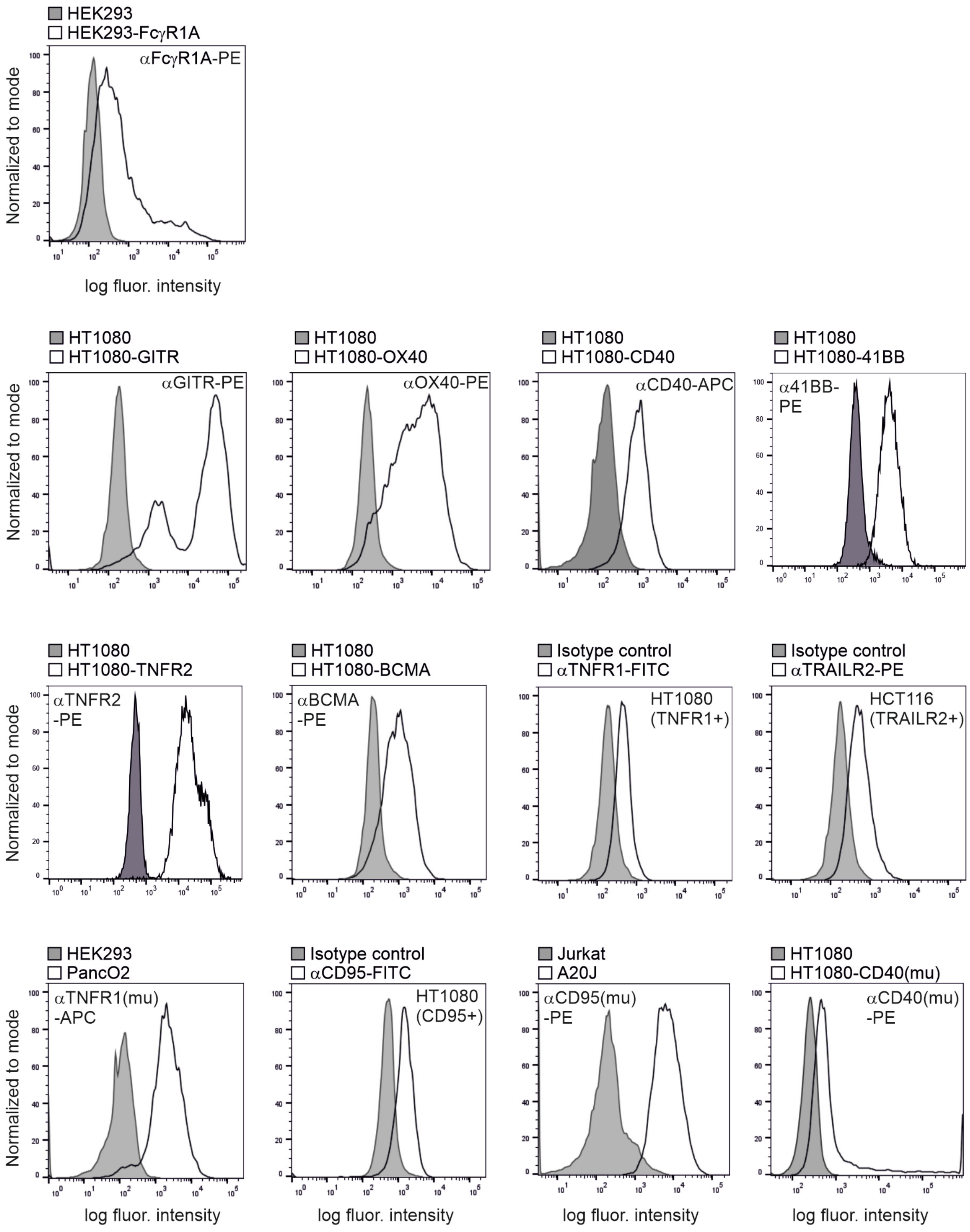

Supplemental Figure S1. HEK293 cells transiently transfected with empty vector or an expression plasmid encoding FcγRIA and the various TNFR responder cell lines used in this study were analyzed for cell surface expression of the indicated molecules by flow cytometry.

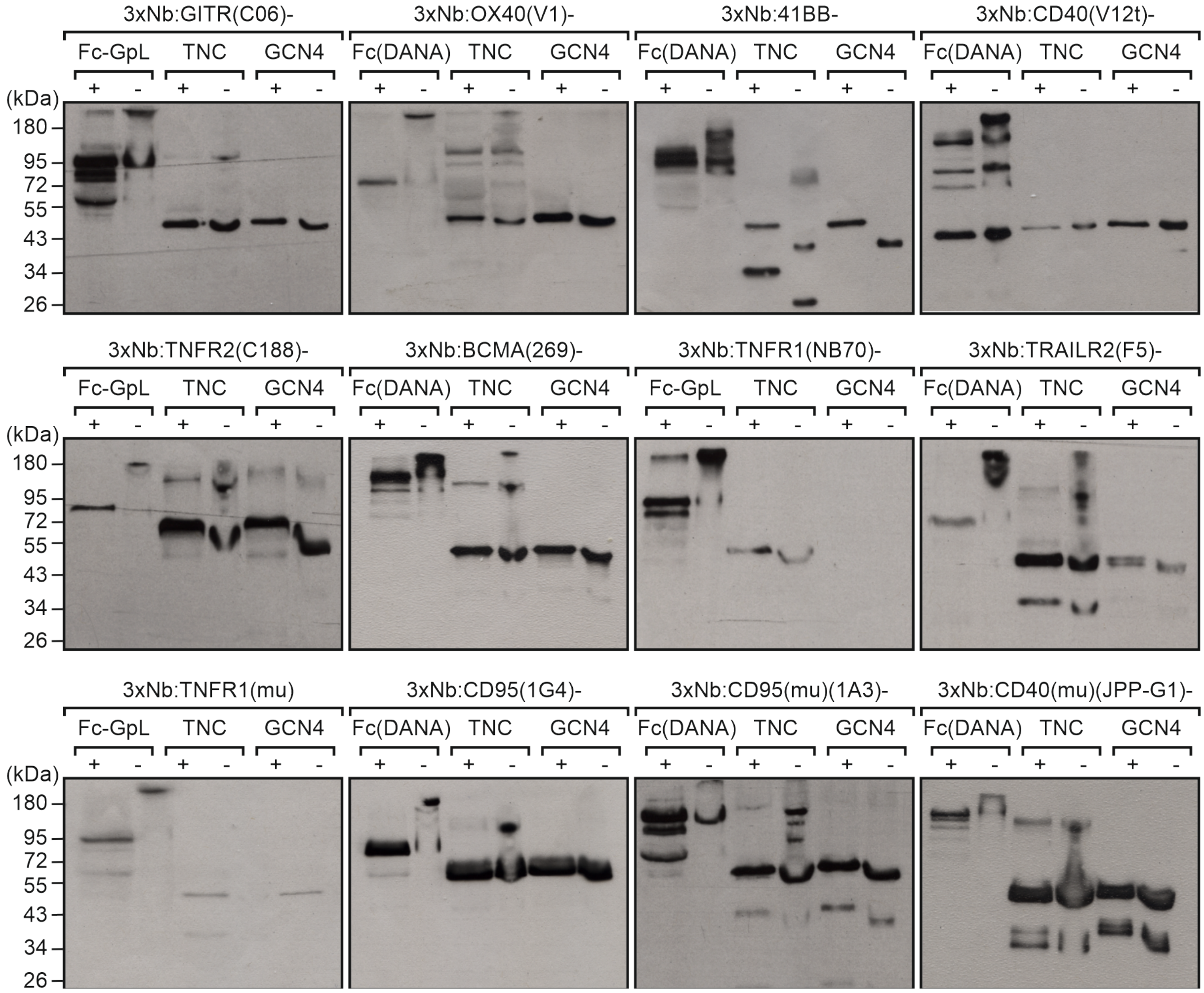

Supplemental Figure S2. Cell culture supernatants of HEK293 cells transiently expressing the indicated 3xNb:TNFR fusion proteins were denatured with conventional Lämmli sample buffer (containing ß-mercaptoethanol; +) and non-reducing Laemmli sample buffer (w/o ß-mercaptoethanol; -), resolved by SDS-PAGE and analyzed by western blotting using an Direct-Blot™ HRP anti-Flag Tag antibody and HRP conjugated sdAb anti-ALFA.

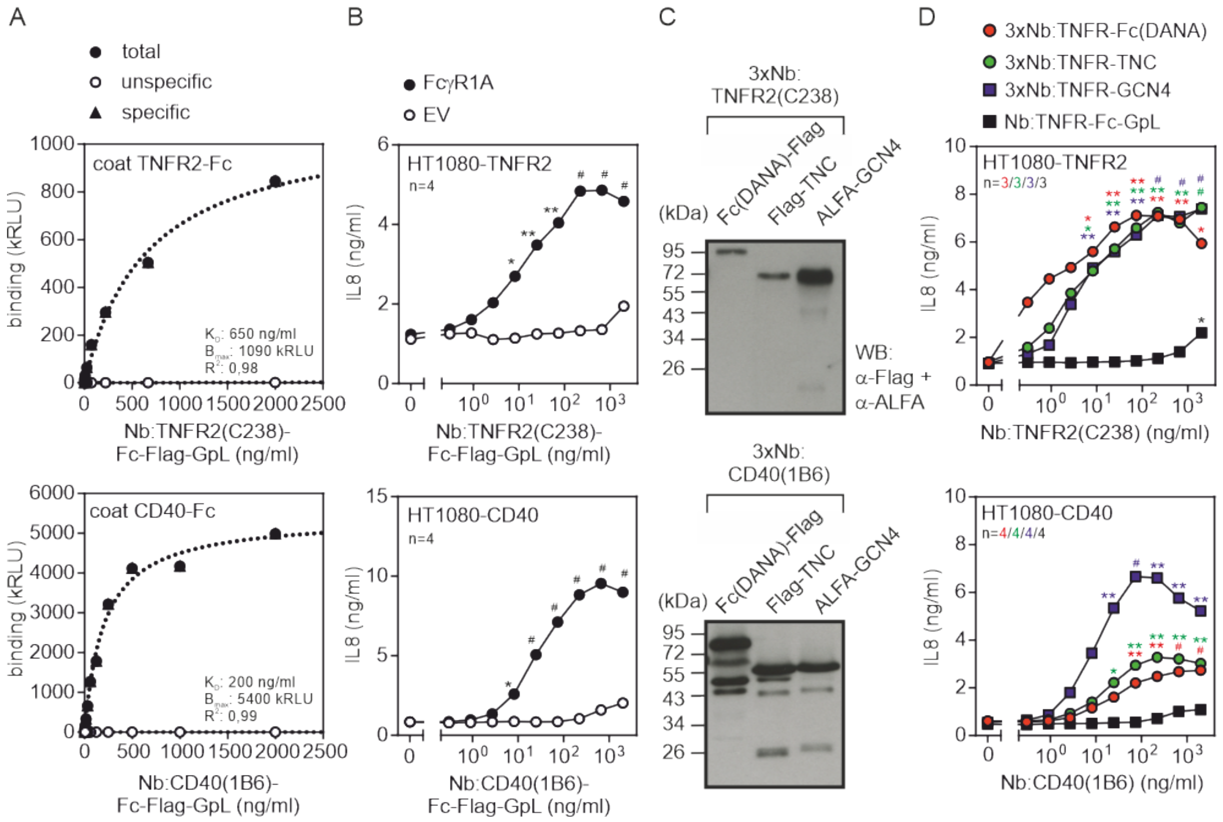

Supplemental Figure S3. Characterization of oligovalent constructs derived of nanobody clones C238 (TNFR2) and 1B6 (CD40). (A) Binding of Nb:TNFR2(238)-Fc-GpL and Nb:CD40(1B6)-Fc-GpL to plastic-immobilized TNFR(ed)-Fc fusion proteins. (B) The indicated cells (10^4 per well) responding with IL8 production to TNFR2 and CD40 stimulation were cultured in 96-well plates overnight and supplemented next day (1:1) with HEK293 cells transfected with empty vector (EV) or an expression plasmid encoding FcγR1A along with increasing concentrations of the indicated bivalent Nb:TNFR(ed)-Fc-GpL constructs. The following day, the amount of IL8 in the cell culture supernatants was determined. Upregulation of IL8 production served as a readout for TNFR activation. (C) WB of supernatants of cells producing TNFR(ed)-specific oligovalent Nb fusion proteins. (D) The indicated cell lines were challenged overnight with cell culture supernatants containing the indicated oligovalent Nb:TNFR fusion proteins. Next day, cells were analyzed for IL8 production by ELISA. Shown are averaged data and the number of independent experiments is noted in the corresponding graph. Data were analyzed using the multiple unpaired t-test. * $p < 0.05$; ** $p < 0.01$; # $p < 0.0001$.

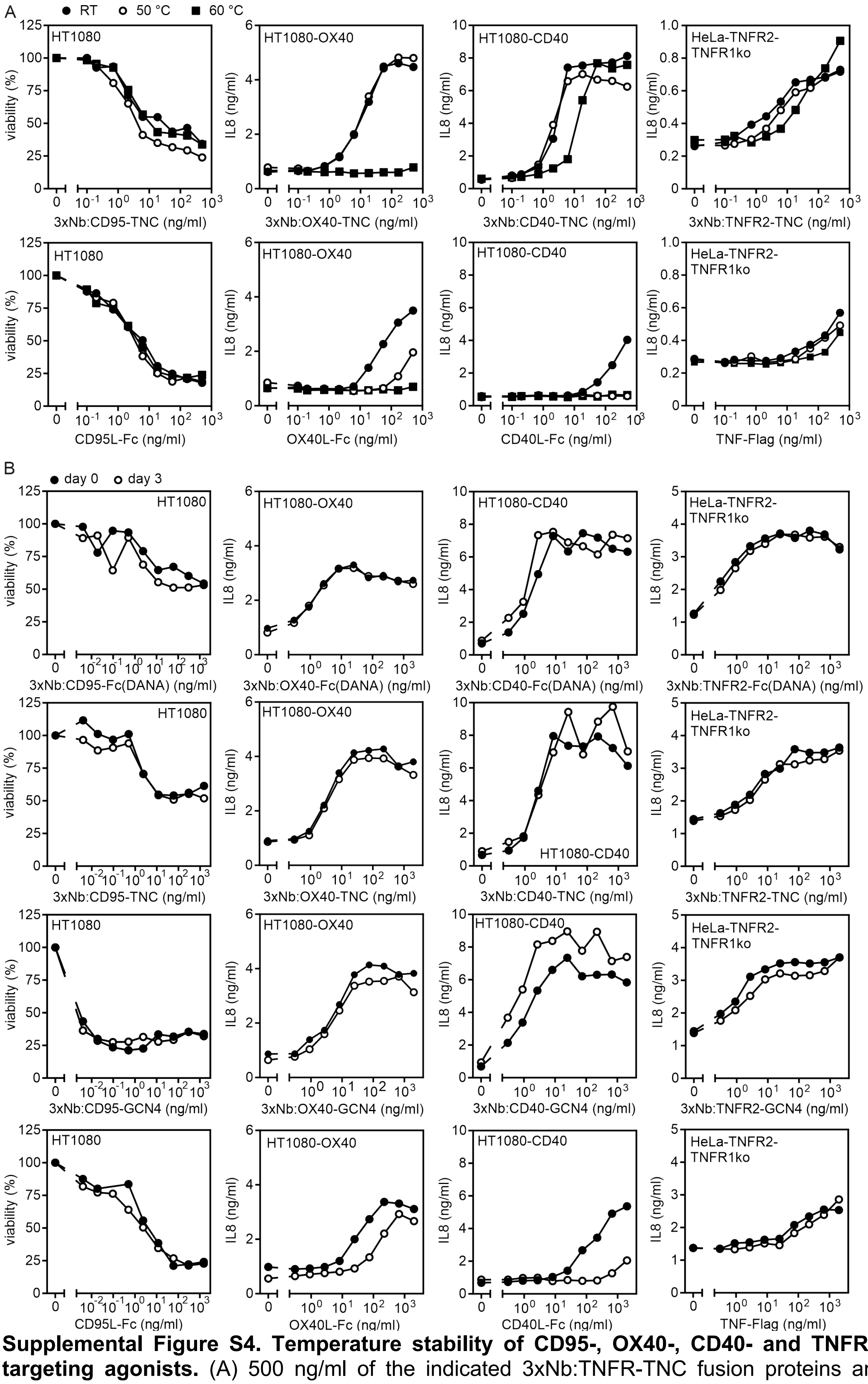

Supplemental Figure S4. Temperature stability of CD95-, OX40-, CD40- and TNFR2-targeting agonists. (A) 500 ng/ml of the indicated 3xNb:TNFR-TNC fusion proteins and

corresponding TNFL constructs were incubated at 50 or 60°C for 60 min or stayed at room temperature (RT). Afterwards, the fusion proteins and TNFL constructs were titrated and used to stimulate the indicated cell lines overnight. Then, cells were analyzed for IL8 production by ELISA or cellular viability. HT1080 cells were sensitized for cell death induction with 2.5 µg/ml CHX. Results of one of two similar experiments is shown. (B) Hexa-, nona- and dodecavalent 3xNb:TNFR fusion proteins and corresponding TNFLs were titrated and split. One dilution series was incubated for three days at 37°C (open circles) and the second one (filled circles) was stored at +4°C. Indicated cell lines were treated side-by-side with the two dilution series and analyzed Next day, cells were analyzed for cell death induction (HT1080) or IL8 production by ELISA. Results of one of three similar experiments experiment is shown.

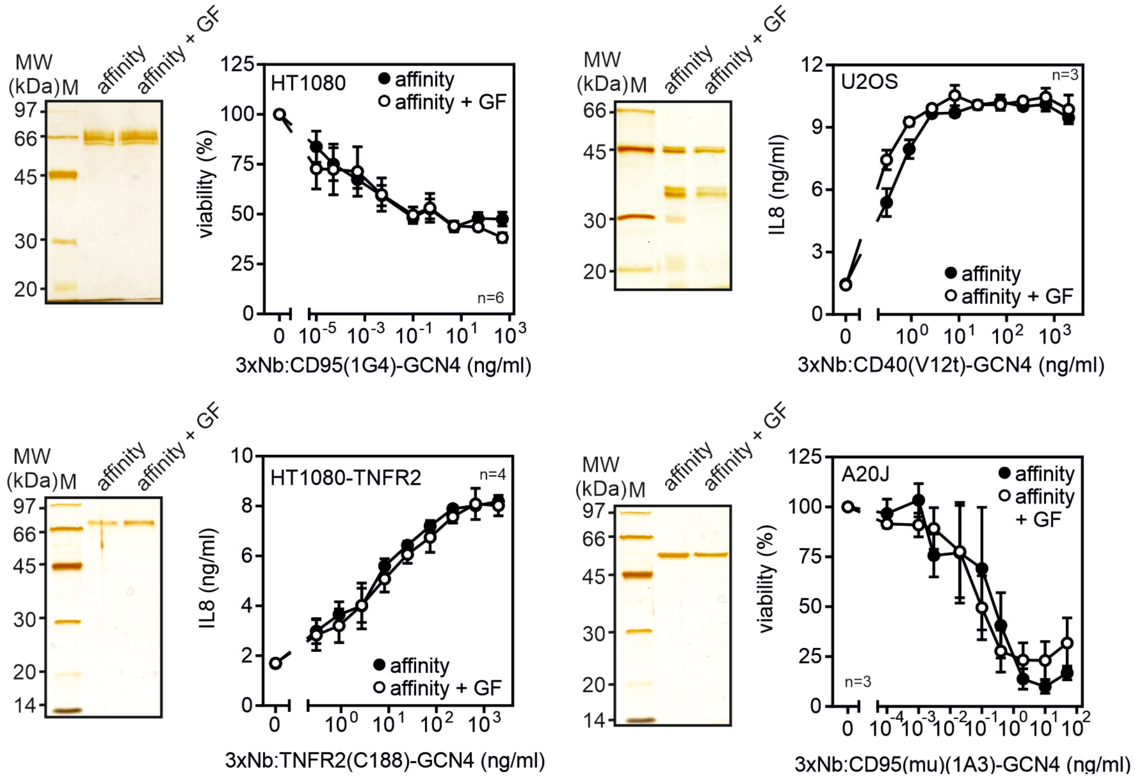

Supplemental Figure S5. Comparison of affinity- versus affinity plus gel filtration (GF) purified 3xNb:TNFR fusion proteins. Affinity purified 3xNb:TNFR-GCN4 fusion proteins before and after preparative size exclusion chromatography (peak fractions) were analyzed by SDS-PAGE analysis and silver staining. Then, both purified protein samples (affinity-purified vs. affinity-purified plus gel filtrated (affinity + GF)) were analyzed side by side regarding their TNFR-stimulating potential. Therefore, cells were treated overnight with the indicated concentrations of the various samples. Cells were finally analyzed for IL8 production by ELISA or for viability via crystal violet or MTT staining. HT1080 and A20J cells were sensitized for cell death induction with 2.5 µg/ml CHX. Data shown are derived of four independent experiments (mean ± SEM).

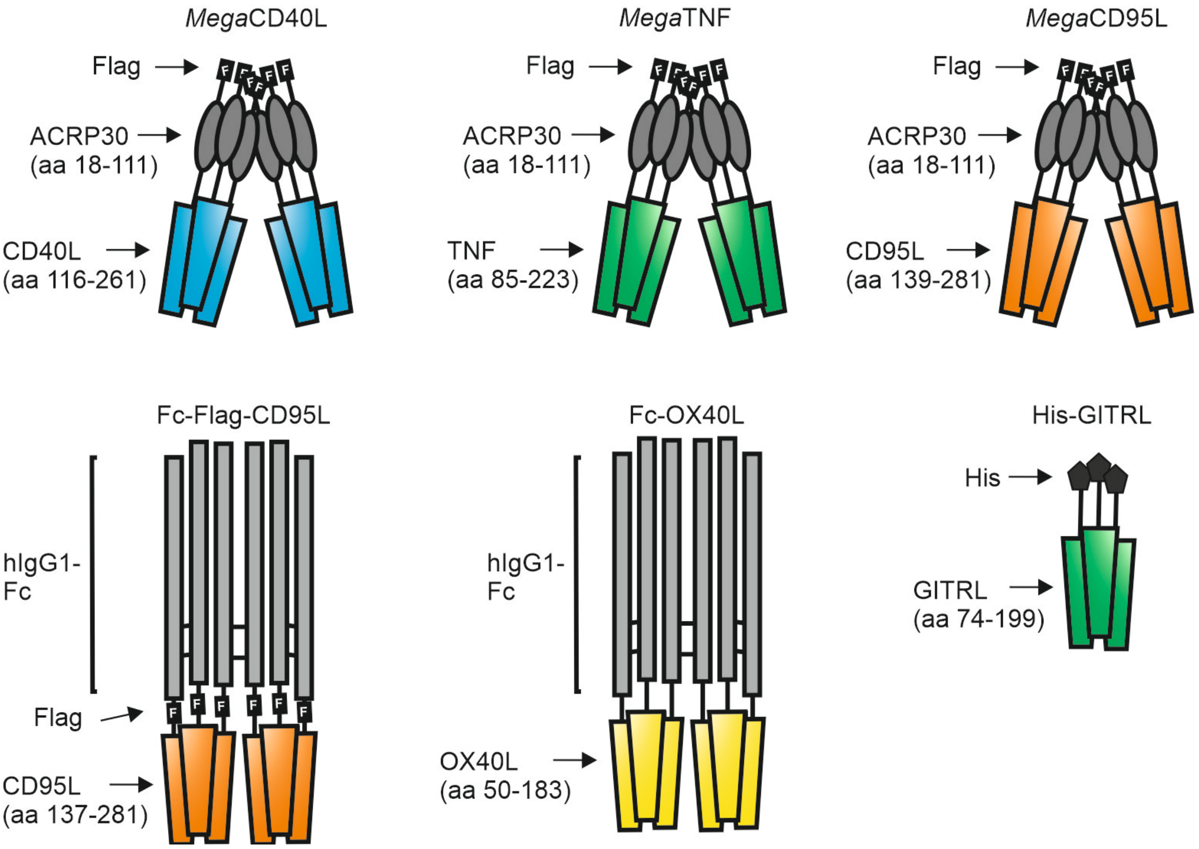

Supplemental Figure S6. Domain architecture of the TNFL-based TNFR agonists used in this study as benchmarks for the oligovalent Nb:TNFR agonists.
