# Supplementary material for: A simple and broadly applicable nanobody-based approach to generate potent TNFR agonists
Source: Cell Death Dis. 2026 May 30;17(1):518. doi: 10.1038/s41419-026-08911-x (PMC13222343; doi:10.1038/s41419-026-08911-x)

ORIGINAL DATA FILE

A simple and broadly applicable nanobody-based approach to generate potent TNFR agonists

Isabell Lang[1], Olena Zaitseva[1], Amelie Glöckler[1], Daniela Siegmund[1], Dalia Sheta[2], Bayan Mouhandes[1], Daniela Weisenberger[1], Viktoria Schäfer[1], Svetlana Stepanzow[1], Theresa Schneider[2], Andreas Beilhack[2], Alexander Crauel[3], Markus Kilisch[3], Lisa-Marie Funk[3], Hansjörg Götzke[3], Steffen Frey[3] and Harald Wajant[1]

Figure 1B

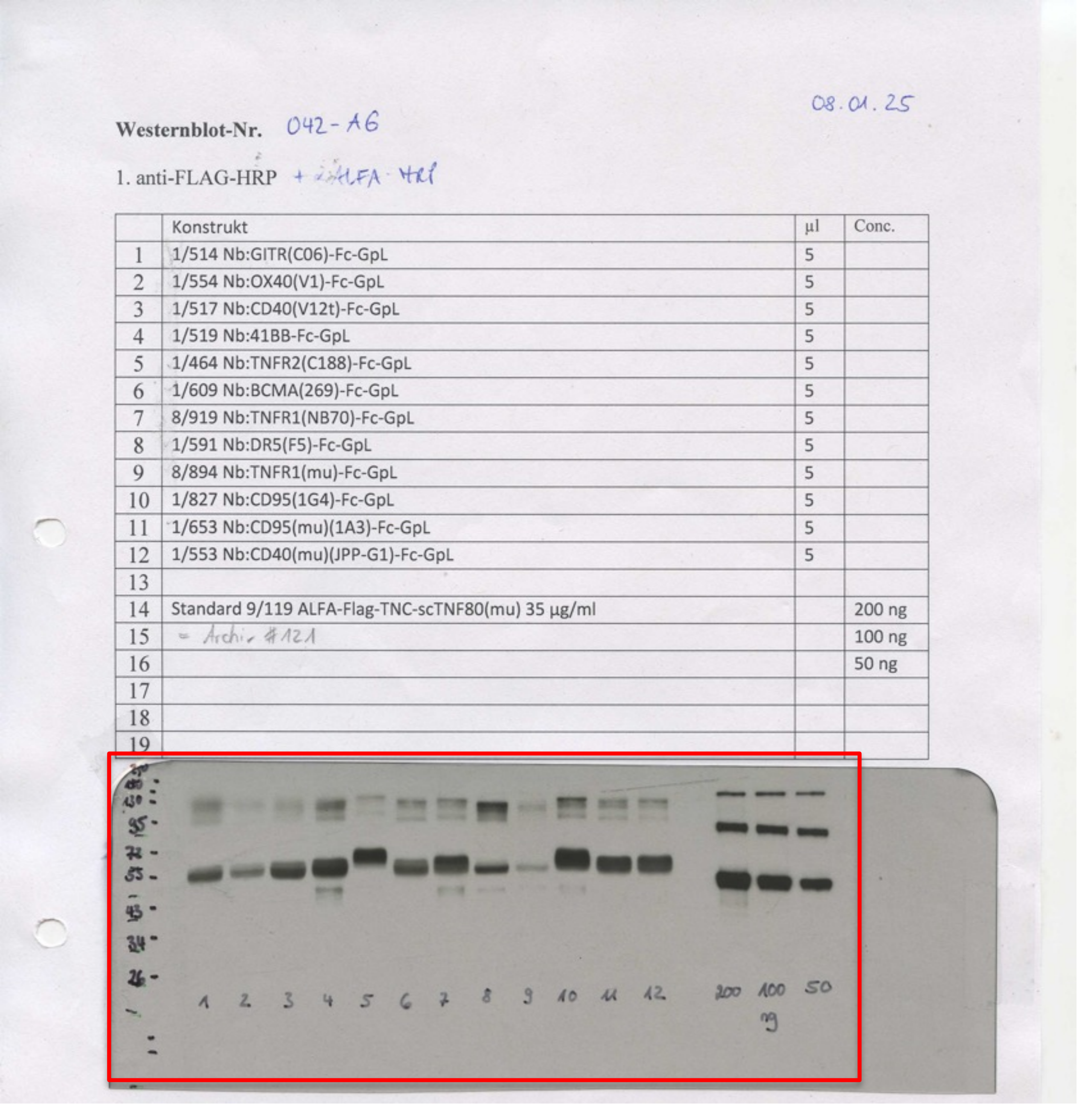

Westernblot-Nr. 042-A6 08.01.25

1. anti-FLAG-HRP + [illegible]ALFA-HRP

	Konstrukt	µl	Conc.
1	1/514 Nb:GITR(C06)-Fc-GpL	5	
2	1/554 Nb:OX40(V1)-Fc-GpL	5	
3	1/517 Nb:CD40(V12t)-Fc-GpL	5	
4	1/519 Nb:41BB-Fc-GpL	5	
5	1/464 Nb:TNFR2(C188)-Fc-GpL	5	
6	1/609 Nb:BCMA(269)-Fc-GpL	5	
7	8/919 Nb:TNFR1(NB70)-Fc-GpL	5	
8	1/591 Nb:DR5(F5)-Fc-GpL	5	
9	8/894 Nb:TNFR1(mu)-Fc-GpL	5	
10	1/827 Nb:CD95(1G4)-Fc-GpL	5	
11	1/653 Nb:CD95(mu)(1A3)-Fc-GpL	5	
12	1/553 Nb:CD40(mu)(JPP-G1)-Fc-GpL	5	
13			
14	Standard 9/119 ALFA-Flag-TNC-scTNF80(mu) 35 µg/ml		200 ng
15	= Archiv #121		100 ng
16			50 ng
17			
18			
19			

Figure 3 B

Westernblot-Nr. 049 AG **Datum 26.03.2025**

1. Anti-FLAG-HRP (1:50 000) + Anti-ALFA-HRP (1:75 000)

	Konstrukt		Volume µl	Menge
1	9/239	anti-TNFR1(NB70)-VHH(3x)-Fc-Flag-Gaussia(w/o)-pCR3	20 µl	
2	9/496	anti-TNFR1(NB70)-VHH(3x)-Flag-TNC-pCR3	20 µl	
3	9/511	anti-TNFR1(NB70)-VHH(3x)-ALFAtag-GCN4-pCR3	20 µl	
4				
5	9/111	anti-DR5(F5)-VHH(3x)-Fc(DANA)-Flag-pCR3	20 µl	
6	9/127	anti-DR5(F5)-VHH(3x)-TNC Flag-pCR3	20 µl	
7	9/539	anti-DR5(F5)-VHH(3x)-ALFAtag-GCN4-pCR3	20 µl	
8				
9	9/270	anti-TNFR1(mu)-VHH(3x)-Fc-Flag-Gaussia(w/o)-pCR3 Amelie	20 µl	
10	9/507	anti-TNFR1(mu)-VHH(3x)-Flag-TNC-pCR3	20 µl	
11	9/512	anti-TNFR1(mu)-VHH(3x)-ALFAtag-GCN4-pCR3	20 µl	
12				
13	9/270	anti-TNFR1(mu)-VHH(3x)-Fc-Flag-Gaussia(w/o)-pCR3 Svetlana	20 µl	
14	9/507	anti-TNFR1(mu)-VHH(3x)-Flag-TNC-pCR3	20 µl	
15	9/512	anti-TNFR1(mu)-VHH(3x)-ALFAtag-GCN4-pCR3	20 µl	
16				
17	Standard 9/119 ALFAtag-Flag-TNC-scTNF80(mu)-pCR3 35 µg/ml			200 ng
18				100 ng
19				50 ng

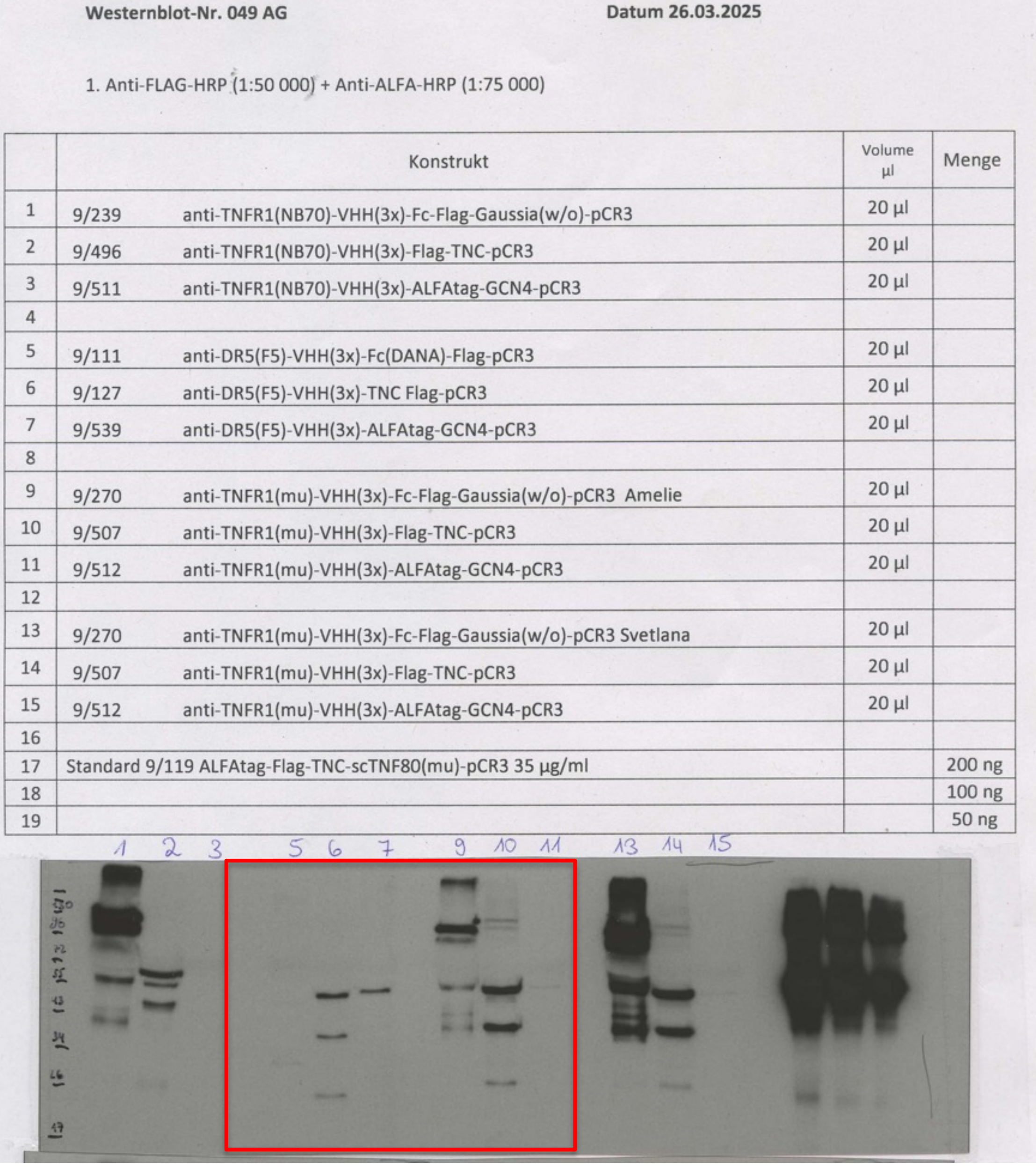

Westernblot-Nr. 043-AG 08.01.25

1. anti-FLAG-HRP + anti-ALFA-HRP

	Konstrukt	µl	Conc.
1	8/841 3xNb:GITR(C06)-Fc-Flag-GpL	10	
2	9/279 3xNb:GITR(C06)-Flag-TNC	10	
3	9/505 3xNb:GITR(C06)-ALFA-GCN4	10	
4			
5	8/922 3xNb:OX40(V1)-Fc(DANA)-Flag	10	
6	9/277 3xNb:OX40(V1)-Flag-TNC	10	
7	9/504 3xNb:OX40(V1)-ALFA-GCN4	10	
8			
9	9/454 3xNb:CD40(V12t)-ALFA-Fc(DANA)	10	
10	9/441 3xNb:CD40(V12t)-ALFA-TNC	10	
11	9/452 3xNb:CD40(V12t)-ALFA-GCN4	10	
12			
13	8/819 3xNb:41BB-Fc(DANA)-Flag	10	
14	9/278 3xNb:41BB-Flag-TNC	10	
15	9/427 GCN4-Flag-3xNb:41BB	10	
16			
17	Standard 9/119 ALFA-Flag-TNC-scTNF80(mu) 35 µg/ml		200 ng
18			100 ng
19			50 ng

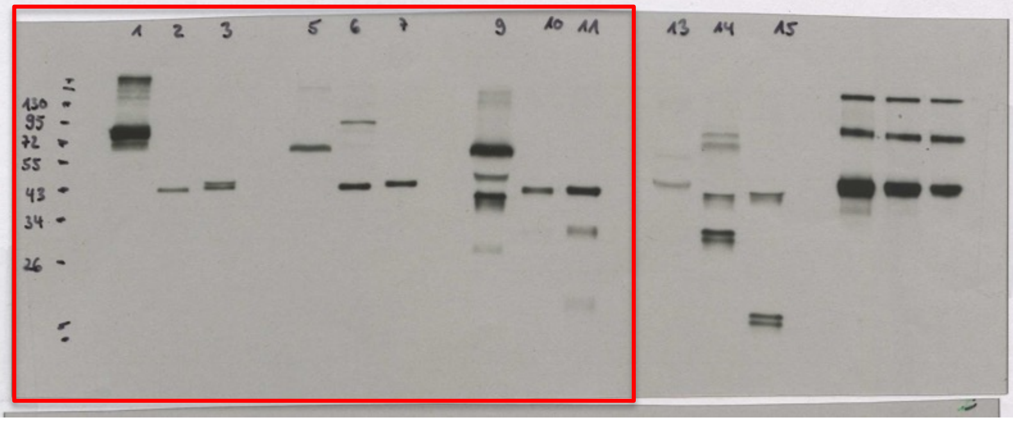

Westernblot-Nr. 044 - AG 08.01.25

1. anti-FLAG-HRP + anti-ALFA-HRP

	Konstrukt	µl	Conc.
1	8/800 3xNb:TNFR2(C188)-Fc(DANA)-Flag	10	
2	8/849 3xNb:TNFR2(C188)-Flag-TNC	10	
3	9/563 3xNb: TNFR2(C188)-ALFA-GCN4	10	
4			
5	9/113 3xNb:BCMA(269)-Fc(DANA)-Flag	10	
6	9/125 3xNb:BCMA(269)-Flag-TNC	10	
7	9/510 3xNb:BCMA(269)-ALFA-GCN4	10	
8			
9	9/239 3xNb:TNFR1(NB70)-Fc-Flag-GpL	10	
10	9/496 3xNb:TNFR1(NB70)-Flag-TNC	10	
11	9/511 3xNb:TNFR1(NB70)- ALFA-GCN4	10	
12			
13	9/111 3xNb:DR5(F5)-Fc(DANA)-Flag	10	
14	9/127 3xNb: DR5(F5)-Flag-TNC	10	
15	9/5~~39~~ 3xNb: DR5(F5)-~~ALFA~~-GCN4	10	
16	64 Flag		
17	Standard 9/119 ALFA-Flag-TNC-scTNF80(mu) 35 µg/ml		200 ng
18	= Archiv # 121		100 ng
19			50 ng

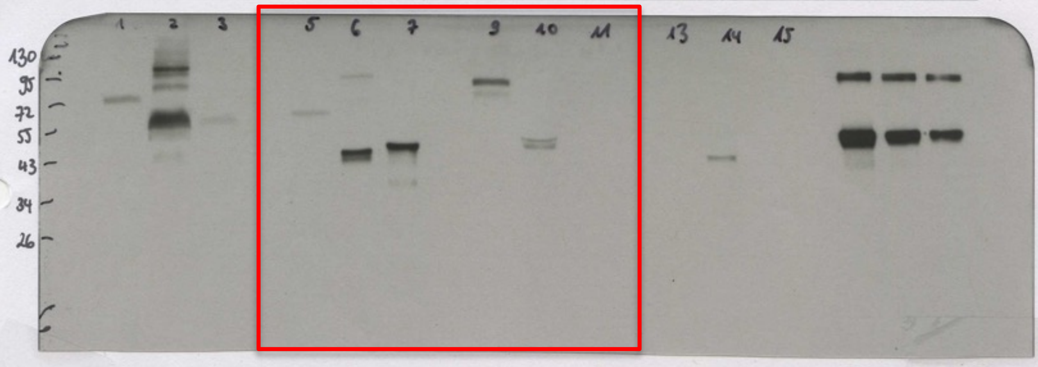

Westernblot-Nr. 045 -AG 08.01.25

1. anti-FLAG-HRP + anti-ALFA-HRP

	Konstrukt	µl	Conc.
1	9/270 3xNb:TNFR1(mu)-Fc-Flag-GpL	10	
2	9/507 3xNb:TNFR1(mu)-Flag-TNC	10	
3	9/512 3xNb:TNFR1(mu)-ALFA-GCN4	10	
4			
5	9/544 3xNb:CD95(1G4)-ALFA-Fc(DANA)	10	
6	9/538 3xNb:CD95(1G4)-ALFA-TNC	10	
7	9/537 3xNb: CD95(1G4)-ALFA-GCN4	10	
8			
9	9/248 3xNb:CD95(mu)(1A3)-Fc-Flag-GpL	10	
10	9/494 3xNb:CD95(mu)(1A3)-ALFA-TNC	10	
11	9/495 3xNb:CD95(mu)(1A3)-ALFA-GCN4	10	
12			
13	8/917 3xNb:CD40(mu)(JPP-G1)-Fc(DANA)-Flag	10	
14	8/944 3xNb:CD40(mu)(JPP-G1)-Flag-TNC	10	
15	9/509 3xNb:CD40(mu)(JPP-G1)-ALFA-GCN4	10	
16			
17	Standard 9/119 ALFA-Flag-TNC-scTNF80(mu) 35 µg/ml		200 ng
18			100 ng
19			50 ng

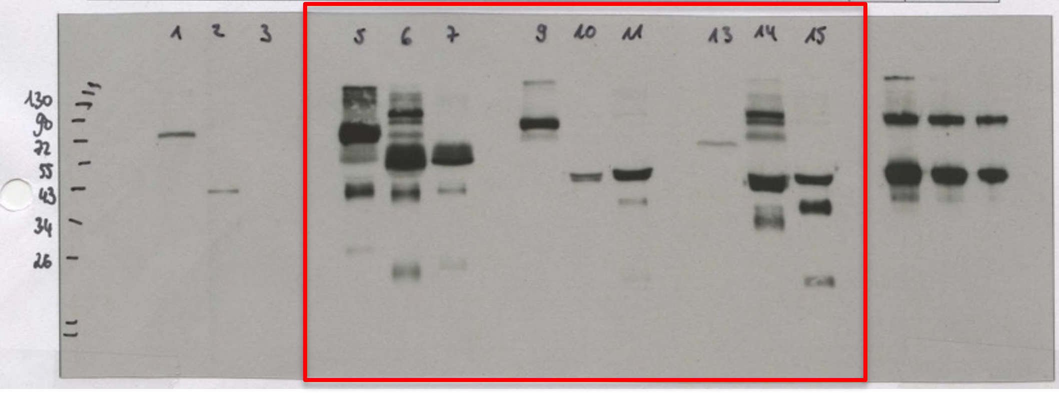

Westernblot-Nr. 048 AG **Datum 26.03.2025**

1. Anti-FLAG-HRP (1:50 000) + Anti-ALFA-HRP (1:75 000)

	Konstrukt		Volume µl	Menge
1	8/819	anti-41BB-VHH(3x)-Fc(DANA)-Flag-pCR3	15 µl	
2	9/278	anti-41BB-VHH(3x)-Flag-TNC-pCR3	15 µl	
3	9/427	anti-41BB-VHH(3x)-GCN4-Flag-pCR3	15 µl	
4				
5	8/800	C188-VHH(3x)-Fc(DANA)-Flag-pCR3 (neu)	20 µl	
6	8/849	C188-VHH(3x)-Flag-TNC-pCR3	20 µl	
7	9/563	C188-VHH(3x)-ALFAtag-GCN4-pCR3	20 µl	
8				
9	9/113	anti-BCMA(269A37948)-VHH(3x)-Fc(DANA)-Flag-pCR3	15 µl	
10	9/125	anti-BCMA(269A37948)-VHH(3x)-Flag-TNC-pCR3	15 µl	
11	9/510	anti-BCMA(269A37948)-VHH(3x)-ALFAtag-GCN4-pCR3	15 µl	
12				
13	9/544	anti-CD95(1G4-63-92)-VHH(3x)-ALFAtag-Fc(DANA)-pCR3	5 µl	
14	9/538	anti-CD95(1G4-63-92)-VHH(3x)-ALFAtag-TNC-pCR3	5 µl	
15	9/537	anti-CD95(1G4-63-92)-VHH(3x)-ALFAtag-GCN4-pCR3	5 µl	
16				
17	Standard 9/119 ALFAtag-Flag-TNC-scTNF80(mu)-pCR3 35 µg/ml			200 ng
18				100 ng
19				50 ng

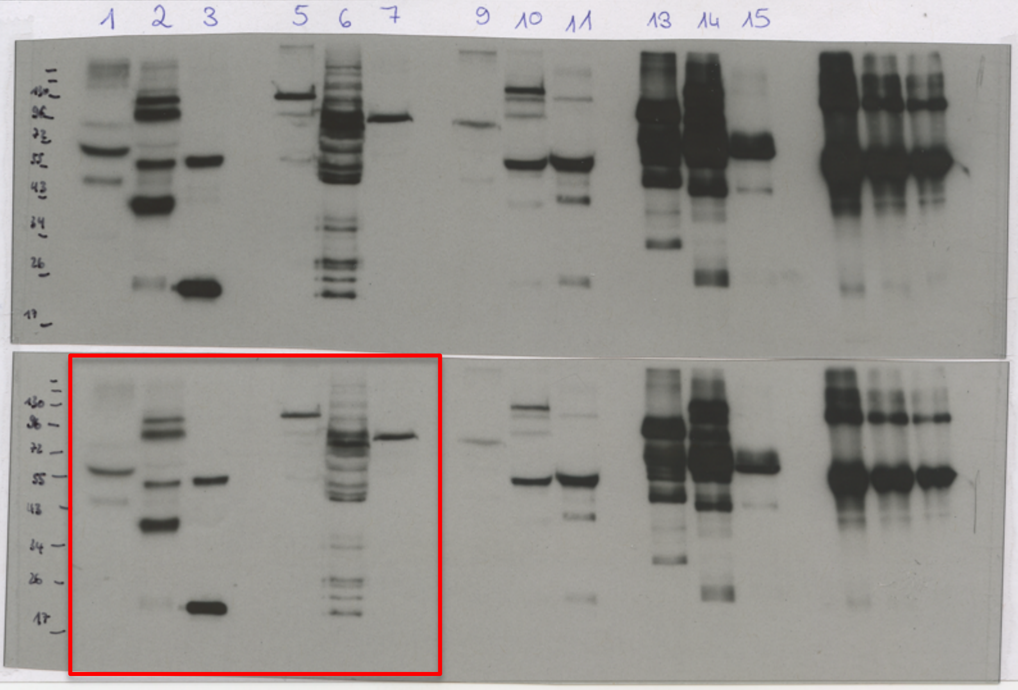

Figure 5D

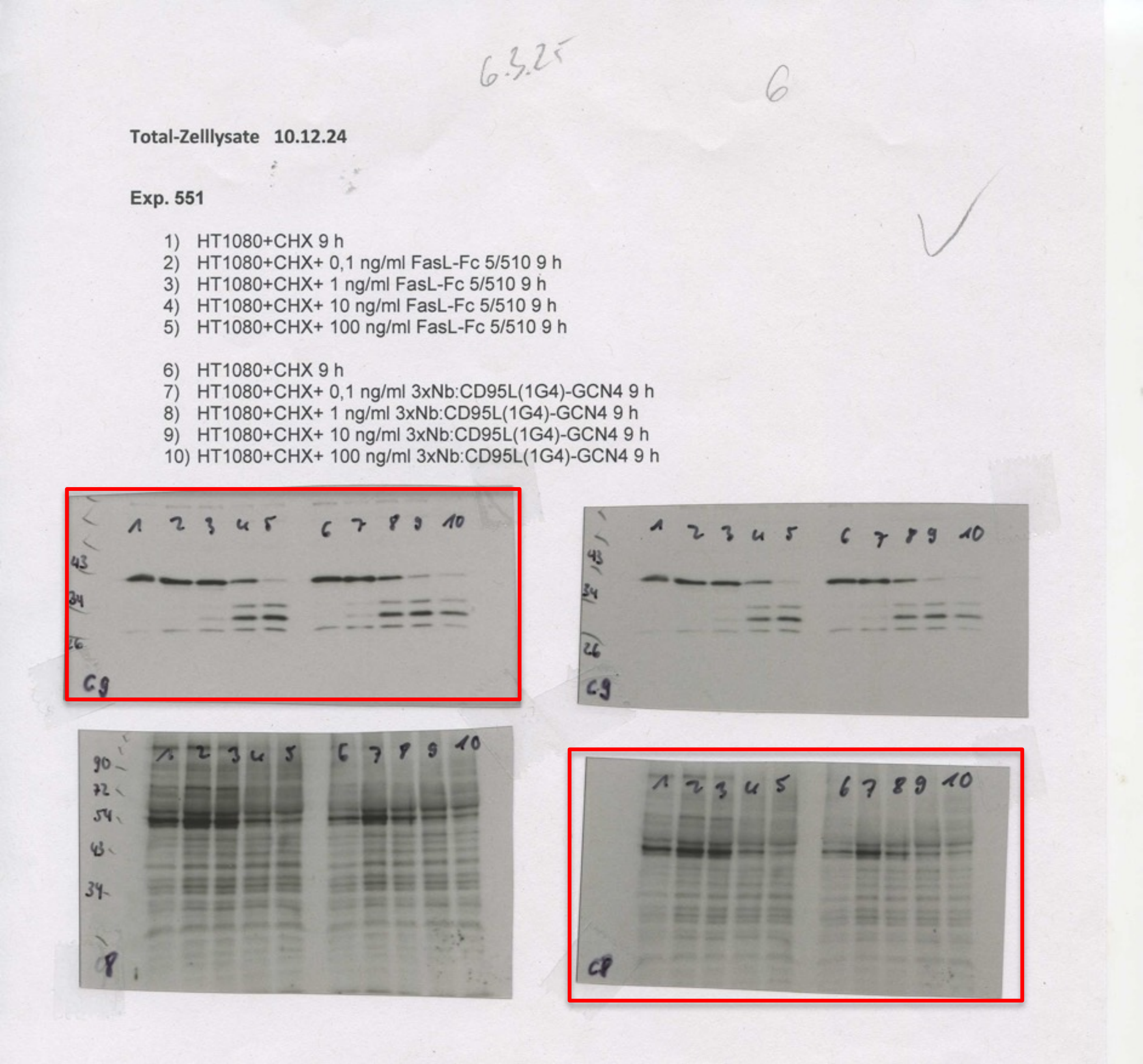
6.3.25
6
Total-Zelllysate 10.12.24
Exp. 551
1) HT1080+CHX 9 h
2) HT1080+CHX+ 0,1 ng/ml FasL-Fc 5/510 9 h
3) HT1080+CHX+ 1 ng/ml FasL-Fc 5/510 9 h
4) HT1080+CHX+ 10 ng/ml FasL-Fc 5/510 9 h
5) HT1080+CHX+ 100 ng/ml FasL-Fc 5/510 9 h
6) HT1080+CHX 9 h
7) HT1080+CHX+ 0,1 ng/ml 3xNb:CD95L(1G4)-GCN4 9 h
8) HT1080+CHX+ 1 ng/ml 3xNb:CD95L(1G4)-GCN4 9 h
9) HT1080+CHX+ 10 ng/ml 3xNb:CD95L(1G4)-GCN4 9 h
10) HT1080+CHX+ 100 ng/ml 3xNb:CD95L(1G4)-GCN4 9 h
1 2 3 4 5 6 7 8 9 10
43
34
26
C9
90
72
54
43
34
C8

09.01.25 7

Total-Zelllysate 10.12.24

Exp. 551

1) HT1080+CHX 9 h
2) HT1080+CHX+ 0,1 ng/ml FasL-Fc 5/510 9 h
3) HT1080+CHX+ 1 ng/ml FasL-Fc 5/510 9 h
4) HT1080+CHX+ 10 ng/ml FasL-Fc 5/510 9 h
5) HT1080+CHX+ 100 ng/ml FasL-Fc 5/510 9 h

6) HT1080+CHX 9 h
7) HT1080+CHX+ 0,1 ng/ml 3xNb:CD95L(1G4)-GCN4 9 h
8) HT1080+CHX+ 1 ng/ml 3xNb:CD95L(1G4)-GCN4 9 h
9) HT1080+CHX+ 10 ng/ml 3xNb:CD95L(1G4)-GCN4 9 h
10) HT1080+CHX+ 100 ng/ml 3xNb:CD95L(1G4)-GCN4 9 h

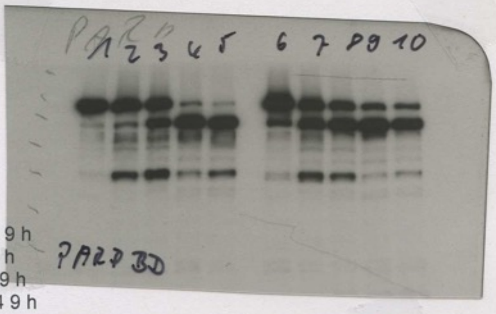

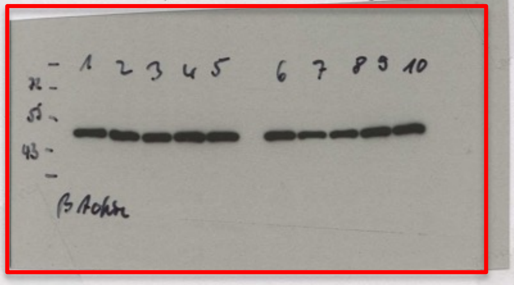

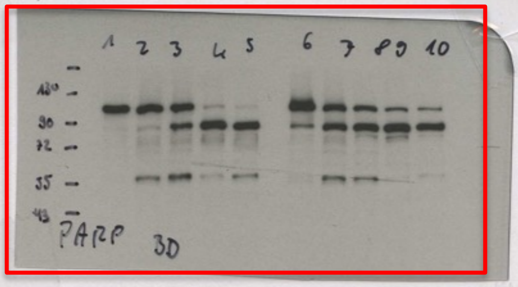

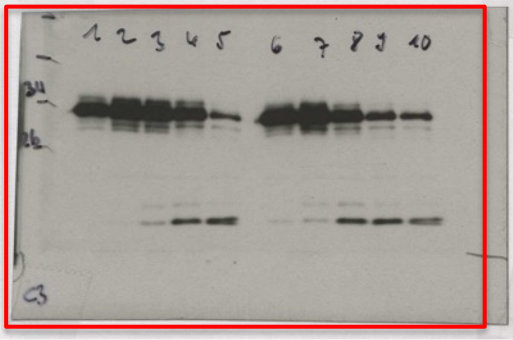

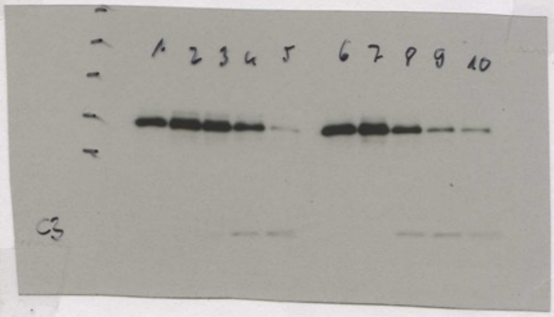

Figure 5E

IP – FADD, Casp8 und FLIP

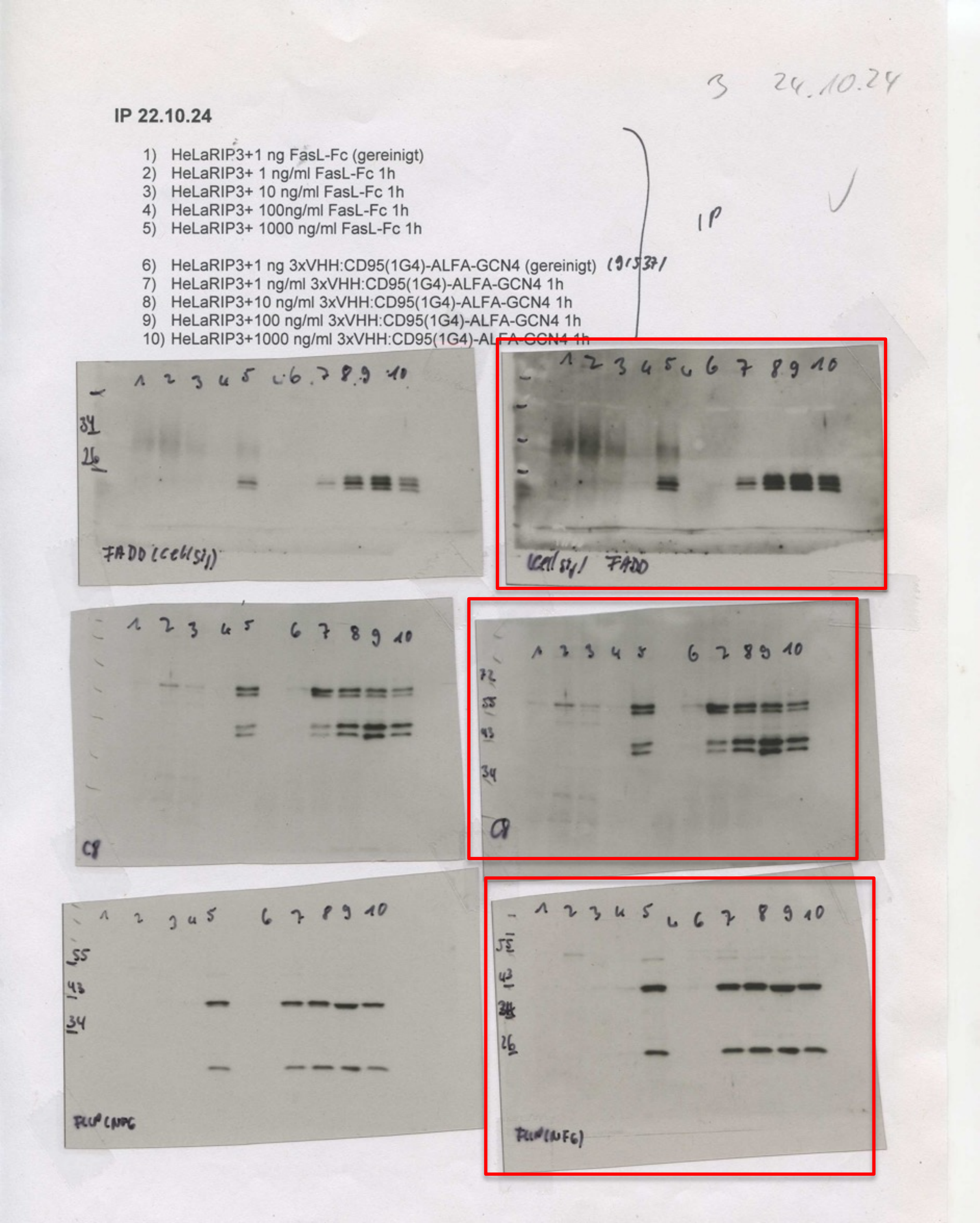

IP 22.10.24

1) HeLaRIP3+1 ng FasL-Fc (gereinigt)
2) HeLaRIP3+ 1 ng/ml FasL-Fc 1h
3) HeLaRIP3+ 10 ng/ml FasL-Fc 1h
4) HeLaRIP3+ 100ng/ml FasL-Fc 1h
5) HeLaRIP3+ 1000 ng/ml FasL-Fc 1h

6) HeLaRIP3+1 ng 3xVHH:CD95(1G4)-ALFA-GCN4 (gereinigt)
7) HeLaRIP3+1 ng/ml 3xVHH:CD95(1G4)-ALFA-GCN4 1h
8) HeLaRIP3+10 ng/ml 3xVHH:CD95(1G4)-ALFA-GCN4 1h
9) HeLaRIP3+100 ng/ml 3xVHH:CD95(1G4)-ALFA-GCN4 1h
10) HeLaRIP3+1000 ng/ml 3xVHH:CD95(1G4)-ALFA-GCN4 1h

IP Fas

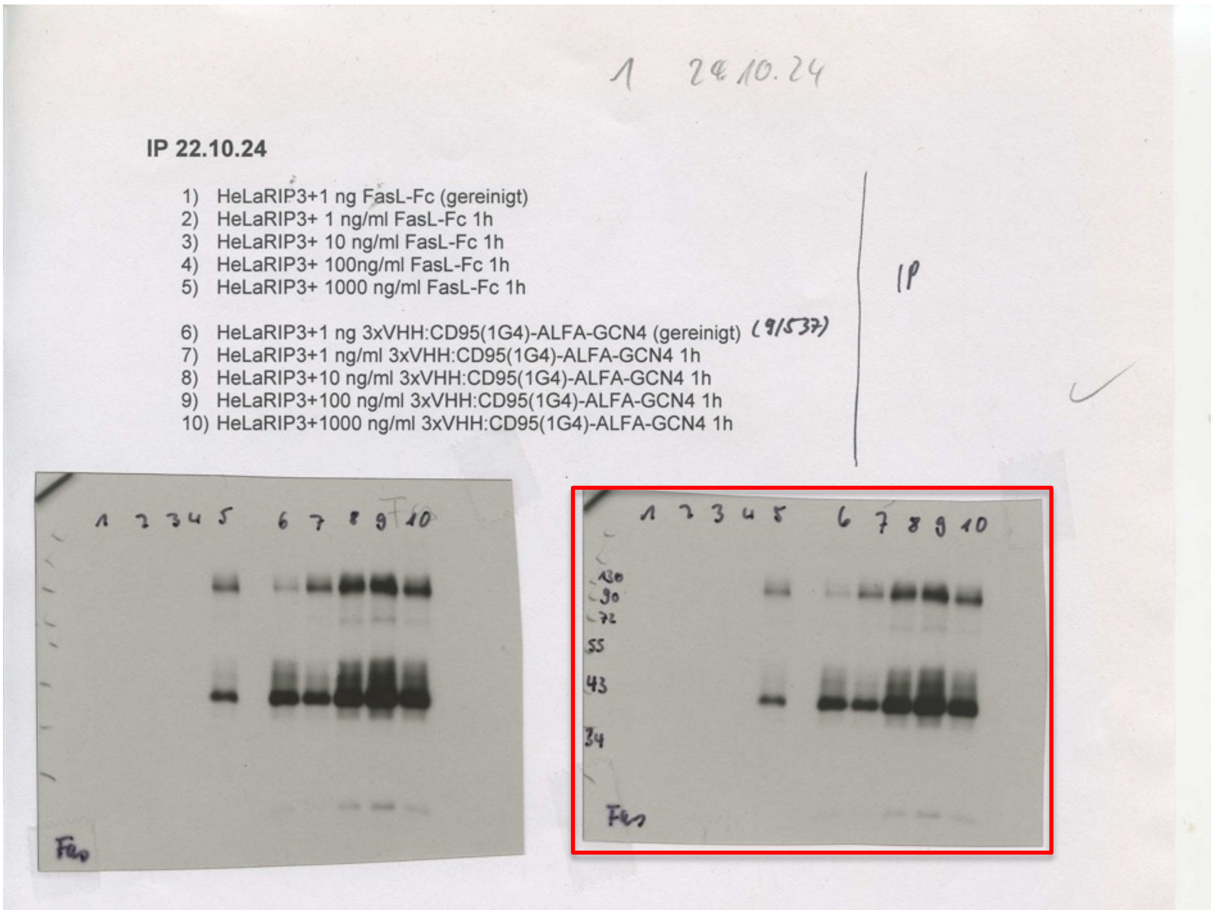
1 24.10.24

IP 22.10.24

1) HeLaRIP3+1 ng FasL-Fc (gereinigt)
2) HeLaRIP3+ 1 ng/ml FasL-Fc 1h
3) HeLaRIP3+ 10 ng/ml FasL-Fc 1h
4) HeLaRIP3+ 100ng/ml FasL-Fc 1h
5) HeLaRIP3+ 1000 ng/ml FasL-Fc 1h

6) HeLaRIP3+1 ng 3xVHH:CD95(1G4)-ALFA-GCN4 (gereinigt) (9/537)
7) HeLaRIP3+1 ng/ml 3xVHH:CD95(1G4)-ALFA-GCN4 1h
8) HeLaRIP3+10 ng/ml 3xVHH:CD95(1G4)-ALFA-GCN4 1h
9) HeLaRIP3+100 ng/ml 3xVHH:CD95(1G4)-ALFA-GCN4 1h
10) HeLaRIP3+1000 ng/ml 3xVHH:CD95(1G4)-ALFA-GCN4 1h

IP

Lys FADD, Casp8 und FLIP

2 24.10.24

IP 22.10.24

1) HeLaRIP3+1 ng FasL-Fc (gereinigt)
2) HeLaRIP3+ 1 ng/ml FasL-Fc 1h
3) HeLaRIP3+ 10 ng/ml FasL-Fc 1h
4) HeLaRIP3+ 100ng/ml FasL-Fc 1h
5) HeLaRIP3+ 1000 ng/ml FasL-Fc 1h

6) HeLaRIP3+1 ng 3xVHH:CD95(1G4)-ALFA-GCN4 (gereinigt)
7) HeLaRIP3+1 ng/ml 3xVHH:CD95(1G4)-ALFA-GCN4 1h
8) HeLaRIP3+10 ng/ml 3xVHH:CD95(1G4)-ALFA-GCN4 1h
9) HeLaRIP3+100 ng/ml 3xVHH:CD95(1G4)-ALFA-GCN4 1h
10) HeLaRIP3+1000 ng/ml 3xVHH:CD95(1G4)-ALFA-GCN4 1h

Lys (915371)

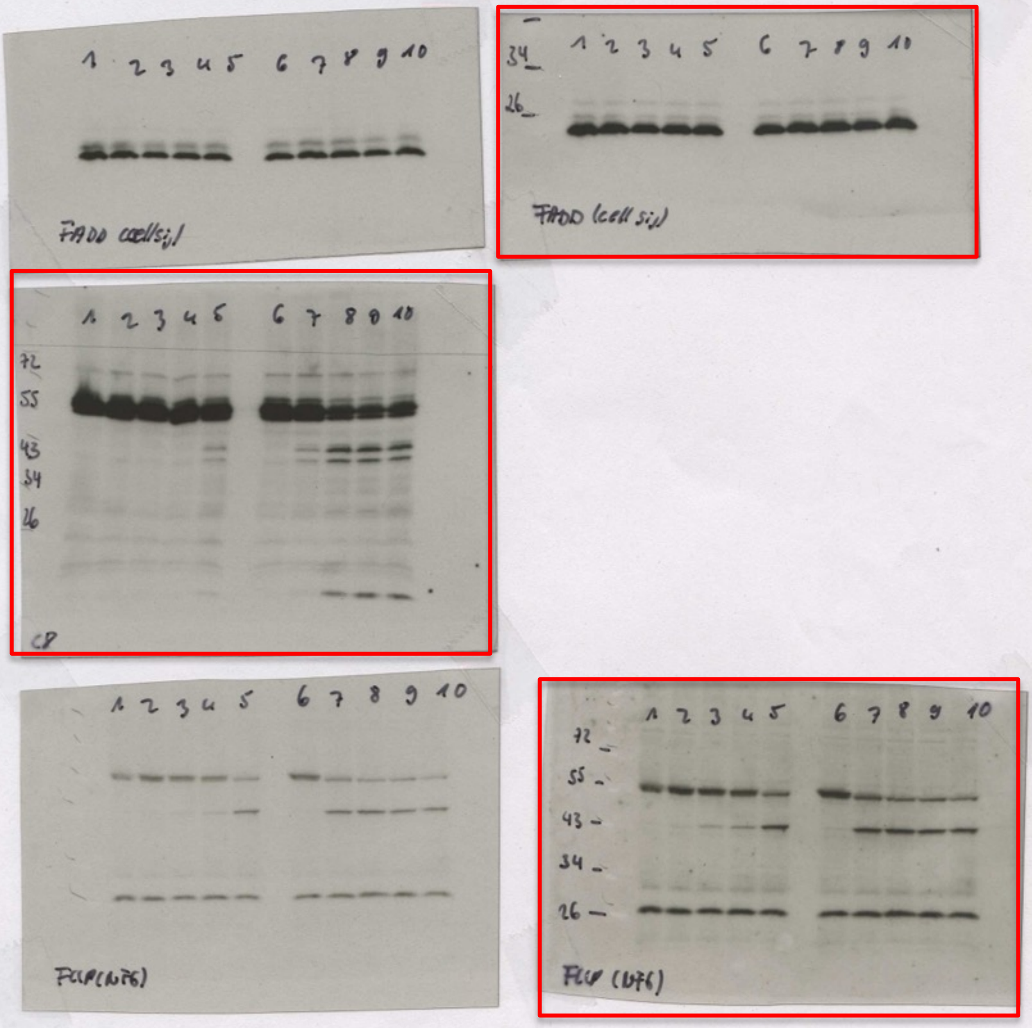

Lys- Fas

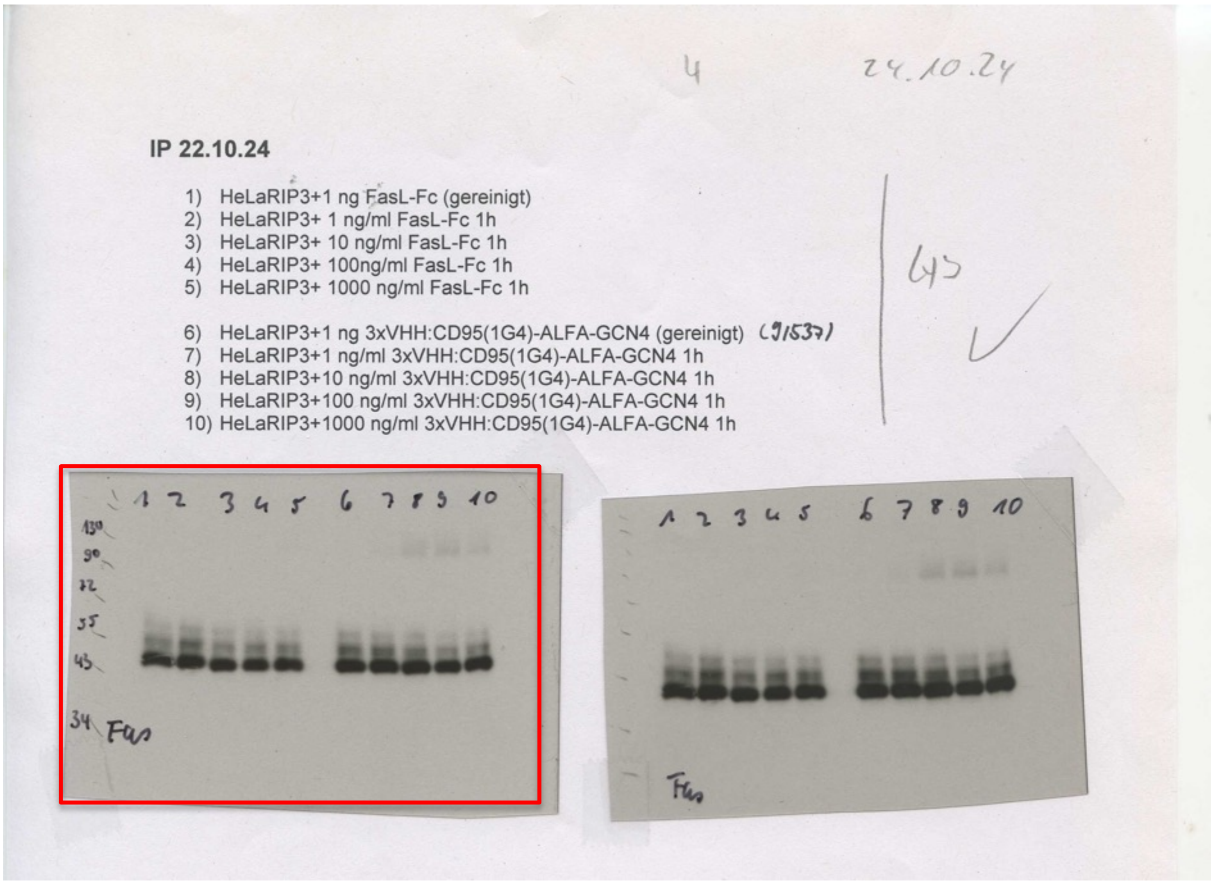

4 24.10.24

IP 22.10.24

1) HeLaRIP3+1 ng FasL-Fc (gereinigt)
2) HeLaRIP3+ 1 ng/ml FasL-Fc 1h
3) HeLaRIP3+ 10 ng/ml FasL-Fc 1h
4) HeLaRIP3+ 100ng/ml FasL-Fc 1h
5) HeLaRIP3+ 1000 ng/ml FasL-Fc 1h

6) HeLaRIP3+1 ng 3xVHH:CD95(1G4)-ALFA-GCN4 (gereinigt) (91537)
7) HeLaRIP3+1 ng/ml 3xVHH:CD95(1G4)-ALFA-GCN4 1h
8) HeLaRIP3+10 ng/ml 3xVHH:CD95(1G4)-ALFA-GCN4 1h
9) HeLaRIP3+100 ng/ml 3xVHH:CD95(1G4)-ALFA-GCN4 1h
10) HeLaRIP3+1000 ng/ml 3xVHH:CD95(1G4)-ALFA-GCN4 1h

Figure 6

Lysate U2OS

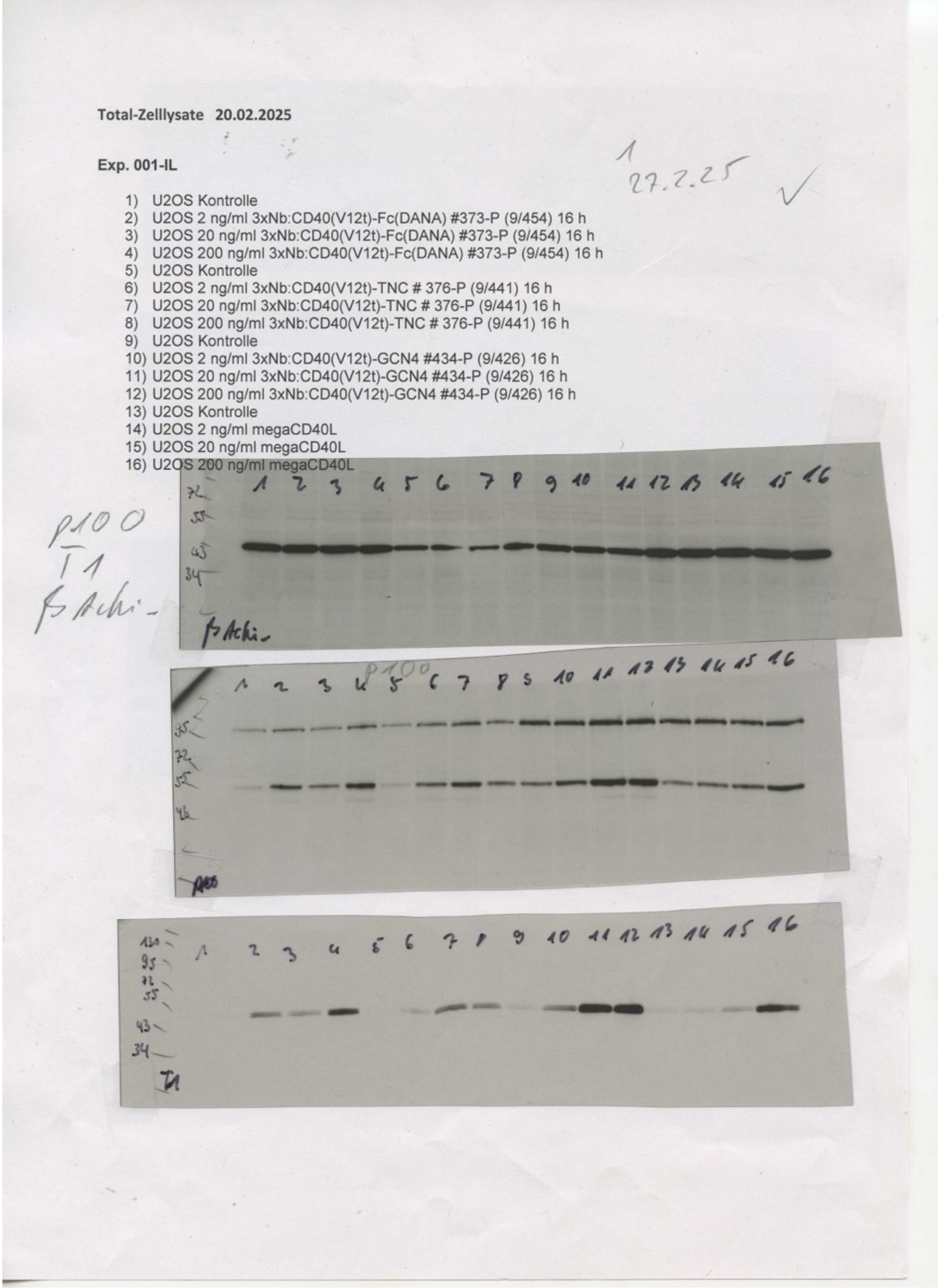

Total-Zelllysate 20.02.2025

Exp. 001-IL

1) U2OS Kontrolle
2) U2OS 2 ng/ml 3xNb:CD40(V12t)-Fc(DANA) #373-P (9/454) 16 h
3) U2OS 20 ng/ml 3xNb:CD40(V12t)-Fc(DANA) #373-P (9/454) 16 h
4) U2OS 200 ng/ml 3xNb:CD40(V12t)-Fc(DANA) #373-P (9/454) 16 h
5) U2OS Kontrolle
6) U2OS 2 ng/ml 3xNb:CD40(V12t)-TNC # 376-P (9/441) 16 h
7) U2OS 20 ng/ml 3xNb:CD40(V12t)-TNC # 376-P (9/441) 16 h
8) U2OS 200 ng/ml 3xNb:CD40(V12t)-TNC # 376-P (9/441) 16 h
9) U2OS Kontrolle
10) U2OS 2 ng/ml 3xNb:CD40(V12t)-GCN4 #434-P (9/426) 16 h
11) U2OS 20 ng/ml 3xNb:CD40(V12t)-GCN4 #434-P (9/426) 16 h
12) U2OS 200 ng/ml 3xNb:CD40(V12t)-GCN4 #434-P (9/426) 16 h
13) U2OS Kontrolle
14) U2OS 2 ng/ml megaCD40L
15) U2OS 20 ng/ml megaCD40L
16) U2OS 200 ng/ml megaCD40L

Lysate HT1080-GITR

12.12.24

1

Total-Zelllysate 04.12.24

Exp. 549

1) HT1080GITR Kontrolle
2) HT1080GITR 2 ng/ml 3xNb:GITR(C06)-Fc-GpL 8/841 16 h
3) HT1080GITR 20 ng/ml 3xNb:GITR(C06)-Fc-GpL 8/841 16 h
4) HT1080GITR 200 ng/ml 3xNb:GITR(C06)-Fc-GpL 8/841 16 h
5) HT1080GITR Kontrolle
6) HT1080GITR 2 ng/ml 3xNb:GITR(C06)-TNC 9/279 16 h
7) HT1080GITR 20 ng/ml 3xNb:GITR(C06)- TNC 9/279 16 h
8) HT1080GITR 200 ng/ml 3xNb:GITR(C06)- TNC 9/279 16 h
9) HT1080GITR Kontrolle
10) HT1080GITR 2 ng/ml 3xNb:GITR(C06)-GCN4 9/505 16 h
11) HT1080GITR 20 ng/ml 3xNb:GITR(C06)- GCN4 9/505 16 h
12) HT1080GITR 200 ng/ml 3xNb:GITR(C06)- GCN4 9/505 16 h
13) HT1080GITR Kontrolle
14) HT1080GITR 2 ng/ml rec. multim. GITRL 16 h
15) HT1080GITR 20 ng/ml rec. multim. GITRL 16 h
16) HT1080GITR 200 ng/ml rec. multim. GITRL 16 h

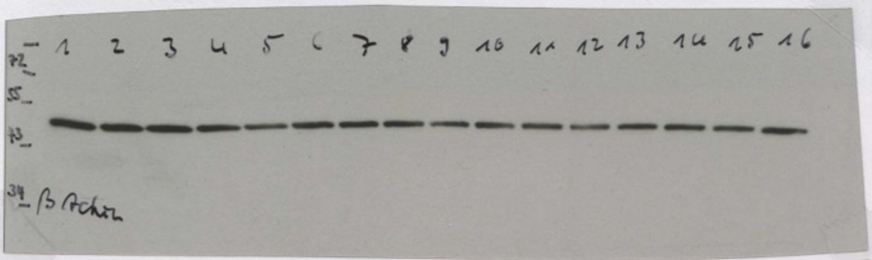

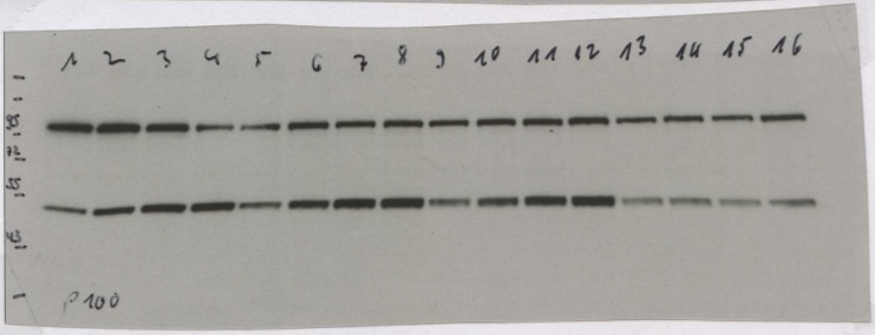

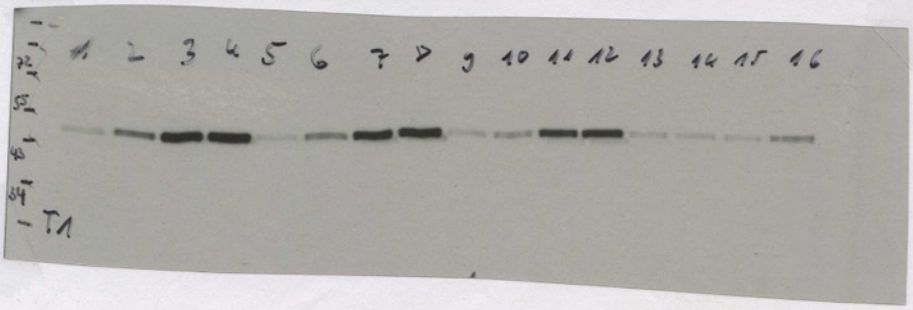

Lysate HT1080-OX40

13.2.25 1

Total-Zelllysate 16.01.25

Exp. 555

1) HT1080OX40 Kontrolle
2) HT1080OX40 2 ng/ml 3xNb:OX40(V1)-Fc(DANA) 8/922 16 h
3) HT1080OX40 20 ng/ml 3xNb: OX40(V1)-Fc(DANA) 8/922 16 h
4) HT1080OX40 200 ng/ml 3xNb: OX40(V1)-Fc(DANA) 8/922 16 h
5) HT1080OX40 Kontrolle
6) HT1080OX40 2 ng/ml 3xNb: OX40(V1)-TNC 9/277 16 h
7) HT1080OX40 20 ng/ml 3xNb: OX40(V1)-TNC 9/277 16 h
8) HT1080OX40 200 ng/ml 3xNb: OX40(V1)-TNC 9/277 16 h
9) HT1080OX40 Kontrolle
10) HT1080OX40 2 ng/ml 3xNb: OX40(V1)-GCN4 9/504 16 h
11) HT1080OX40 20 ng/ml 3xNb: OX40(V1)-GCN4 9/504 16 h
12) HT1080OX40 200 ng/ml 3xNb: OX40(V1)-GCN4 9/504 16 h
13) HT1080OX40 Kontrolle
14) HT1080OX40 2 ng/mlFc:hOX40L 16 h
15) HT1080OX40 20 ng/mlFc:hOX40L 16 h
16) HT1080OX40 200 ng/mlFc:hOX40L 16 h

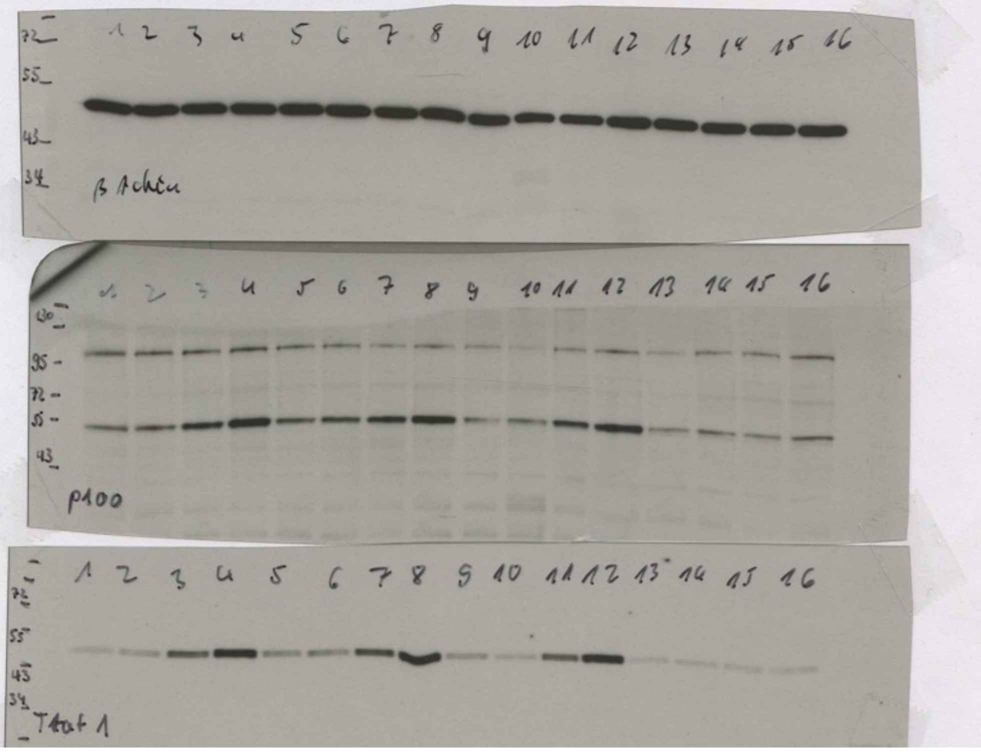

Supplemental Figure S2

Westernblott-Nr: 005MS

Datum: 13.02.26

1 AK: Direct-Blot HRP anti-DYKDDDDK Tag (1:50.000) + anti-ALFA-HRP (1:100.000)

Spur	Konstrukt			Volumen µl	Konzent-ration µg/ml
1	8/841	anti-GITR(hzC06v1.1)-VHH(3x)-Fc-Flag-Gaussia(w/o)-pCR3	13/22	10	
2	8/841	anti-GITR(hzC06v1.1)-VHH(3x)-Fc-Flag-Gaussia(w/o)-pCR3 - non reducing	13/22	10	
3	9/279	anti-GITR(hzC06v1.1)-VHH(3x)-Flag-TNC-pCR3	13/25	10	
4	9/279	anti-GITR(hzC06v1.1)-VHH(3x)-Flag-TNC-pCR3 - non reducing	13/25	10	
5	9/505	anti-GITR(hzC06v1.1)-VHH(3x)-ALFAtag-GCN4-pCR3	13/28	10	
6	9/505	anti-GITR(hzC06v1.1)-VHH(3x)-ALFAtag-GCN4-pCR3 - non reducing	13/28	10	
7					
8	8/922	anti-OX40(V1)-VHH(3x)-Fc(DANA)-Flag-pCR3	13/31	15	
9	8/922	anti-OX40(V1)-VHH(3x)-Fc(DANA)-Flag-pCR3 - non reducing	13/31	15	
10	9/277	anti-OX40(V1)-VHH(3x)-Flag-TNC-pCR3	13/34	10	
11	9/277	anti-OX40(V1)-VHH(3x)-Flag-TNC-pCR3 - non reducing	13/34	10	
12	9/504	anti-OX40(V1)-VHH(3x)-ALFAtag-GCN4-pCR3	13/37	10	
13	9/504	anti-OX40(V1)-VHH(3x)-ALFAtag-GCN4-pCR3 - non reducing	13/37	10	
14					
15	Standard 9/119 ALFA-Flag-TNC-scTNF80(mu)-pCR3			10	200ng
16				5	100ng
17				2,5	50ng
18					
19					

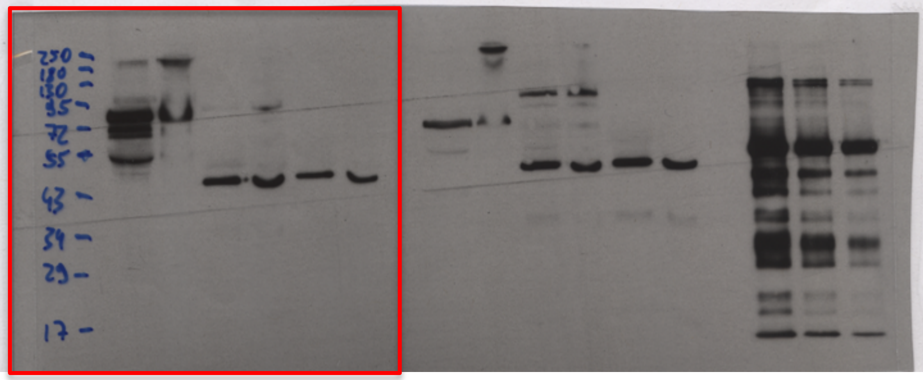

Westernblott-Nr: 605MS

Datum: 19.11.25

1 AK: Direct-Blot HRP anti-DYKDDDDK Tag (1:50.000) + anti-ALFA-HRP (1:75.000)

Spur	Konstrukt				Volumen	Konzent-ration
					µl	µg/ml
1	8/841	anti-GITR(hzC06v1.1)-VHH(3x)-Fc-Flag-Gaussia(w/o)-pCR3	13/22	V	10	
2	8/841	anti-GITR(hzC06v1.1)-VHH(3x)-Fc-Flag-Gaussia(w/o)-pCR3 - non reducing		V	10	
3	9/279	anti-GITR(hzC06v1.1)-VHH(3x)-Flag-TNC-pCR3	13/25	V	10	
4	9/279	anti-GITR(hzC06v1.1)-VHH(3x)-Flag-TNC-pCR3 - non reducing		V	10	
5	9/505	anti-GITR(hzC06v1.1)-VHH(3x)-ALFAtag-GCN4-pCR3	13/28	V	10	
6	9/505	anti-GITR(hzC06v1.1)-VHH(3x)-ALFAtag-GCN4-pCR3 - non reducing		V	10	
7						
8	8/922	anti-OX40(V1)-VHH(3x)-Fc(DANA)-Flag-pCR3	13/31	V	10	
9	8/922	anti-OX40(V1)-VHH(3x)-Fc(DANA)-Flag-pCR3 - non reducing		V	10	
10	9/277	anti-OX40(V1)-VHH(3x)-Flag-TNC-pCR3	13/34	V	10	
11	9/277	anti-OX40(V1)-VHH(3x)-Flag-TNC-pCR3 - non reducing		V	10	
12	9/504	anti-OX40(V1)-VHH(3x)-ALFAtag-GCN4-pCR3	13/37	V	10	
13	9/504	anti-OX40(V1)-VHH(3x)-ALFAtag-GCN4-pCR3 - non reducing		V	10	
14						
15	Standard 9/119 ALFA-Flag-TNC-scTNF80(mu)-pCR3					200ng
16						100ng
17						50ng
18						
19						

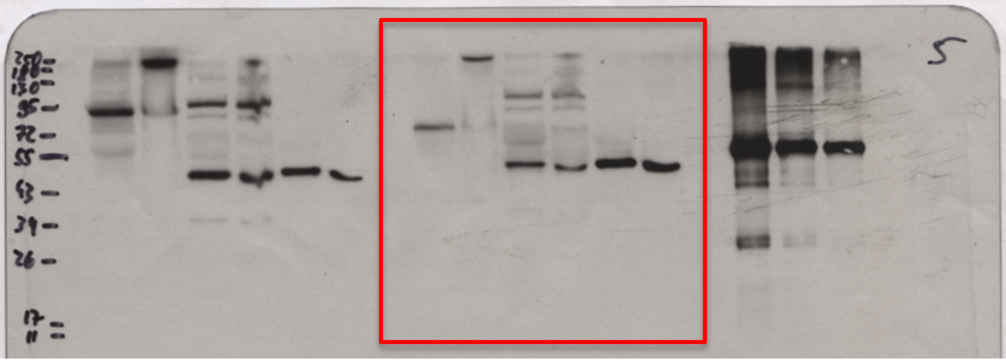

Westernblott-Nr:006 MS Datum: 19.11.25

1 AK: Direct-Blot HRP anti-DYKDDDDK Tag (1:50.000) + anti-ALFA-HRP (1:75.000)

Spur		Konstrukt			Volumen µl	Konzentration µg/ml
1	8/819	anti-41BB-VHH(3x)-Fc(DANA)-Flag-pCR3	14/2	V	10 15	
2	8/819	anti-41BB-VHH(3x)-Fc(DANA)-Flag-pCR3 - non reducing		V	10	
3	9/278	anti-41BB-VHH(3x)-Flag-TNC-pCR3	14/5	V	10	
4	9/278	anti-41BB-VHH(3x)-Flag-TNC-pCR3 - non reducing		V	10	
5	9/427	anti-41BB-VHH(3x)-GCN4-Flag-pCR3	14/8	V	10	
6	9/427	anti-41BB-VHH(3x)-GCN4-Flag-pCR3 - non reducing		V	10	
7						
8	9/454	anti-CD40(V12t)-VHH(3x)-ALFAtag-Fc(DANA)-pCR3 (Flagless)	13/40	V	10 5	
9	9/454	anti-CD40(V12t)-VHH(3x)-ALFAtag-Fc(DANA)-pCR3 (Flagless) - non reducing		V	10	
10	9/441	anti-CD40(V12t)-VHH(3x)-ALFAtag-TNC-pCR3 (Flagless)	13/43	V	10	
11	9/441	anti-CD40(V12t)-VHH(3x)-ALFAtag-TNC-pCR3 (Flagless) - non reducing		V	10	
12	9/452	anti-CD40(V12t)-VHH(3x)-ALFAtag-GCN4-pCR3 (Flagless)	13/46	V	10	
13	9/452	anti-CD40(V12t)-VHH(3x)-ALFAtag-GCN4-pCR3 (Flagless) - non reducing		V	10	
14						
15	Standard 9/119 ALFA-Flag-TNC-scTNF80(mu)-pCR3					200ng
16						100ng
17						50ng
18						
19						

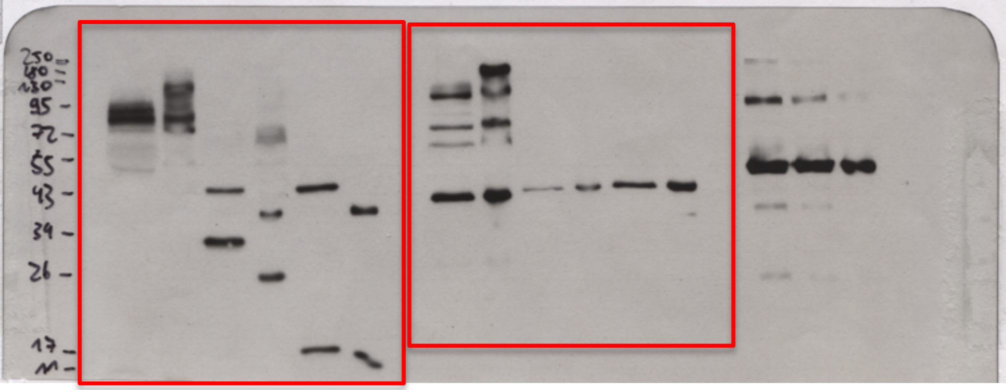

Westernblott-Nr.: 007 MS

Datum: 19.02.26

1 AK: Direct-Blot HRP anti-DYKDDDDK Tag (1:50.000) + anti-ALFA-HRP (1:100.000)

Spur	Konstrukt			Volumen	Konzent-ration
				µl	µg/ml
1	8/800	C188-VHH(3x)-Fc(DANA)-Flag-pCR3 (neu)	14/11	10	
2	8/800	C188-VHH(3x)-Fc(DANA)-Flag-pCR3 (neu) - non reducing	14/11	10	
3	8/849	C188-VHH(3x)-Flag-TNC-pCR3	14/14	7,5	
4	8/849	C188-VHH(3x)-Flag-TNC-pCR3 - non reducing	14/1~~6~~4	7,5	
5	9/563	C188-VHH(3x)-ALFAtag-GCN4-pCR3	14/17	7,5	
6	9/563	C188-VHH(3x)-ALFAtag-GCN4-pCR3 - non reducing	14/1~~8~~7	7,5	
7					
8	9/113	anti-BCMA(269A37948)-VHH(3x)-Fc(DANA)-Flag-pCR3	14/20	7,5	
9	9/113	anti-BCMA(269A37948)-VHH(3x)-Fc(DANA)-Flag-pCR3 - non reducing	14/20	7,5	
10	9/125	anti-BCMA(269A37948)-VHH(3x)-Flag-TNC-pCR3	14/23	10	
11	9/125	anti-BCMA(269A37948)-VHH(3x)-Flag-TNC-pCR3 - non reducing	14/23	10	
12	9/510	anti-BCMA(269A37948)-VHH(3x)-ALFAtag-GCN4-pCR3	14/26	10	
13	9/510	anti-BCMA(269A37948)-VHH(3x)-ALFAtag-GCN4-pCR3 - non reducing	14/26	10	
14					
15	Standard 9/119 ALFA-Flag-TNC-scTNF80(mu)-pCR3			10	200ng
16				5	100ng
17				2,5	50ng
18					
19					

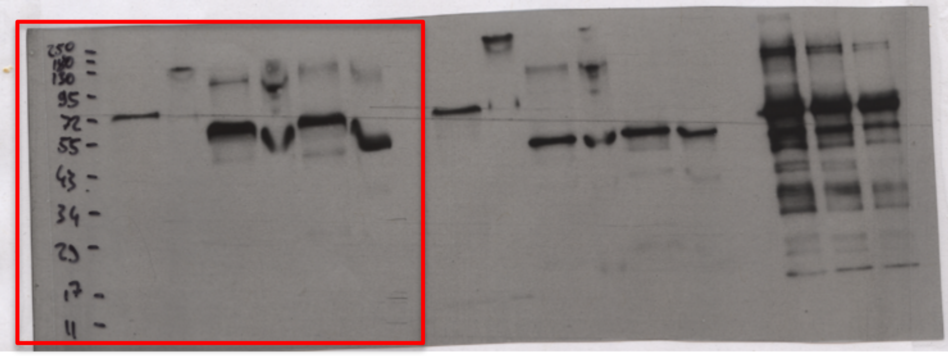

Westernblott-Nr: 007 MS

Datum: 19.11.25

1 AK: Direct-Blot HRP anti-DYKDDDDK Tag (1:50.000) + anti-ALFA-HRP (1:75.000)

Spur	Konstrukt				Volumen	Konzent-ration
					µl	µg/ml
1	8/800	C188-VHH(3x)-Fc(DANA)-Flag-pCR3 (neu)	14/11	V	10	
2	8/800	C188-VHH(3x)-Fc(DANA)-Flag-pCR3 (neu) - non reducing		V	10	
3	8/849	C188-VHH(3x)-Flag-TNC-pCR3	14/14	V	10	
4	8/849	C188-VHH(3x)-Flag-TNC-pCR3 - non reducing		V	10	
5	9/563	C188-VHH(3x)-ALFAtag-GCN4-pCR3	14/17	V	10	
6	9/563	C188-VHH(3x)-ALFAtag-GCN4-pCR3 - non reducing		V	10	
7						
8	9/113	anti-BCMA(269A37948)-VHH(3x)-Fc(DANA)-Flag-pCR3	14/20	V	10	
9	9/113	anti-BCMA(269A37948)-VHH(3x)-Fc(DANA)-Flag-pCR3 - non reducing		V	10	
10	9/125	anti-BCMA(269A37948)-VHH(3x)-Flag-TNC-pCR3	14/23	V	10	
11	9/125	anti-BCMA(269A37948)-VHH(3x)-Flag-TNC-pCR3 - non reducing		V	10	
12	9/510	anti-BCMA(269A37948)-VHH(3x)-ALFAtag-GCN4-pCR3	14/26	V	10	
13	9/510	anti-BCMA(269A37948)-VHH(3x)-ALFAtag-GCN4-pCR3 - non reducing		V	10	
14						
15	Standard 9/119 ALFA-Flag-TNC-scTNF80(mu)-pCR3					200ng
16						100ng
17						50ng
18						
19						

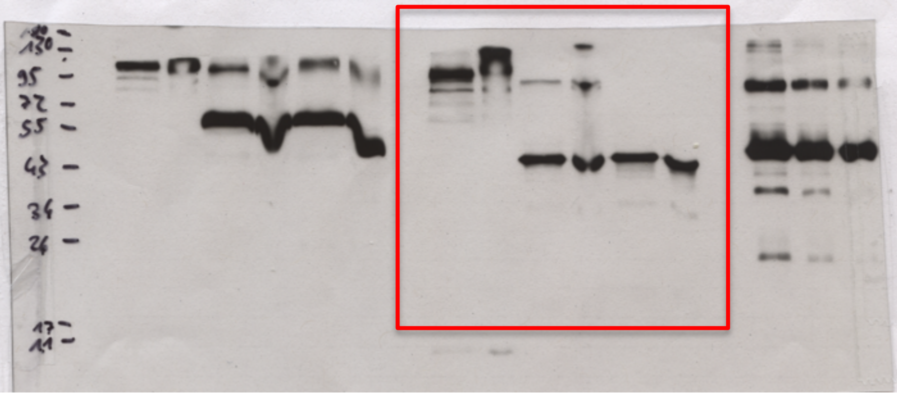

Westernblott-Nr: 108MS

Datum: 19.02.26

1 AK: Direct-Blot HRP anti-DYKDDDDK Tag (1:50.000) + anti-ALFA-HRP (1:100.000)

Spur	Konstrukt			Volumen	Konzent-ration
				µl	µg/ml
1	9/239	anti-TNFR1(NB70)-VHH(3x)-Fc-Flag-Gaussia(w/o)-pCR3	14/29	7,5	
2	9/239	anti-TNFR1(NB70)-VHH(3x)-Fc-Flag-Gaussia(w/o)-pCR3 - non reducing	14/29	7,5	
3	9/496	anti-TNFR1(NB70)-VHH(3x)-Flag-TNC-pCR3	14/32	10	
4	9/496	anti-TNFR1(NB70)-VHH(3x)-Flag-TNC-pCR3 - non reducing	14/32	10	
5	9/511	anti-TNFR1(NB70)-VHH(3x)-ALFAtag-GCN4-pCR3	14/35	10	
6	9/511	anti-TNFR1(NB70)-VHH(3x)-ALFAtag-GCN4-pCR3 - non reducing	14/35	10	
7					
8	9/111	anti-DR5(F5)-VHH(3x)-Fc(DANA)-Flag-pCR3	14/38	15	
9	9/111	anti-DR5(F5)-VHH(3x)-Fc(DANA)-Flag-pCR3 - non reducing	14/38	15	
10	9/127	anti-DR5(F5)-VHH(3x)-Flag-TNC-pCR3	14/41	15	
11	9/127	anti-DR5(F5)-VHH(3x)-Flag-TNC-pCR3 - no reducing	14/41	15	
12	9/564	anti-DR5(F5)-VHH(3x)-Flag-GCN4-pCR3	14/44	17,5	
13	9/564	anti-DR5(F5)-VHH(3x)-Flag-GCN4-pCR3 - non reducing	14/44	17,5	
14					
15	Standard 9/119 ALFA-Flag-TNC-scTNF80(mu)-pCR3			10	200ng
16				5	100ng
17				2,5	50ng
18					
19					

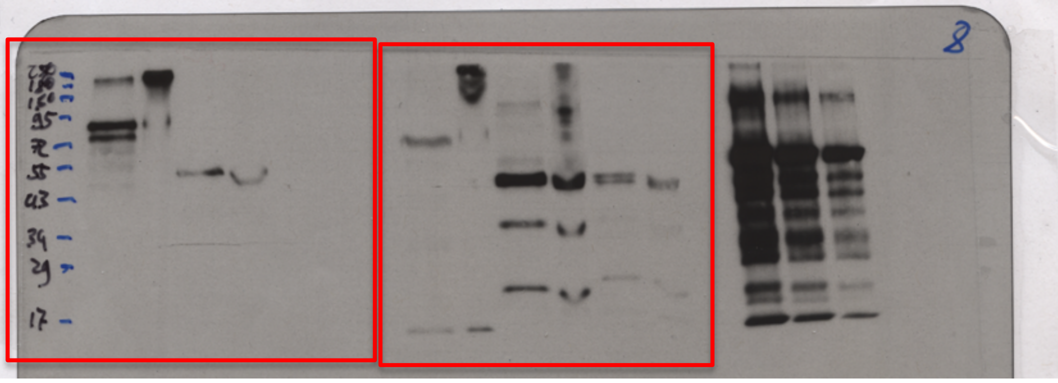

Westernblott-Nr: 009 MS

Datum: 19.11.25

1 AK: Direct-Blot HRP anti-DYKDDDDK Tag (1:50.000) + anti-ALFA-HRP (1:75.000)

Spur	Konstrukt				Volumen	Konzent-ration
					µl	µg/ml
1	9/270	anti-TNFR1(mu)-VHH(3x)-Fc-Flag-Gaussia(w/o)-pCR3	14/47	V	15 20	
2	9/270	anti-TNFR1(mu)-VHH(3x)-Fc-Flag-Gaussia(w/o)-pCR3 - non reducing		V	15	
3	9/507	anti-TNFR1(mu)-VHH(3x)-Flag-TNC-pCR3	14/50	V	15	
4	9/507	anti-TNFR1(mu)-VHH(3x)-Flag-TNC-pCR3 - non reducing		V	15	
5	9/512	anti-TNFR1(mu)-VHH(3x)-ALFAtag-GCN4-pCR3	15/3	V	15	
6	9/512	anti-TNFR1(mu)-VHH(3x)-ALFAtag-GCN4-pCR3 - non reducing		V	15	
7						
8	9/544	anti-CD95(1G4-63-92)-VHH(3x)-ALFAtag-Fc(DANA)-pCR3	15/6	V	5	
9	9/544	anti-CD95(1G4-63-92)-VHH(3x)-ALFAtag-Fc(DANA)-pCR3 - non reducing		V	5	
10	9/538	anti-CD95(1G4-63-92)-VHH(3x)-ALFAtag-TNC-pCR3	15/9	V	5	
11	9/538	anti-CD95(1G4-63-92)-VHH(3x)-ALFAtag-TNC-pCR3 - non reducing		V	5	
12	9/537	anti-CD95(1G4-63-92)-VHH(3x)-ALFAtag-GCN4-pCR3	15/12	V	5	
13	9/537	anti-CD95(1G4-63-92)-VHH(3x)-ALFAtag-GCN4-pCR3 - non reducing		V	5	
14						
15	Standard 9/119 ALFA-Flag-TNC-scTNF80(mu)-pCR3					200ng
16						100ng
17						50ng
18						
19						

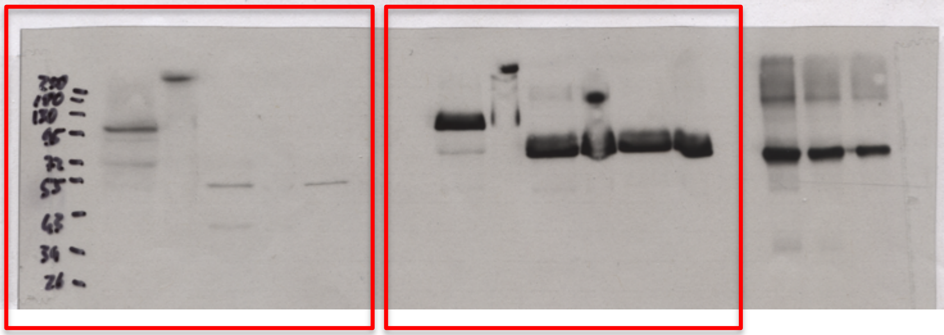

Westernblott-Nr: 010MS

Datum: 19.02.26

1 AK: Direct-Blot HRP anti-DYKDDDDK Tag (1:50.000) + anti-ALFA-HRP (1:100.000)

Spur	Konstrukt			Volumen µl	Konzent-ration µg/ml
1	9/248	anti-CD95(mu)(1A3-10-19)-VHH(3x)-Fc-Flag-Gaussia(w/o)-pCR3	15/18	15	
2	9/248	anti-CD95(mu)(1A3-10-19)-VHH(3x)-Fc-Flag-Gaussia(w/o)-pCR3 - non reducing	15/18	15	
3	9/494	anti-CD95(mu)(1A3-10-19)-VHH(3x)-ALFAtag-TNC-pCR3	15/21	10	
4	9/494	anti-CD95(mu)(1A3-10-19)-VHH(3x)-ALFAtag-TNC-pCR3 - non reducing	15/21	10	
5	9/495	anti-CD95(mu)(1A3-10-19)-VHH(3x)-ALFAtag-GCN4-pCR3	15/24	10	
6	9/495	anti-CD95(mu)(1A3-10-19)-VHH(3x)-ALFAtag-GCN4-pCR3 - non reducing	15/24	10	
7					
8	8/917	anti-CD40(mu)(JPP-G1)-VHH(3x)-Fc(DANA)-Flag-pCR3	15/27	15	
9	8/917	anti-CD40(mu)(JPP-G1)-VHH(3x)-Fc(DANA)-Flag-pCR3 - non reducing	15/27	15	
10	8/944	anti-CD40(mu)(JPP-G1)-VHH(3x)-Flag-TNC-pCR3	15/30	5	
11	8/944	anti-CD40(mu)(JPP-G1)-VHH(3x)-Flag-TNC-pCR3 - non reducing	15/30	5	
12	9/509	anti-CD40(mu)(JPP-G1)-VHH(3x)-ALFAtag-GCN4-pCR3	15/33	5	
13	9/509	anti-CD40(mu)(JPP-G1)-VHH(3x)-ALFAtag-GCN4-pCR3	15/33	5	
14					
15	Standard 9/119 ALFA-Flag-TNC-scTNF80(mu)-pCR3			10	200ng
16				5	100ng
17				2,5	50ng
18					
19					

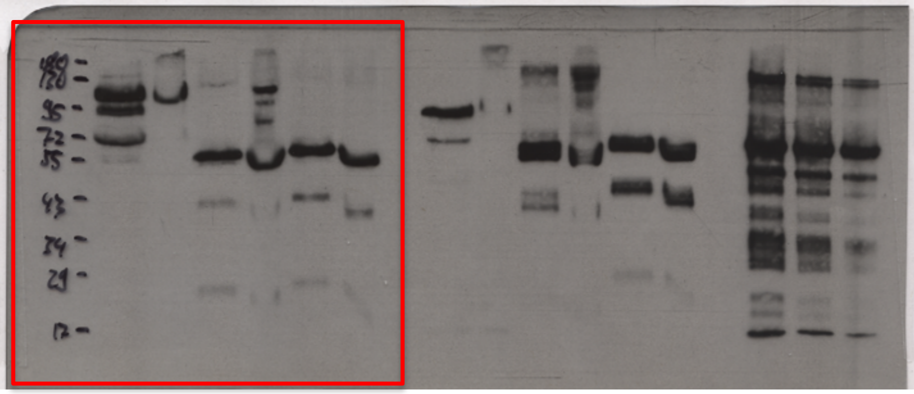

Westernblott-Nr: 010 MS

Datum: 19.11.25

1 AK: Direct-Blot HRP anti-DYKDDDDK Tag (1:50.000) + anti-ALFA-HRP (1:75.000)

Spur		Konstrukt			Volumen	Konzent-ration
					µl	µg/ml
1	9/248	anti-CD95(mu)(1A3-10-19)-VHH(3x)-Fc-Flag-Gaussia(w/o)-pCR3	15/18	V	10	
2	9/248	anti-CD95(mu)(1A3-10-19)-VHH(3x)-Fc-Flag-Gaussia(w/o)-pCR3 - non reducing		V	10	
3	9/494	anti-CD95(mu)(1A3-10-19)-VHH(3x)-ALFAtag-TNC-pCR3	15/21	V	10	
4	9/494	anti-CD95(mu)(1A3-10-19)-VHH(3x)-ALFAtag-TNC-pCR3 - non reducing		V	10	
5	9/495	anti-CD95(mu)(1A3-10-19)-VHH(3x)-ALFAtag-GCN4-pCR3	15/24	V	10	
6	9/495	anti-CD95(mu)(1A3-10-19)-VHH(3x)-ALFAtag-GCN4-pCR3 - non reducing		V	10	
7						
8	8/917	anti-CD40(mu)(JPP-G1)-VHH(3x)-Fc(DANA)-Flag-pCR3	15/27	V	10	
9	8/917	anti-CD40(mu)(JPP-G1)-VHH(3x)-Fc(DANA)-Flag-pCR3 - non reducing		V	10	
10	8/944	anti-CD40(mu)(JPP-G1)-VHH(3x)-Flag-TNC-pCR3	15/30	V	10	
11	8/944	anti-CD40(mu)(JPP-G1)-VHH(3x)-Flag-TNC-pCR3 - non reducing		V	10	
12	9/509	anti-CD40(mu)(JPP-G1)-VHH(3x)-ALFAtag-GCN4-pCR3	15/33	V	10	
13	9/509	anti-CD40(mu)(JPP-G1)-VHH(3x)-ALFAtag-GCN4-pCR3		V	10	
14						
15	Standard 9/119 ALFA-Flag-TNC-scTNF80(mu)-pCR3					200ng
16						100ng
17						50ng
18						
19						

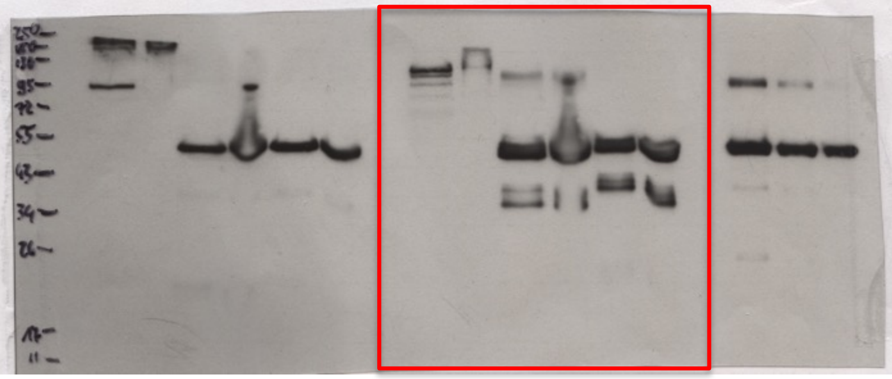

Supplemental Figure S3

Nb:C238

Westernblot-Nr. 39

Datum 01.07.24

1 AK: Anti-Flag-M2 (1:10 000)

2 AK: anti-mouseAK + anti-ALFA (1:100 000)

	Konstrukt		Volume µl	Menge
1	1/466	C238-VHH-Fc-Flag-Gaussia(w/o)-pCR3 (TWIST)	10	
2	8/801	C238-VHH(3x)-Fc(DANA)-Flag-pCR3	10	
3	9/497	C238-VHH(3x)-Flag-TNC-pCR3	10	
4	9/541	C238-VHH(3x)-ALFA-GCN4-pCR3	10	
5				
6	5/458	TNF(wt)-Flag-pCR3	10	
7	7/605	TNF(wt)-Flag-Fc-pCR3 (hIgG-Leader)	10	
8				
9	Standard ALFA 9/452			200ng
10	Standard ALFA 9/452			100ng
11	Standard Flag 6/642 (20 µg/ml)			200 ng
12	Standard Flag 6/642 (20 µg/ml)			100 ng
13	1/466	C238-VHH-Fc-Flag-Gaussia(w/o)-pCR3 (TWIST)		100 ng
14	8/801	C238-VHH(3x)-Fc(DANA)-Flag-pCR3		100 ng
15	9/497	C238-VHH(3x)-Flag-TNC-pCR3		100 ng
16	9/541	C238-VHH(3x)-ALFA-GCN4-pCR3		100 ng
17				
18		TNF80 murin		100 ng
19		TNF80-single-chain-Flag-TNC		100 ng

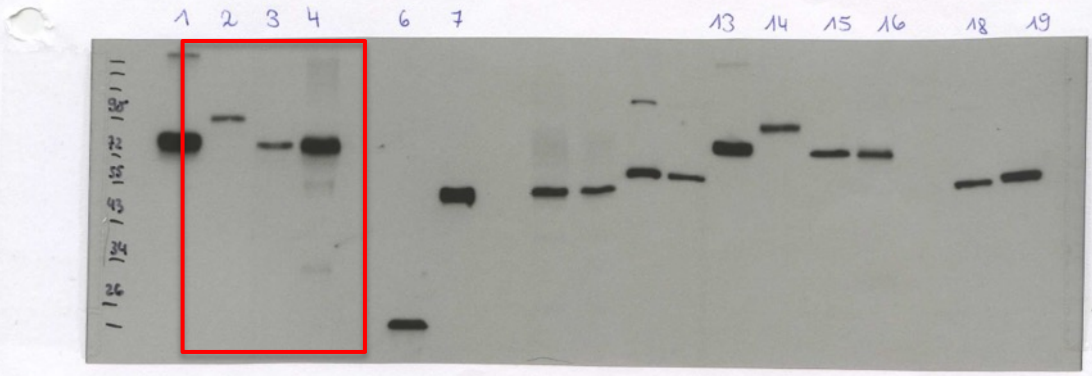

Nb:1B6

Westernblot-Nr. 30

Datum 17.06.24

1 AK: Anti-Flag-M2 (1:10 000)

2 AK: anti-mouseAK + anti-ALFA (1:100 000)

	Konstrukt	Volume µl	Menge
1	9/257 anti-CD40(1B6-13-14-128)-VHH-Fc-Flag-Gaussia(w/o)-pCR3	10 µl	
2	9/478 anti-CD40(1B6-13-14-128)-VHH(3x)-ALFA-Fc(DANA)-pCR3	10 µl	
3	9/476 anti-CD40(1B6-13-14-128)-VHH(3x)-ALFA-TNC-pCR3	10 µl	
4	9/477 anti-CD40(1B6-13-14-128)-VHH(3x)-ALFA-GCN4-pCR3	10 µl	
5			
6	6/651 CD40L-Gaussia-Flag-TNC-pCR3	10 µl	
7	6/144 CD40L-Fc-Flag-pCR3	10 µl	
8			
9	Standard ALFA 9/452		200 ng
[illegible]0	Standard ALFA 9/452		100ng
11	Standard Flag 6/642 (20 µg/ml)		100ng
12	Standard Flag 6/642 (20 µg/ml)		200ng
13	8/819 1) anti-41BB-VHH(3x)-Fc(DANA)-Flag-pCR3	10 µl	5 µg/ml
14	8/819 2) anti-41BB-VHH(3x)-Fc(DANA)-Flag-pCR3	10 µl	5
15	8/819 3) anti-41BB-VHH(3x)-Fc(DANA)-Flag-pCR3	10 µl	2,5
16	8/819 4) anti-41BB-VHH(3x)-Fc(DANA)-Flag-pCR3	10 µl	7,5
17	8/819 5) anti-41BB-VHH(3x)-Fc(DANA)-Flag-pCR3	10 µl	7,5
18	9/278 1) anti-41BB-VHH(3x)-Flag-TNC-pCR3	10 µl	30
19	9/278 2) anti-41BB-VHH(3x)-Flag-TNC-pCR3	10 µl	30

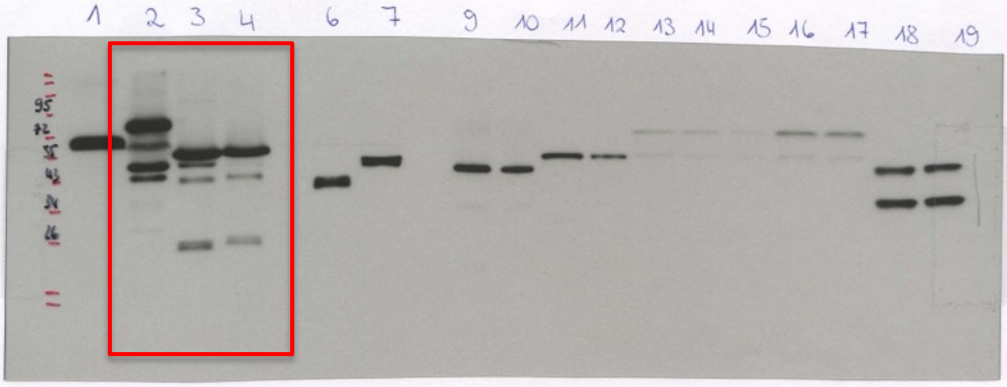

Supplement: Supplementary file 2 — Supplemental material 2 - original data [file 41419_2026_8911_MOESM2_ESM.pdf]
